# Supplementary material for: Three open questions in polygenic score portability
Source: Nat Commun. 2026 Jan 26;17:942. doi: 10.1038/s41467-026-68565-3 (PMC12835231; doi:10.1038/s41467-026-68565-3)
Supplement: Supplementary file 1 — Supplementary Information [file 41467_2026_68565_MOESM1_ESM.pdf]

Supplementary Materials for: Three Open Questions in Polygenic Score Portability

Joyce Y. Wang[1], Neeka Lin[1], Michael Zietz[2], Jason Mares[3], Olivia S. Smith[1], Paul J. Rathouz[4,5] and Arbel Harpak[1,5,+]

[1] Department of Integrative Biology, The University of Texas at Austin, Austin, TX

[2] Department of Biomedical Informatics, Columbia University, New York, NY

[3] Department of Neurology, Columbia University, New York, NY

[4] Department of Statistics and Data Science, The University of Texas at Austin, Austin, TX

[5] Department of Population Health, The University of Texas at Austin, Austin, TX

[+] Correspondence should be addressed to A.H. (arbelharpak@utexas.edu)

Contents

1 Sample characteristics and genetic distance **8**

2 The relationship between F_{st} and genotype matrix PCs **9**

3 Sensitivity analyses for major portability trends **9**

List of Tables

1 Characteristics of continuous traits and their PGS. 13

2 Characteristics of binary traits and their PGS. 14

List of Figures

1 Correlation between PC distance and F_{st}. 15

2 Trends of portability vary across traits and measures, for anthropometric measurements. 16

3 Trends of portability vary across traits and measures, for white blood cell-related traits. 17

4 Trends of portability vary across traits and measures, for red blood cell-related traits. 18

5 Trends of portability vary across traits and measures, for other biomarkers. 19

6 Full range of individual level prediction error for anthropometric measurements. 20

7 Full range of individual level prediction error for white blood cell-related traits. 21

8 Full range of individual level prediction error for red blood cell-related traits. 22

9 Full range of individual level prediction error for other biomarkers. 23

10 Divergence of group- and individual-level prediction accuracy for anthropometric measurements. 24
11 Divergence of group- and individual-level prediction accuracy for white blood cell-related traits. 25
12 Divergence of group- and individual-level prediction accuracy for red blood cell-related traits. 26
13 Divergence of group- and individual-level prediction accuracy for other biomarkers. 27
14 Mean trends in individual-level prediction accuracy by different measures for anthropometric measurements. 28
15 Mean trends in individual-level prediction accuracy by different measures for white blood cell-related traits. 29
16 Mean trends in individual-level prediction accuracy by different measures for red blood cell-related traits. 30
17 Mean trends in individual-level prediction accuracy by different measures for other biomarkers. 31
18 Individual-level prediction error explained by different measures for anthropometric measurements. 32
19 Individual-level prediction error explained by different measures for white blood cell-related traits. 33
20 Individual-level prediction error explained by different measures for red blood cell-related traits. 34
21 Individual-level prediction error explained by different measures for other biomarkers. 35
22 Mean heterozygosity of SNPs, stratified by effect size. 36
23 Squared allelic effect estimate of SNPs. 37
24 Heritability explained by index SNPs. 38
25 Mean Townsend Deprivation Index $\pm$ SE in each bin by genetic distance. 39

26 Average total household income before tax in each bin by genetic distance. 40
27 Mean age ± SE in each bin by genetic distance. 41
28 Sex composition across bins of genetic distance. 42
29 Participants' country of residence across bins of genetic distance. 43
30 Correlation between F_{st} and PC 1. 44
31 Correlation between F_{st} and PC 2. 45
32 Correlation between F_{st} and PC 3. 46
33 Correlation between F_{st} and PC 40. 47
34 Trends of portability vary across traits and measures, with confidence intervals, for anthropometric measurements. 48
35 Trends of portability vary across traits and measures, with confidence intervals, for white blood cell-related traits. 49
36 Trends of portability vary across traits and measures, with confidence intervals, for red blood cell-related traits. 50
37 Trends of portability vary across traits and measures, with confidence intervals, for other biomarkers. 51
38 Group level portability, using more bins, for anthropometric measurements. 52
39 Group level portability, using more bins, for white blood cell-related traits. 53
40 Group level portability, using more bins, for red blood cell-related traits. 54
41 Group level portability, using more bins, for other biomarkers. 55
42 Trends of portability, visualized with generalized additive model (GAM) fit, for anthropometric measurements. 56
43 Trends of portability, visualized with generalized additive model (GAM) fit, for white blood cell-related traits. 57
44 Trends of portability, visualized with generalized additive model (GAM) fit, for red blood cell-related traits. 58
45 Trends of portability, visualized with generalized additive model (GAM) fit, for other biomarkers. 59

46 Trends of portability, visualized using 12 knots in cubic line fits, for anthropometric measurements. 60
47 Trends of portability, visualized using 12 knots in cubic line fits, for white blood cell-related traits. 61
48 Trends of portability, visualized using 12 knots in cubic line fits, for red blood cell-related traits. 62
49 Trends of portability, visualized using 12 knots in cubic line fits, for other biomarkers. 63
50 Trends of portability, when including additional GWAS covariates, for anthropometric measurements. 64
51 Trends of portability, when including additional GWAS covariates, for white blood cell-related traits. 65
52 Trends of portability, when including additional GWAS covariates, for red blood cell-related traits. 66
53 Trends of portability, when including additional GWAS covariates, for other biomarkers. 67
54 Trends of portability, with GWAS run using regenie, for anthropometric measurements. 68
55 Trends of portability, with GWAS run using regenie, for white blood cell-related traits. 69
56 Trends of portability, with GWAS run using regenie, for red blood cell-related traits. 70
57 Trends of portability, with GWAS run using regenie, for other biomarkers. 71
58 Trends of portability, with PGS constructed using PRS-CS, for anthropometric measurements. 72
59 Trends of portability, with PGS constructed using PRS-CS, for white blood cell-related traits. 73

60 Trends of portability, with PGS constructed using PRS-CS, for red blood cell-related traits. 74
61 Trends of portability, with PGS constructed using PRS-CS, for other biomarkers. 75
62 A comparison of portability trends, with genetic distance defined in two different ways, for anthropometric measurements. 76
63 A comparison of portability trends, with genetic distance defined in two different ways, for white blood cell-related traits. 77
64 A comparison of portability trends, with genetic distance defined in two different ways, for red blood cell-related traits. 78
65 A comparison of portability trends, with genetic distance defined in two different ways, for other biomarkers. 79
66 Variance in disease PGS as a function of genetic distance. 80
67 Portability, as a function of both genetic distance and Townsend Deprivation Index, for anthropometric measurements. 81
68 Portability, as a function of both genetic distance and Townsend Deprivation Index, for white blood cell-related traits. 82
69 Portability, as a function of both genetic distance and Townsend Deprivation Index, for red blood cell-related traits. 83
70 Portability, as a function of both genetic distance and Townsend Deprivation Index, for other biomarkers. 84
71 Measuring genetic distance from the GWAS sample, with White British individuals included in the prediction set. 85
72 A comparison of portability trends, with different choices of the GWAS and prediction samples, for anthropometric measurements. 86
73 A comparison of portability trends, with different choices of the GWAS and prediction samples, for white blood cell-related traits. 87

74 A comparison of portability trends, with different choices of the GWAS and prediction samples, for red blood cell-related traits. 88
75 A comparison of portability trends, with different choices of the GWAS and prediction samples, for other biomarkers. 89
76 Zoomed-in portability trends, with WB in the prediction set, for anthropometric measurements. 90
77 Zoomed-in portability trends, with WB in the prediction set, for white blood cell-related traits. 91
78 Zoomed-in portability trends, with WB in the prediction set, for red blood cell-related traits. 92
79 Zoomed-in portability trends, with WB in the prediction set, for other biomarkers. 93

1 Sample characteristics and genetic distance

We sought to quantify various sample characteristics that are confounded with genetic distance. We therefore explored the distribution of Townsend Deprivation Index[1] (data field 189), average total household income before tax (data field 738), sex (data field 22001), age (data field 21022), and country of residence (converted from data field 54, into England, Scotland, or Wales) in the GWAS and prediction samples.

For Townsend Deprivation Index[1] (Supplementary Fig. 25) and age (Supplementary Fig. 27), we plotted the mean ± standard error (SE) in each genetic distance bin, along with the values in the GWAS set for reference. For the GWAS sample, we the SE using $\frac{\text{SD}_{\text{GWAS}}}{\sqrt{\overline{n_{\text{pred}}}}}$, where SD_{GWAS} represents the standard deviation (SD) of the measurement in the GWAS set and $\overline{n_{\text{pred}}}$ represents the mean sample size (278 individuals) across all 250 bins in the prediction set. Overall, Townsend Deprivation Index tends to increase (higher deprivarion) and age tends to decrease with genetic distance.

For average total household income before tax, we calculated the frequency of each category in each bin and plotted the cumulative frequency, along with the frequencies in the GWAS set for reference (Supplementary Fig. 26). Household income appears to generally decrease with genetic distance.

For sex, we calculated the frequency of females in each bin and also plotted the mean in the GWAS set. The fraction of females largely decreases up to genetic distances of about 70, but then returns to be high (around 60%) up until genetic distances of about 180 (Supplementary Fig. 28).

For country, we first converted the assessment center to the corresponding country (England, Scotland, or Wales), then calculated the frequency of each category in each bin and plotted the cumulative frequency, along with the frequencies in the GWAS set for reference (Supplementary Fig. 29).

2 The relationship between F_{st} and genotype matrix PCs

Using the same 10,000 randomly selected individuals from the prediction sample (among those with a weighted PC distance greater than the weighted PC distance of 95% of the GWAS set; as described in Methods), we examined the correlation between F_{st} and invidividual coordinates along specific PCs: PCs 1 (Supplementary Fig. 30), 2 (Supplementary Fig. 31), 3 (Supplementary Fig. 32), and 40 (Supplementary Fig. 33). All PCs are taken from UKB field 22009. We color-coded the individuals by self-reported race (UKB field 21000).

3 Sensitivity analyses for major portability trends

We performed several sensitivity analyses to test the sensitivity of our conclusions about portability trends to various choices we made in the analyses presented in the main text.

Binning individuals into 500 bins. To examine the sensitivity of our analyses to binning parameter choices, we perform the analysis with a different binning. We binned individuals in the prediction set into 500 bins of 139 individuals each, instead of 250 bins of 278 individuals each. In Supplementary Figs. 38–41, we show relative prediction accuracy trends using the mean prediction accuracy in the 50 bins with median genetic distances that are closest to the mean genetic distance for GWAS individuals as the baseline value. Comparing the two binning choices, we did not notice any change in the portability trends for any of the quantitative traits analyzed.

Additional GWAS covariates. We examined whether additional GWAS covariates not included in the main text analysis impact our conclusions. We performed polygenic scoring based on new GWAS that included assessment center (UKB data field 54) and genotype array (UKB data field 22000) as additional covariates. We converted these two categorical variables into multiple binary variables using one-hot encoding, and re-

moved one column from either of the variables to prevent multicollinearity. We followed the same procedures as described in the Methods from the main text for clumping and thresholding, PGS construction and analysis of prediction accuracy trends at the group and individual levels for 15 quantitative traits (Supplementary Figs. 50–53). Adding these covariates did not lead to any prominent change in the portability trends for most traits; for lymphocyte and monocyte counts, the addition of these covariates generally resulted in better prediction at large genetic distances.

Performing GWAS with regenie. To test the sensitivity of our three conclusions to the GWAS methods we used, we re-ran GWAS for all 15 quantitative traits we analyzed using the same covariates as described in **Methods**, but using regenie[2]. Then we followed the same procedures for clumping and thresholding with PLINK, as described in Methods from the main text. After that, we computed the PGS prediction accuracy at the group and individual levels (Supplementary Figs. 54–57). We saw no major change in the portability trends for most traits. For lymphocyte count, however, prediction accuracy at the group level was noticeably higher in some bins.

Constructing PGS with PRS-CS. To examine the sensitivity of our conclusions to the choice of method for PGS construction, we used an approach distinct from clumping and thresholding—PRS-CS[3], running with Python[4] and using the `--score` flag. PRS-CS applies Bayesian shrinkage to GWAS-based allelic effect estimates. It also aims to consider LD patterns more rigorously and holistically, rather than via the clumping and thresholding heuristic. To run PRS-CS, we used the same GWAS summary statistics as before and the LD reference panel constructed using the UKB EUR super-population data[3]. For the optional gamma-gamma prior parameters a, b, we used the default values, which are 1 and 0.5, respectively. The global shrinkage parameter ϕ was set to the automatically estimated value provided by PRS-CS. We computed the PGS prediction accuracy at the group and individual levels for 15 quantitative traits (Supplementary Figs. 58–61). Using PRS-CS, the peak at intermediate genetic distances for weight and BMI at the group level became less prominent. For lymphocyte count and white blood

cell count, the decrease in prediction at the group level became more gradual. However, the general trends of portability of other traits did not change.

Defining genetic distance using 16 instead of 40 PCs. Previous research puts into question the optimal number of genotype matrix PCs that should be considered, both generally in genetic analyses and for specific tasks[5,6,7]. In the main text, we use a genetic distance metric of an eigenvalue-weighted Euclidean distance in PCA space. The PCA is the pre-computed PCA of the genotype matrix of the UK Biobank sample, and the genetic distance from the GWAS sample is the distance of a given individual's PC coordinates from the centroid of GWAS sample individuals (Methods). Here, we repeated this calculation using the first 16 PCs provided by UKB (UKB data field 22009) rather than the first 40. Among all samples, the Pearson's and Spearman's correlations between genetic distances calculated from 40 PCs and from 16 PCs are both greater than 0.99. We computed PGS prediction accuracy at the group and individual levels for 15 quantitative traits as before. Across all 15 quantitative traits, there were no noticeable changes in portability trends (Supplementary Figs. 62–65).

Continuous trend visualization. To examine whether our conclusions are sensitive to the continuous function fits we used for visualizations, we first examined the effect of a change in the cubic spline parameters. Namely, instead of 8 knots fitted with `bs()` from the R library `splines`[8], we used 12 (Supplementary Figs. 46–49). Using 12 knots, the fit appears more flexible, but the general trends of portability discussed in the main text did not change.

We also tested a different functional form for the fit, a generalized additive model (GAM). We used `gam()` from the R library mgcv[9] instead of a cubic spline (Supplementary Figs. 42–45). For comparison, we placed the 8 knots at the same positions for the GAM model. The GAM fit appears to be smoother at the group level for most traits, but have the same general trends as the cubic spline fit.

Portability trends stratified by Townsend Deprivation Index. In the main text analysis, we discuss the observation that, across traits, genetic distance and Townsend

Deprivation Index[1] explain comparable fractions of variation in individual-level prediction accuracy (Fig. 3B). Here, we examine the two predictors' joint effect. In particular, we stratify individuals by Townsend Deprivation Index and visualize trends of prediction accuracy as a function of genetic distance in each SES stratum independently.

We performed this analysis at both the group and individual levels. At the group level, we stratified the prediction individuals into 2 equal-size strata of Townsend Deprivation Index. At the individual level, we binned individuals into 5 equal-sized strata. In each stratum independently, we then followed the analysis as otherwise described in Methods from the main text to calculate prediction accuracy at the group and individual levels. The results are shown in Supplementary Figs. 67–70.

Including White British individuals in the prediction set. In the main text, we opted to include all White British individuals in the GWAS sample and none in the prediction set. Here, we visit the sensitivity of our conclusions to this choice by reducing the GWAS sample size to 300,000 randomly selected White British individuals and adding the remaining 36,923 White British individuals to the prediction sample (now totaling 106,423 individuals). We otherwise followed the same procedures as described in Methods from the main text, verifying that, across 10,000 individuals from the prediction set, genetic distance (calculated with 40 pre-computed PCs provided in UKB data field 22009) remained highly correlated with F_{st} between the new GWAS sample and the individual. The distribution of genetic distance with this new choice of GWAS sample and prediction sample is shown in Supplementary Fig. 71.

We measure the prediction accuracy at the group and individual levels using the same procedures as described in Methods from the main text. Since the prediction set became larger, we binned the individuals into 382 bins of 279 individuals each at the group level (compared to 250 bins of 278 individuals each in the main text analysis). Across traits, we observed similar qualitative trends as we did in the main text analysis (Supplementary Figs. 72–75). Notably, in short genetic distances (< 5.2) the group-level prediction accuracy tended to be near-constant (Supplementary Figs. 76–79).

Phenotype	SNP h^2	Mean squared prediction error (MSE)	Variance of residualized phenotype (var$[Z]$)	$1 - \frac{\textbf{MSE}}{\textbf{var}[Z]}$	Prediction accuracy (partial R^2)
Height	0.4852	31.8859	41.5853	0.2332	0.2473
Cystatin C level	0.3214	0.0319	0.0333	0.0426	0.0632
Platelet count	0.3079	2950.5930	3369.6670	0.1244	0.1365
Mean corpuscular volume	0.2667	16.5441	18.7806	0.1191	0.1303
Weight	0.2654	205.3049	217.1554	0.0546	0.0620
Mean corpuscular hemoglobin	0.2530	2.6206	2.9492	0.1114	0.1253
BMI	0.2482	23.7059	24.6588	0.0386	0.0442
Red blood cell count	0.2337	0.1061	0.1161	0.0859	0.0912
Body fat percentage	0.0472	41.7102	43.2307	0.0352	0.0419
Monocyte count	0.2305	0.03674	0.0380	0.0325	0.0870
Triglyceride level	0.2182	0.9468	1.0010	0.0542	0.0620
Lymphocyte count	0.2103	1.1740	1.1753	0.0011	0.0342
White blood cell count	0.1910	3.8944	4.0020	0.0269	0.0455
Eosinophil count	0.1840	0.0195	0.0204	0.0430	0.0505
LDL cholesterol level	0.0825	0.6270	0.6810	0.0792	0.0887

Supplementary Table 1: Characteristics of continuous traits and PGS analyzed. We binned the prediction individuals into 250 bins of 278 individuals each by genetic distance. SNP heritabilities (SNP h^2) are taken from the Neale Lab's UKB analysis[10]. For the 25 bins with a genetic distance most similar to the mean genetic distance of the GWAS group, we calculated the group-level prediction accuracy (partial genetic correlation of the PGS and the trait value), mean individual prediction error (squared prediction error), the variance of residualized phenotype, and the ratio between the two subtracted from one, as another measure of phenotype variance explained by the PGS close to the GWAS sample. No repeated measurements from the same individual were taken.

Phenotype	Number of ref. bins	Mean precision	Mean recall
Asthma	25	0.1076	0.3481
Type 2 diabetes	25	0.0944	0.3302

Supplementary Table 2: Characteristics of binary traits and PGS analyzed. We binned the prediction individuals into 250 bins of 278 individuals each by genetic distance. For the reference bins (bins with a genetic distance most similar to the mean genetic distance of the GWAS group), we provide the mean precision and recall values in this table. No repeated measurements from the same individual were taken.

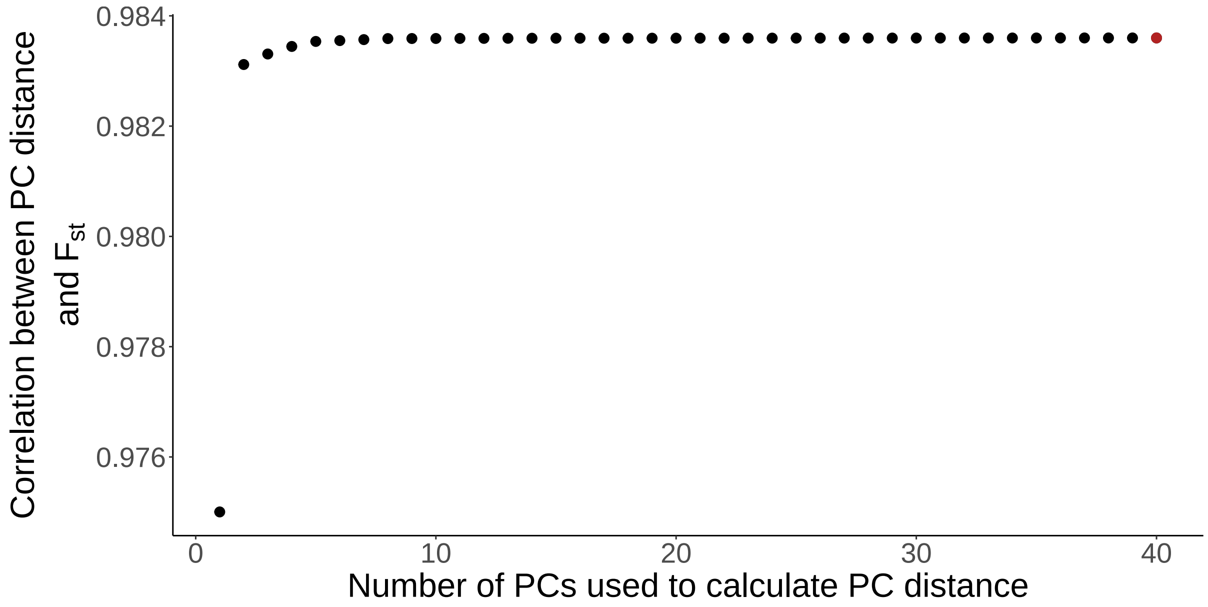

Supplementary Figure 1: Correlation between PC distance and F_{st}. Shown are the Pearson's correlations between PC distance using a given number of UKB PCs and F_{st} between the GWAS sample and individuals. The correlations are calculated using a subset of 10,000 randomly selected prediction sample individuals, among those with a weighted PC distance greater than the weighted PC distance of 95% of the GWAS set (based on weighted PC distance calculated with $K = 10$), as described in Methods from the main text. Using 40 PCs produces the highest correlation between PC distance and F_{st} (red dot, Pearson's $r > 0.9835$, p-value $< 2.2 \times 10^{-16}$). No repeated measurements from the same individual were taken. For correlation between F_{st} and PCs 1, 2, 3, and 40, see Supplementary Figs. 30–33.

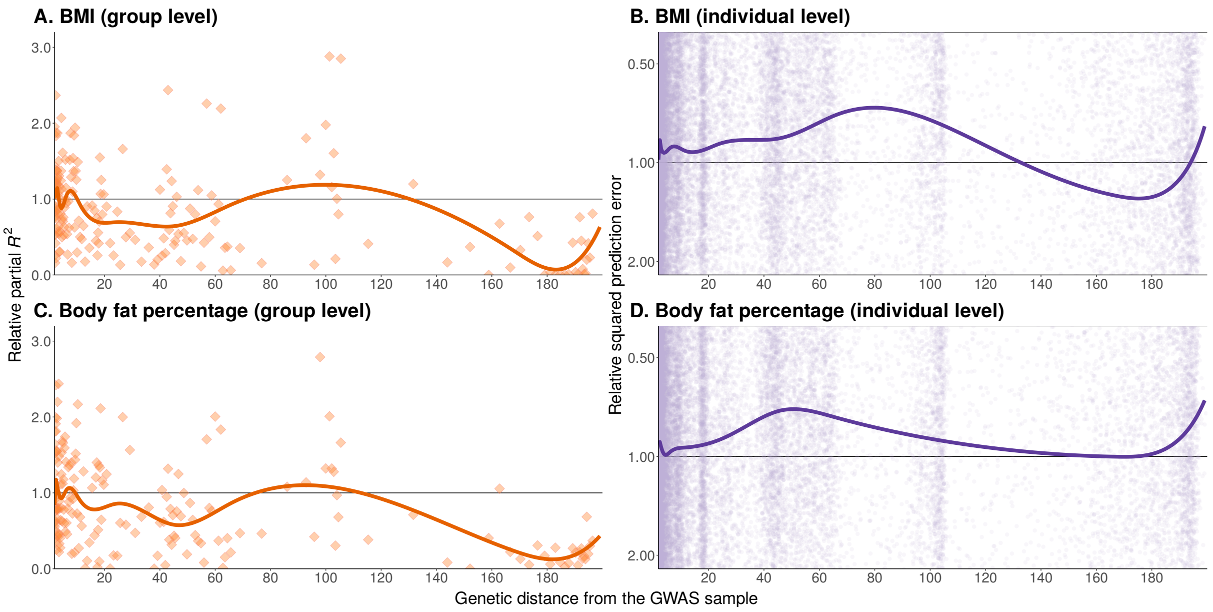

Supplementary Figure 2: Trends of portability vary across traits and measures, for anthropometric measurements. This figure presents the same analysis as Fig. 2 in the main text, but shows other anthropometric traits. At the group level (A, C), we measured prediction accuracy with the squared partial correlation between the PGS and the trait value in 250 bins of 278 individuals each. At the individual level (B, D), we measured prediction accuracy as the squared difference between the predicted phenotype and the true phenotype value. At both the group and individual levels, y-axis values show relative prediction accuracy, i.e. prediction accuracy divided by a baseline value. The baseline value is the mean prediction accuracy in 25 bins with median genetic distances that are closest to the mean genetic distance for GWAS individuals. Supplementary Table 1 details these baseline values for each trait. Curves show cubic spline fits, with 8 knots placed based on the density of data points. See Supplementary Fig. 6–9 for plots showing the full ranges of individual-level prediction accuracy. For all panels, no repeated measurements from the same individual were taken.

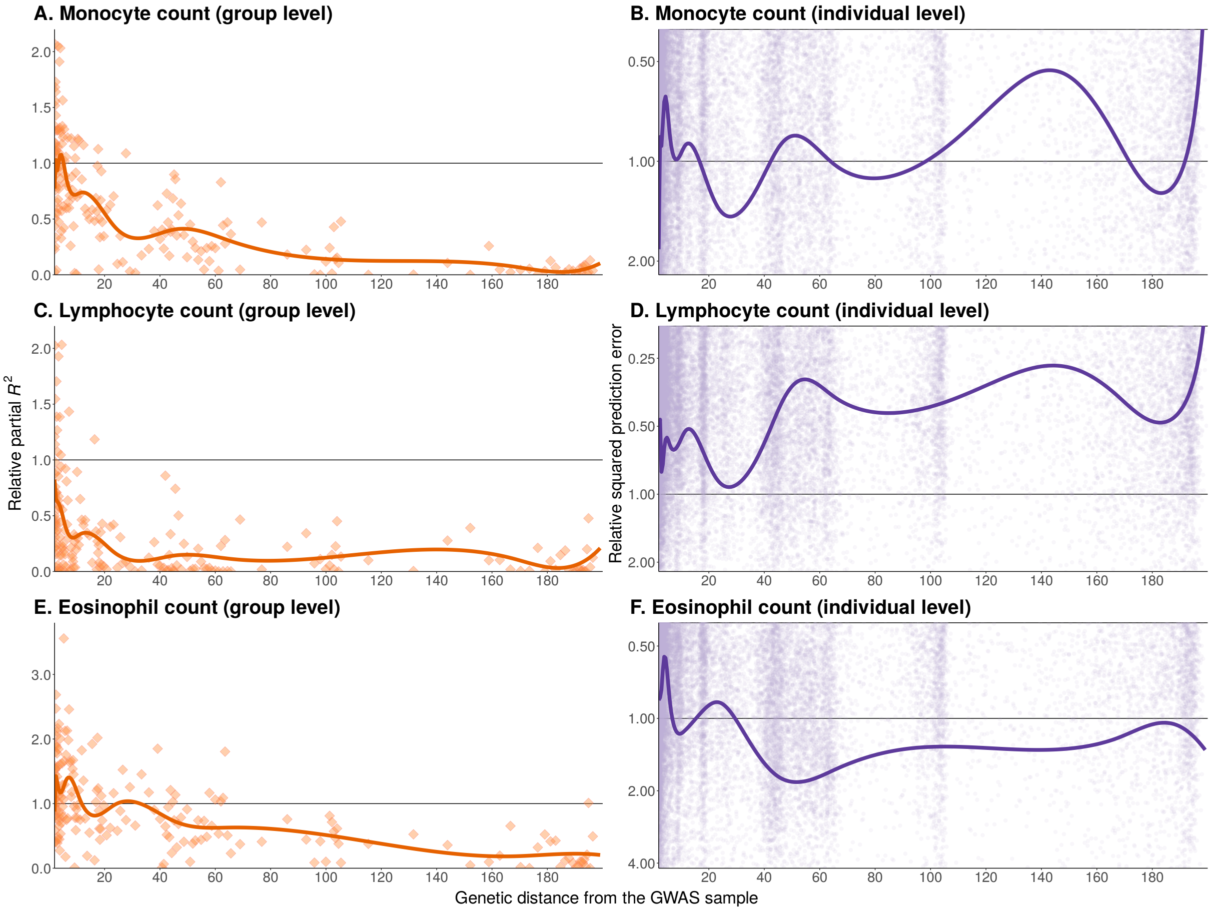

Supplementary Figure 3: Trends of portability vary across traits and measures, for white blood cell-related traits. This figure presents the same analysis as Fig. 2 in the main text, but shows other white blood cell-related traits. At the group level (A, C, E), we measured prediction accuracy with the squared partial correlation between the PGS and the trait value in 250 bins of 278 individuals each. At the individual level (B, D, F), we measured prediction accuracy as the squared difference between the predicted phenotype and the true phenotype value. At both the group and individual levels, y-axis values show relative prediction accuracy, i.e. prediction accuracy divided by a baseline value. The baseline value is the mean prediction accuracy in 25 bins with median genetic distances that are closest to the mean genetic distance for GWAS individuals. Supplementary Table 1 details these baseline values for each trait. Curves show cubic spline fits, with 8 knots placed based on the density of data points. See Supplementary Figs. 6–9 for plots showing the full ranges of individual-level prediction accuracy. For all panels, no repeated measurements from the same individual were taken.

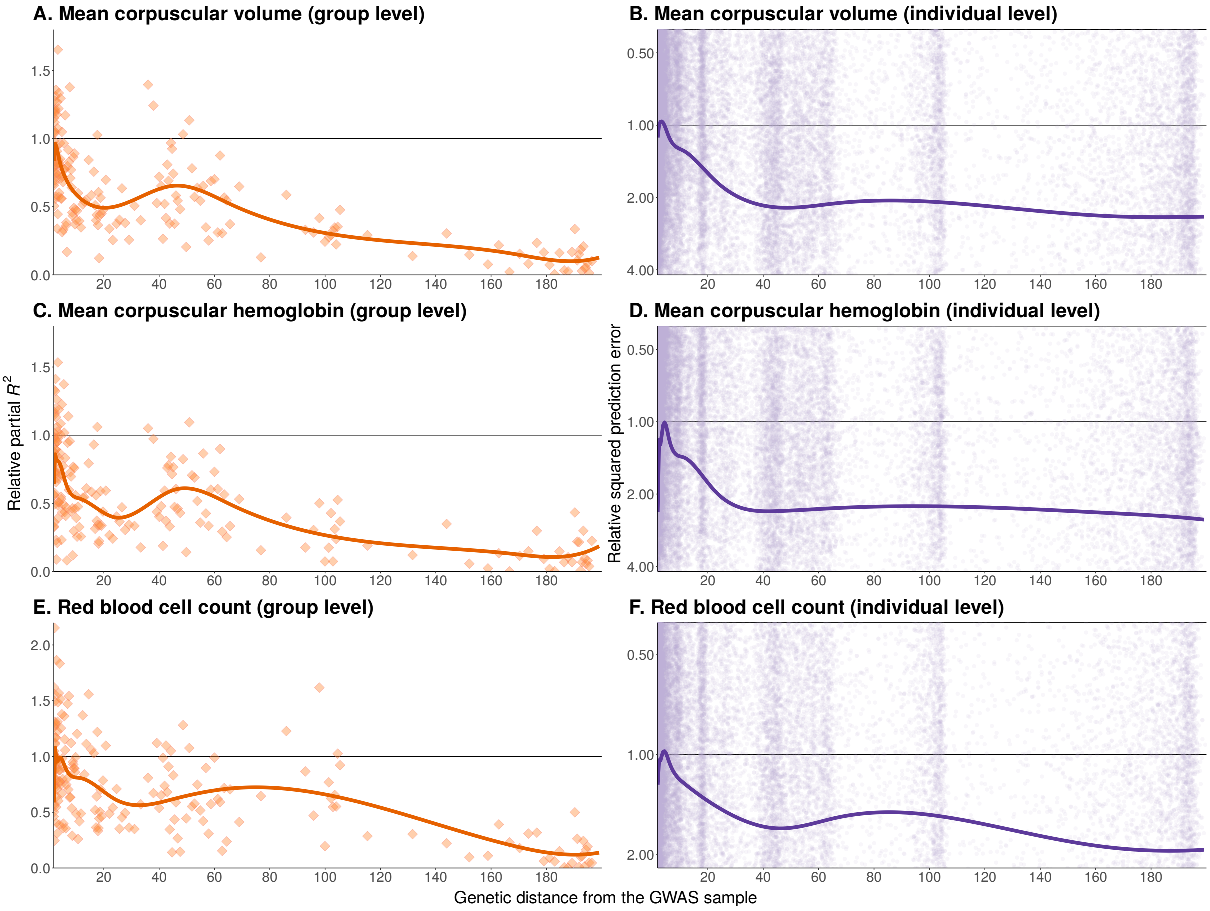

Supplementary Figure 4: Trends of portability vary across traits and measures, for red blood-cell related traits. This figure presents the same analysis as Fig. 2 in the main text, but shows other red blood cell-related traits. At the group level (A, C, E), we measured prediction accuracy with the squared partial correlation between the PGS and the trait value in 250 bins of 278 individuals each. At the individual level (B, D, F), we measured prediction accuracy as the squared difference between the predicted phenotype and the true phenotype value. At both the group and individual levels, y-axis values show relative prediction accuracy, i.e. prediction accuracy divided by a baseline value. The baseline value is the mean prediction accuracy in 25 bins with median genetic distances that are closest to the mean genetic distance for GWAS individuals. Supplementary Table 1 details these baseline values for each trait. Curves show cubic spline fits, with 8 knots placed based on the density of data points. See Supplementary Figs. 6–9 for plots showing the full ranges of individual-level prediction accuracy. For all panels, no repeated measurements from the same individual were taken.

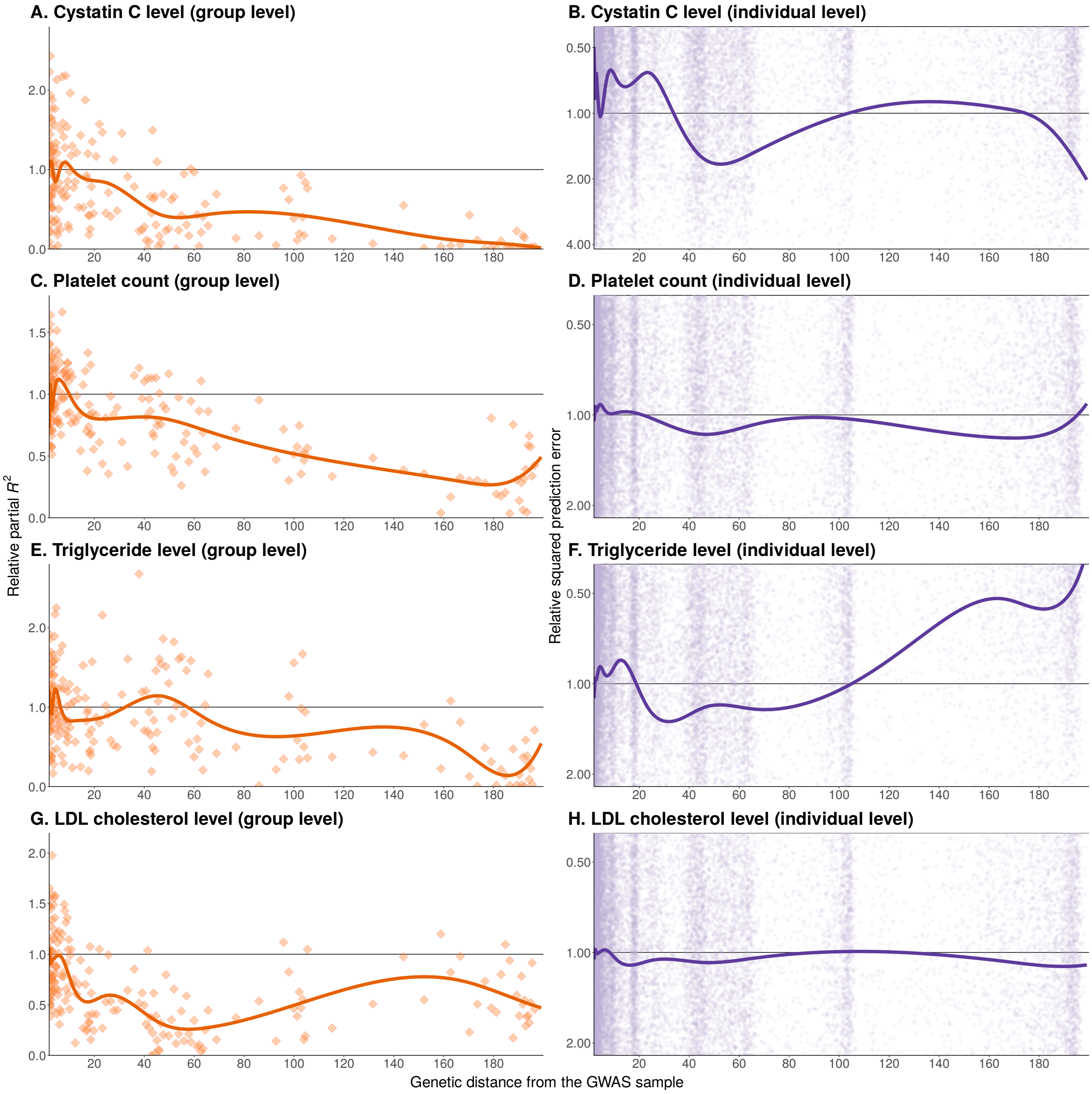

Supplementary Figure 5: Trends of portability vary across traits and measures, for other biomarkers. This figure presents the same analysis as Fig. 2 in the main text, but shows other biomarkers. At the group level (A, C, E), we measured prediction accuracy with the squared partial correlation between the PGS and the trait value in 250 bins of 278 individuals each. At the individual level (B, D, F), we measured prediction accuracy as the squared difference between the predicted phenotype and the true phenotype value. At both the group and individual levels, y-axis values show relative prediction accuracy, i.e. prediction accuracy divided by a baseline value. The baseline value is the mean prediction accuracy in 25 bins with median genetic distances that are closest to the mean genetic distance for GWAS individuals. Supplementary Table 1 details these baseline values for each trait. Curves show cubic spline fits, with 8 knots placed based on the density of data points. See Supplementary Figs. 6–9 for plots showing the full ranges of individual-level prediction accuracy. For all panels, no repeated measurements from the same individual were taken.

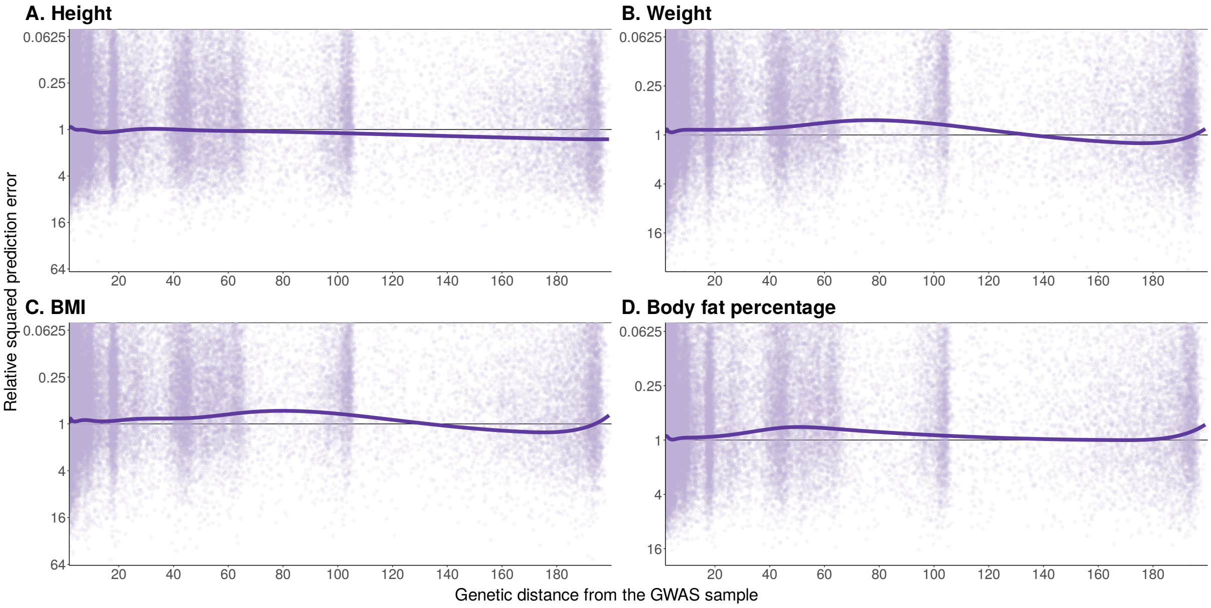

Supplementary Figure 6: Full range of individual level prediction error for anthropometric measurements. This figure presents the same analysis as Fig. 2 in the main text, but shows the full range of prediction error for other anthropometric traits. At the individual level, we measured prediction accuracy as the squared difference between the predicted phenotype and the true phenotype value for 69,500 prediction samples. The y-axis values show relative prediction accuracy, i.e. prediction accuracy divided by a baseline value. The baseline value is the mean prediction accuracy in 25 bins with median genetic distances that are closest to the mean genetic distance for GWAS individuals. Supplementary Table 1 details these baseline values for each trait. Curves show cubic spline fits, with 8 knots placed based on the density of data points. See Supplementary Figs. 2–5 for plots showing the zoomed-in version of individual-level prediction accuracy. For all panels, no repeated measurements from the same individual were taken.

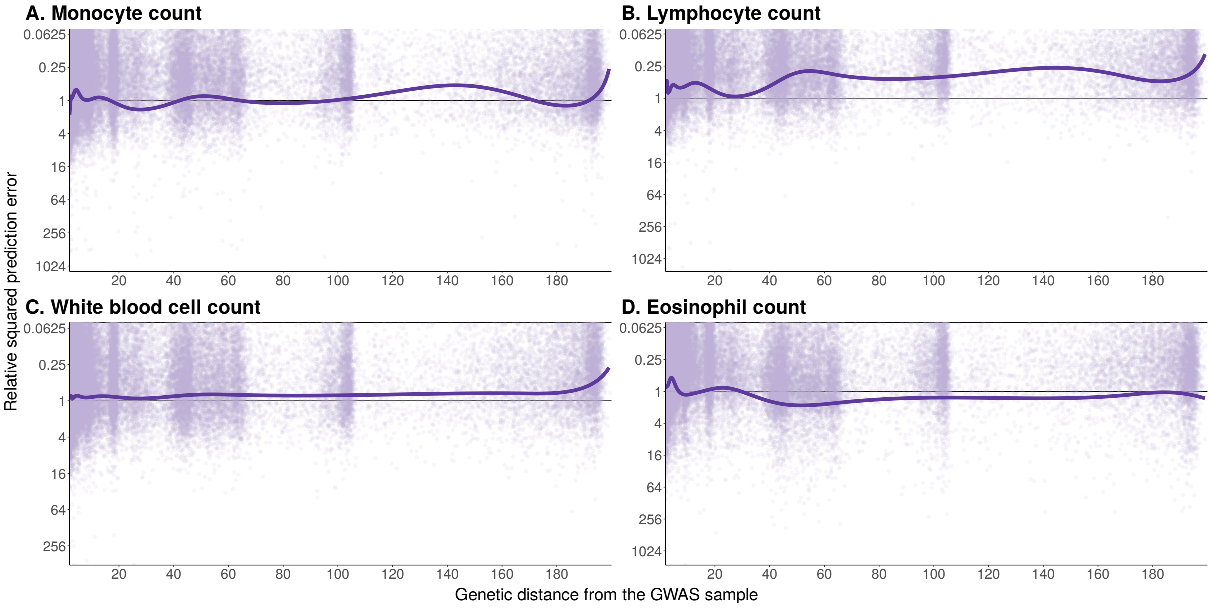

Supplementary Figure 7: Full range of individual level prediction error for white blood cell-related traits. This figure presents the same analysis as Fig. 2 in the main text, but shows the full range of prediction error for white blood cell-related traits. At the individual level, we measured prediction accuracy as the squared difference between the predicted phenotype and the true phenotype value for 69,500 prediction samples. The y-axis values show relative prediction accuracy, i.e. prediction accuracy divided by a baseline value. The baseline value is the mean prediction accuracy in 25 bins with median genetic distances that are closest to the mean genetic distance for GWAS individuals. Supplementary Table 1 details these baseline values for each trait. Curves show cubic spline fits, with 8 knots placed based on the density of data points. See Supplementary Figs. 2–5 for plots showing the zoomed-in version of individual-level prediction accuracy. For all panels, no repeated measurements from the same individual were taken.

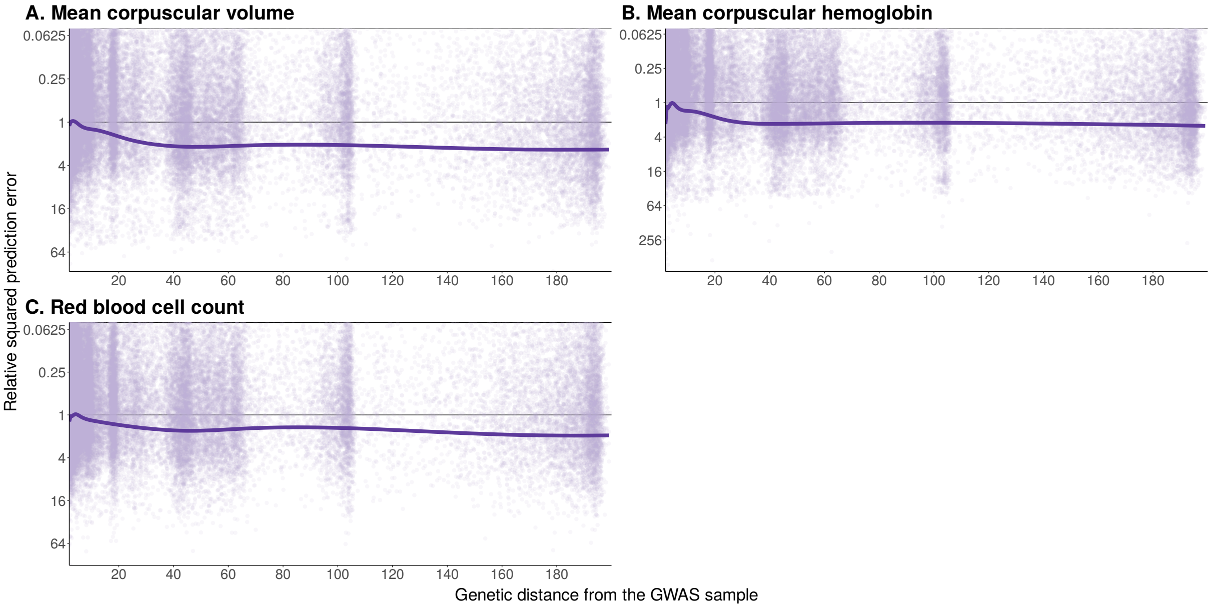

Supplementary Figure 8: Full range of individual level prediction error for red blood cell-related traits. This figure presents the same analysis as Fig. 2 in the main text, but shows the full range of prediction error for red blood cell-related traits. At the individual level, we measured prediction accuracy as the squared difference between the predicted phenotype and the true phenotype value for 69,500 prediction samples. The y-axis values show relative prediction accuracy, i.e. prediction accuracy divided by a baseline value. The baseline value is the mean prediction accuracy in 25 bins with median genetic distances that are closest to the mean genetic distance for GWAS individuals. Supplementary Table 1 details these baseline values for each trait. Curves show cubic spline fits, with 8 knots placed based on the density of data points. See Supplementary Figs. 2–5 for plots showing the zoomed-in version of individual-level prediction accuracy. For all panels, no repeated measurements from the same individual were taken.

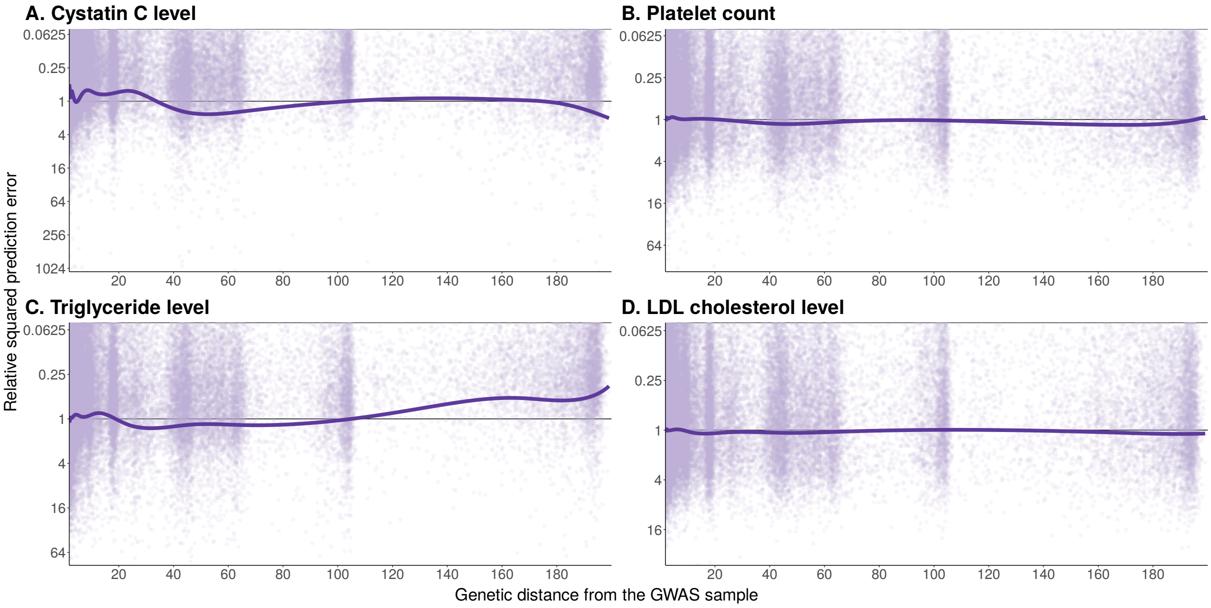

Supplementary Figure 9: Full range of individual level prediction error for other biomarkers. This figure presents the same analysis as Fig. 2 in the main text, but shows the full range of prediction error for other biomarkers. At the individual level, we measured prediction accuracy as the squared difference between the predicted phenotype and the true phenotype value for 69,500 prediction samples. The y-axis values show relative prediction accuracy, i.e. prediction accuracy divided by a baseline value. The baseline value is the mean prediction accuracy in 25 bins with median genetic distances that are closest to the mean genetic distance for GWAS individuals. Supplementary Table 1 details these baseline values for each trait. Curves show cubic spline fits, with 8 knots placed based on the density of data points. See Supplementary Figs. 2–5 for plots showing the zoomed-in version of individual-level prediction accuracy. For all panels, no repeated measurements from the same individual were taken.

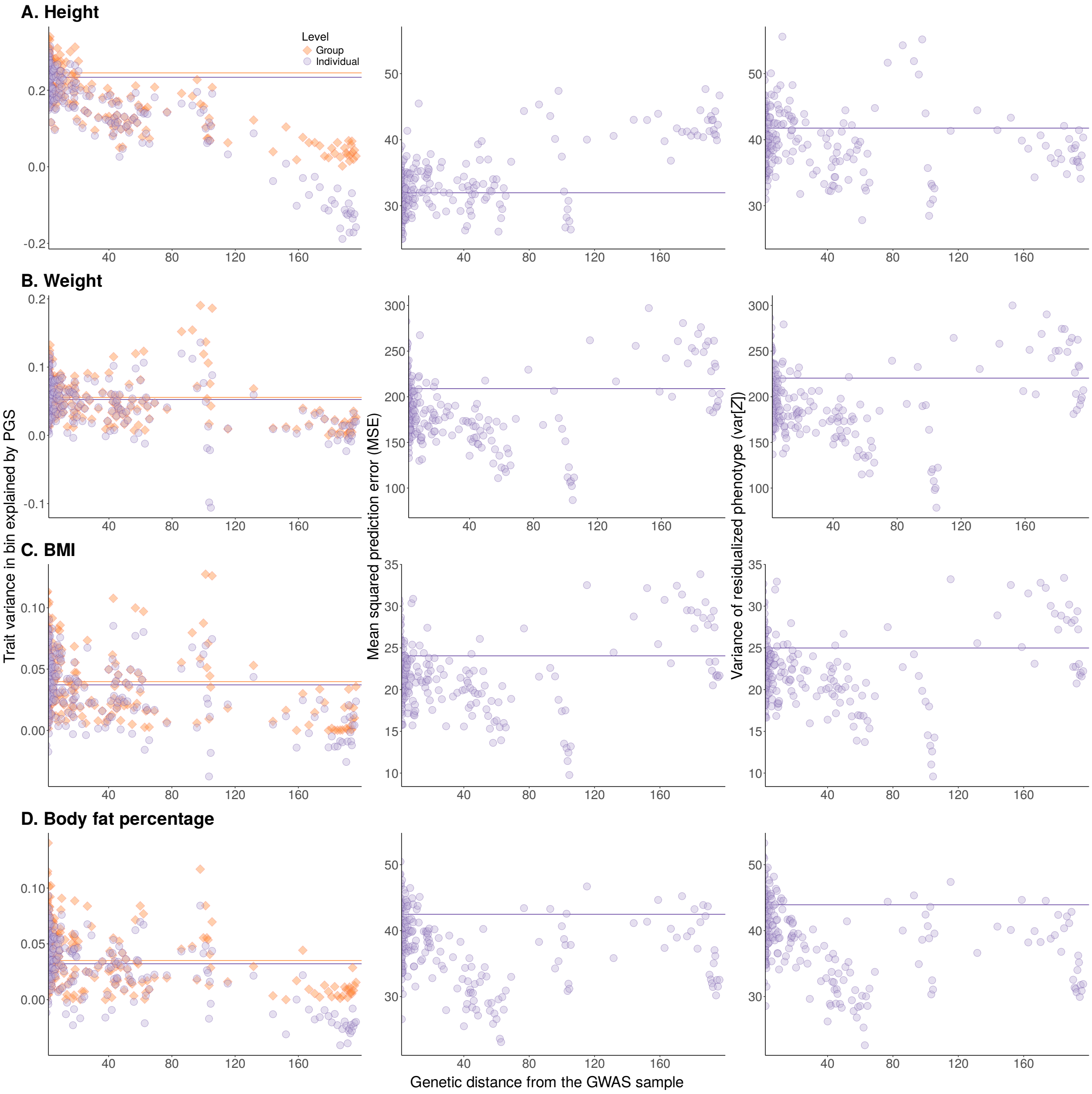

Supplementary Figure 10: Divergence of group- and individual-level prediction accuracy for anthropometric measurements. We showed the results for 250 bins of 278 prediction individuals each, binned by genetic distance. The squared prediction error is with respect to the PGS as a predictor of a phenotypic value residualized for covariates (Z). Its mean (MSE) and the variation of residualized phenotype (var$[Z]$) in each bin are shown in the middle and right columns, respectively. In the left column, we show measures of the variance explained in each bin. Group level refers to the unstandardized partial R^2 between PGS and phenotype. Individual level refers to $1 - \frac{\text{MSE}}{\text{var}[Z]}$. Horizontal lines show mean values across the 25 bins with a genetic distance closest to the mean genetic distance of the GWAS sample individuals (reference bins). For all panels, no repeated measurements from the same individual were taken. The values of the 25 reference bins in all panels can be found in Supplementary Table 1.

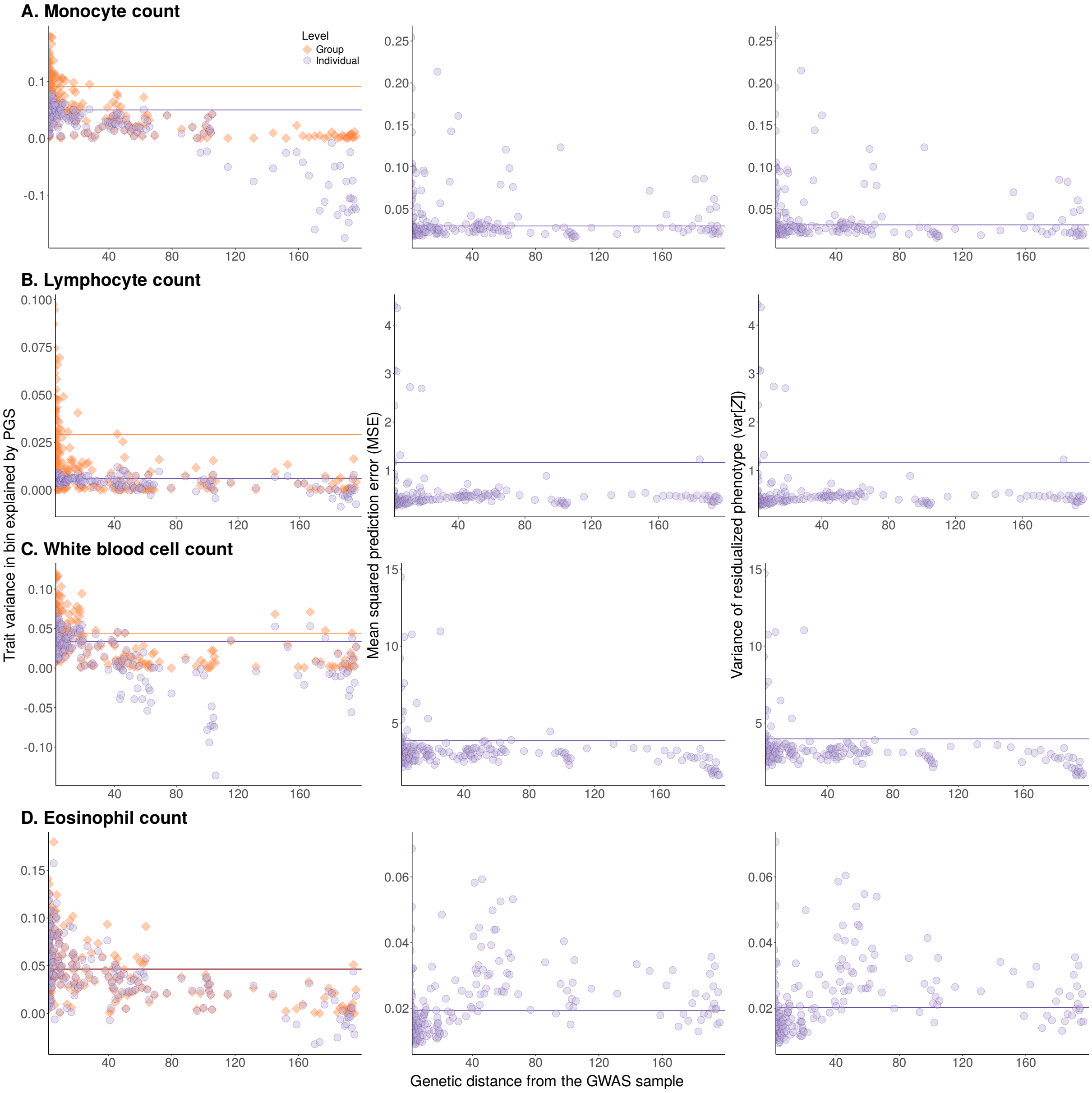

Supplementary Figure 11: Divergence of group- and individual-level prediction accuracy for white blood cell-related traits. We showed the results for 250 bins of 278 prediction individuals each, binned by genetic distance. The squared prediction error is with respect to the PGS as a predictor of a phenotypic value residualized for covariates (Z). Its mean (MSE) and the variation of residualized phenotype (var$[Z]$) in each bin are shown in the middle and right columns, respectively. In the left column, we show measures of the variance explained in each bin. Group level refers to the unstandardized partial R^2 between PGS and phenotype. Individual level refers to $1 - \frac{\text{MSE}}{\text{var}[Z]}$. Horizontal lines show mean values across the 25 bins with a genetic distance closest to the mean genetic distance of the GWAS sample individuals (reference bins). For all panels, no repeated measurements from the same individual were taken. The values of the 25 reference bins in all panels can be found in Supplementary Table 1.

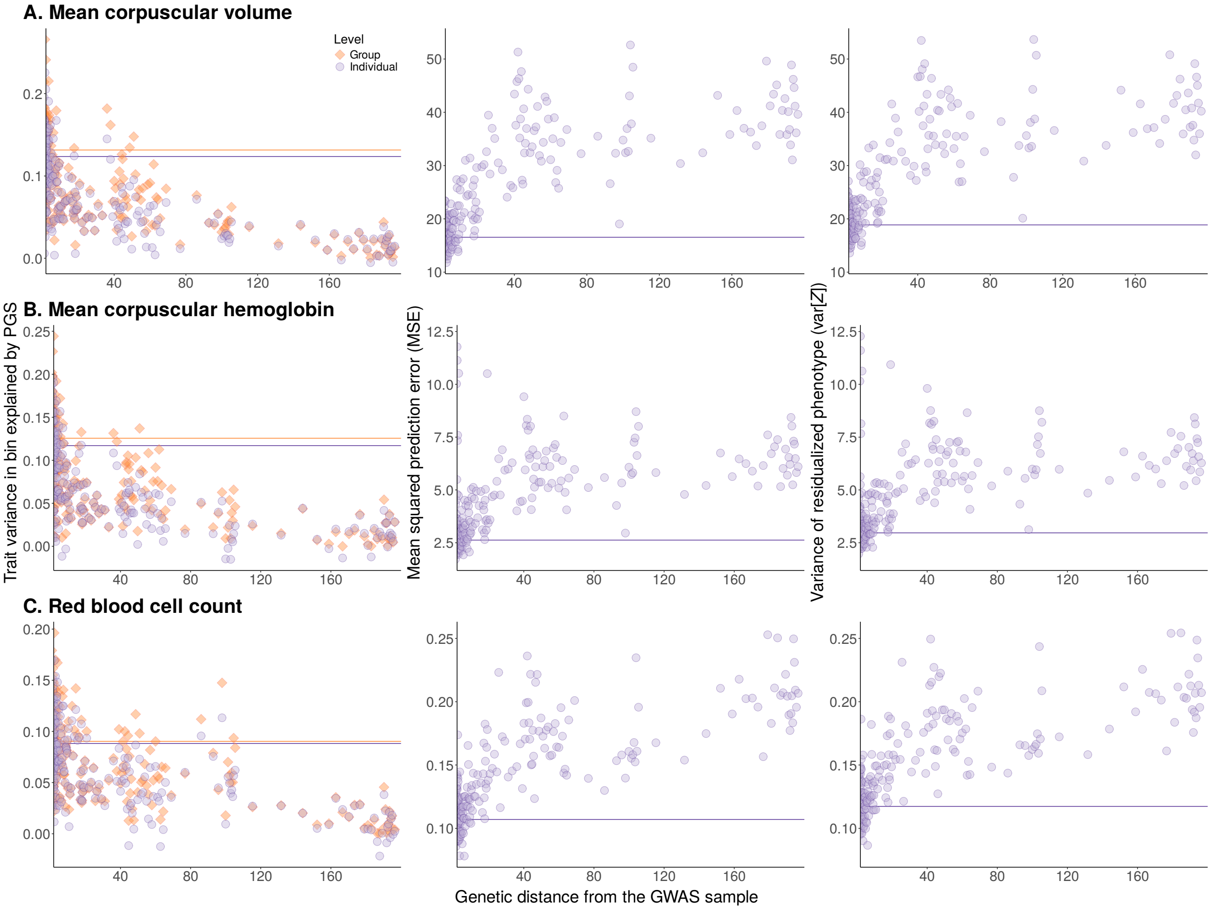

Supplementary Figure 12: Divergence of group- and individual-level prediction accuracy for red blood cell-related traits. We showed the results for 250 bins of 278 prediction individuals each, binned by genetic distance. The squared prediction error is with respect to the PGS as a predictor of a phenotypic value residualized for covariates (Z). Its mean (MSE) and the variation of residualized phenotype (var$[Z]$) in each bin are shown in the middle and right columns, respectively. In the left column, we show measures of the variance explained in each bin. Group level refers to the unstandardized partial R^2 between PGS and phenotype. Individual level refers to $1 - \frac{\text{MSE}}{\text{var}[Z]}$. Horizontal lines show mean values across the 25 bins with a genetic distance closest to the mean genetic distance of the GWAS sample individuals (reference bins). For all panels, no repeated measurements from the same individual were taken. The values of the 25 reference bins in all panels can be found in Supplementary Table 1.

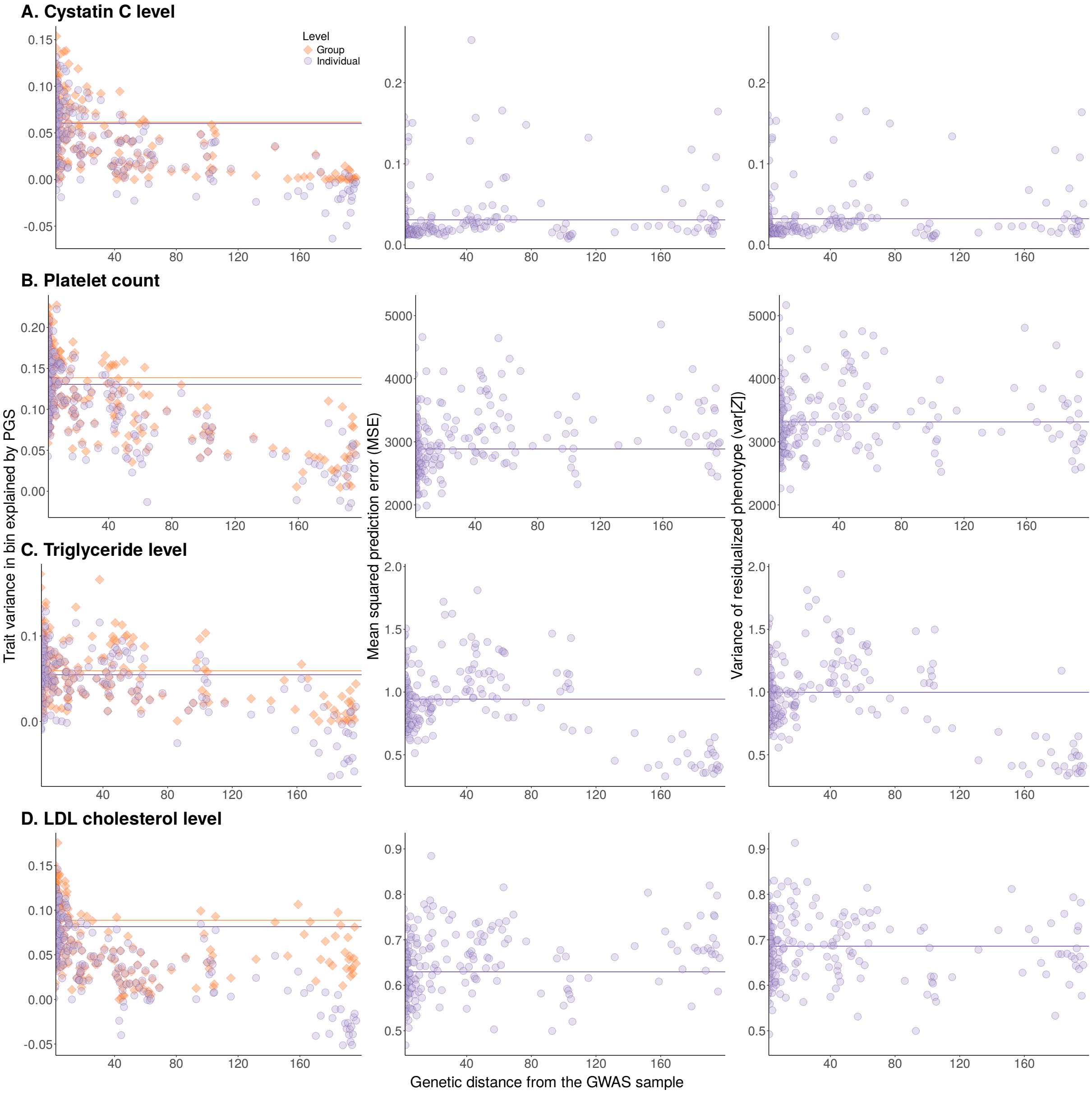

Supplementary Figure 13: Divergence of group- and individual-level prediction accuracy for other biomarkers. We showed the results for 250 bins of 278 prediction individuals each, binned by genetic distance. The squared prediction error is with respect to the PGS as a predictor of a phenotypic value residualized for covariates (Z). Its mean (MSE) and the variation of residualized phenotype (var$[Z]$) in each bin are shown in the middle and right columns, respectively. In the left column, we show measures of the variance explained in each bin. Group level refers to the unstandardized partial R^2 between PGS and phenotype. Individual level refers to $1 - \frac{\text{MSE}}{\text{var}[Z]}$. Horizontal lines show mean values across the 25 bins with a genetic distance closest to the mean genetic distance of the GWAS sample individuals (reference bins). For all panels, no repeated measurements from the same individual were taken. The values of the 25 reference bins in all panels can be found in Supplementary Table 1.

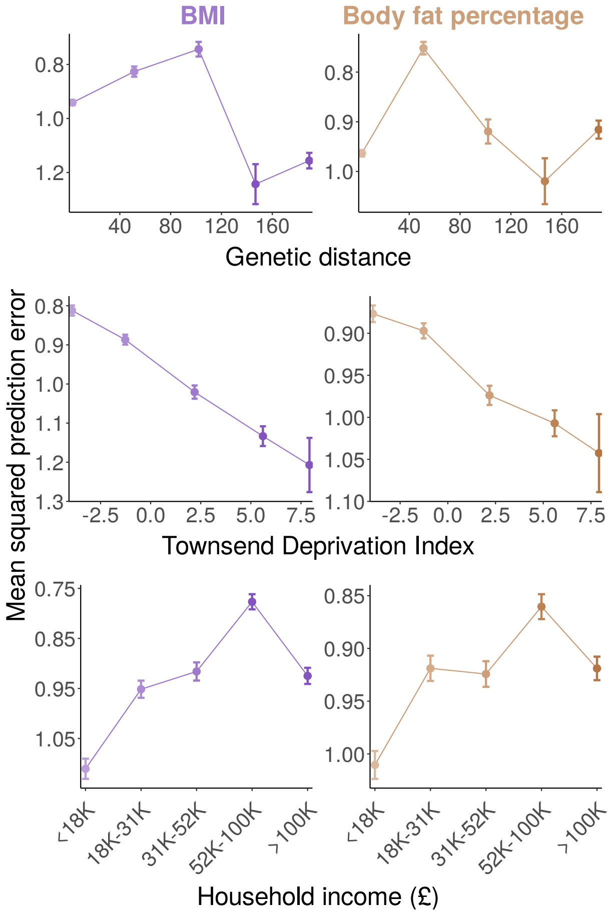

Supplementary Figure 14: Mean trends in individual-level prediction accuracy by different measures for anthropometric measurements. This figure presents the same analysis as Fig. 3A in the main text, but shows other anthropometric traits: body mass index and body fat percentage. The y-axis corresponds to the mean (±SE) squared prediction errors of 69,500 individuals in the prediction set. For genetic distance and Townsend Deprivation Index, individuals are binned into 5 equidistant strata, and the x-axis shows the median measure value for each stratum. Household income refers to the average yearly total household income before tax, and the 5 strata are taken directly from UKB data field 738. No repeated measurements from the same individual were taken.

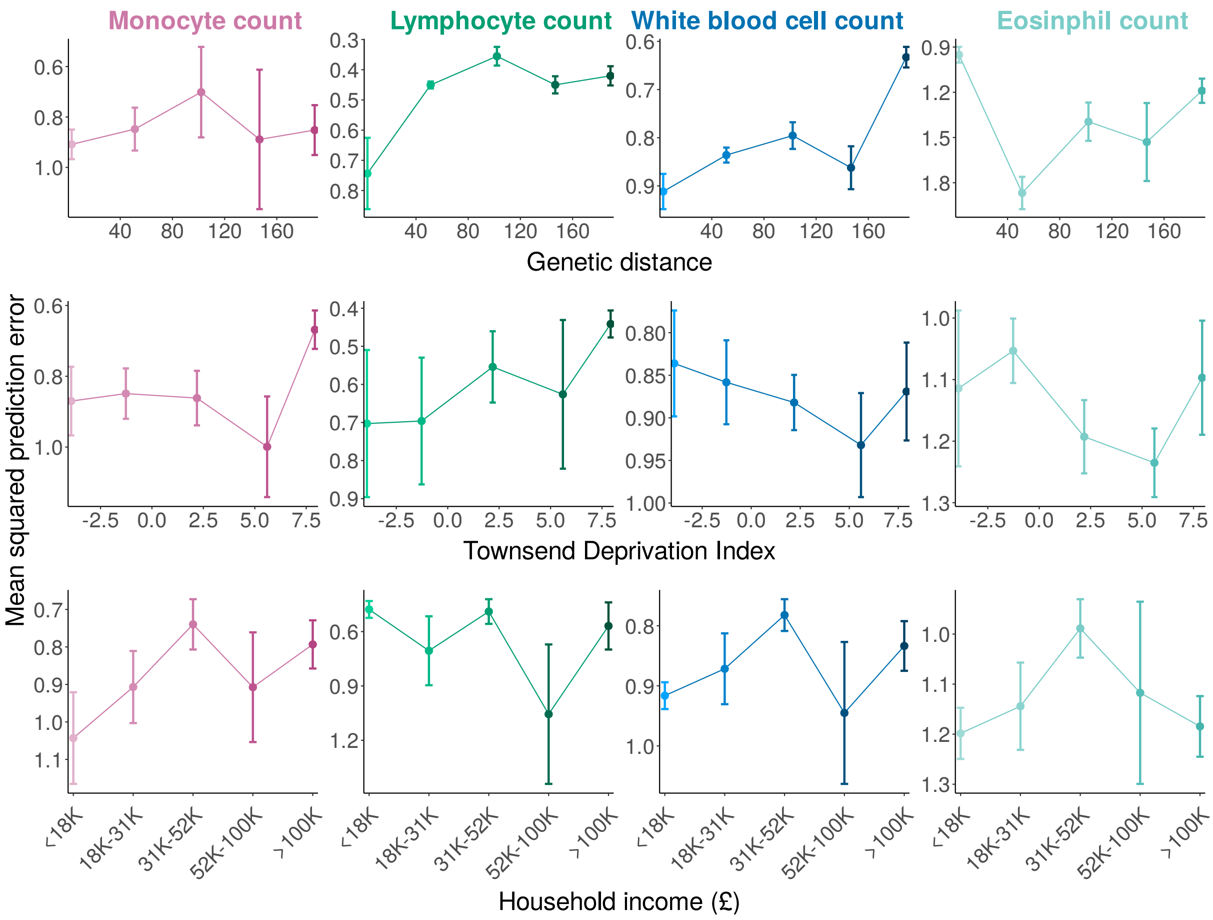

Supplementary Figure 15: Mean trends in individual-level prediction accuracy by different measures for white blood cell-related traits. This figure presents the same analysis as Fig. 3A in the main text, but shows the mean trends for white blood cell-related traits. The y-axis corresponds to the mean (±SE) squared prediction errors of 69,500 individuals in the prediction set. For genetic distance and Townsend Deprivation Index, individuals are binned into 5 equidistant strata, and the x-axis shows the median measure value for each stratum. Household income refers to the average yearly total household income before tax, and the 5 strata are taken directly from UKB data field 738. No repeated measurements from the same individual were taken.

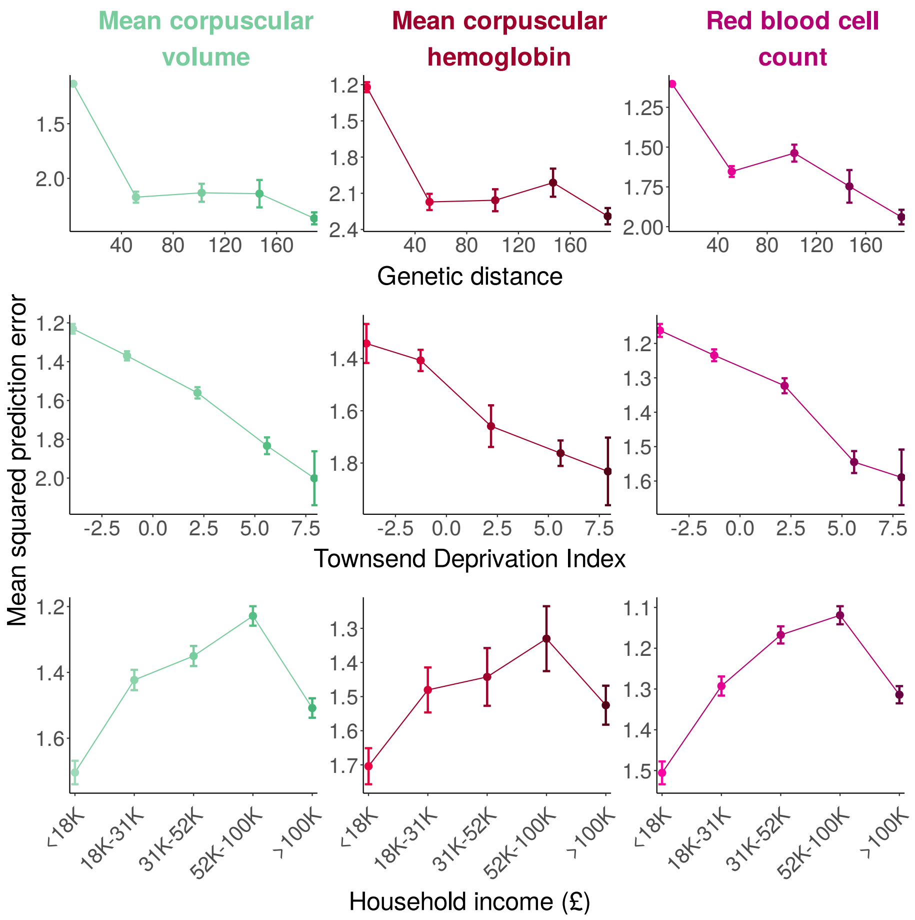

Supplementary Figure 16: Mean trends in individual-level prediction accuracy by different measures for red blood cell-related traits. This figure presents the same analysis as Fig. 3A in the main text, but shows different traits: mean corpuscular volume, mean corpuscular hemoglobin, and red blood cell count. The y-axis corresponds to the mean (±SE) squared prediction errors of 69,500 individuals in the prediction set. For genetic distance and Townsend Deprivation Index, individuals are binned into 5 equidistant strata, and the x-axis shows the median measure value for each stratum. Household income refers to the average yearly total household income before tax, and the 5 strata are taken directly from UKB data field 738. No repeated measurements from the same individual were taken.

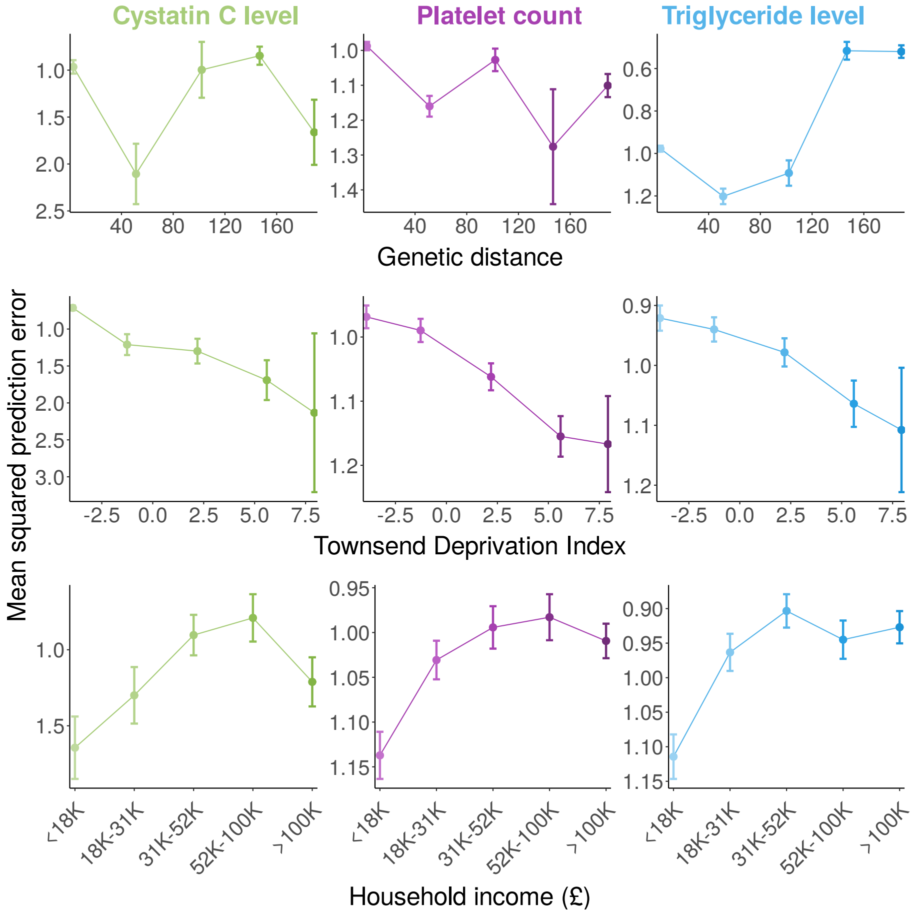

Supplementary Figure 17: Mean trends in individual-level prediction accuracy by different measures for other biomarkers. This figure presents the same analysis as Fig. 3A in the main text, but shows different traits: cystatin C level, platelet count, and triglyceride level. The y-axis corresponds to the mean (±SE) squared prediction errors of 69,500 individuals in the prediction set. For genetic distance and Townsend Deprivation Index, individuals are binned into 5 equidistant strata, and the x-axis shows the median measure value for each stratum. Household income refers to the average yearly total household income before tax, and the 5 strata are taken directly from UKB data field 738. No repeated measurements from the same individual were taken.

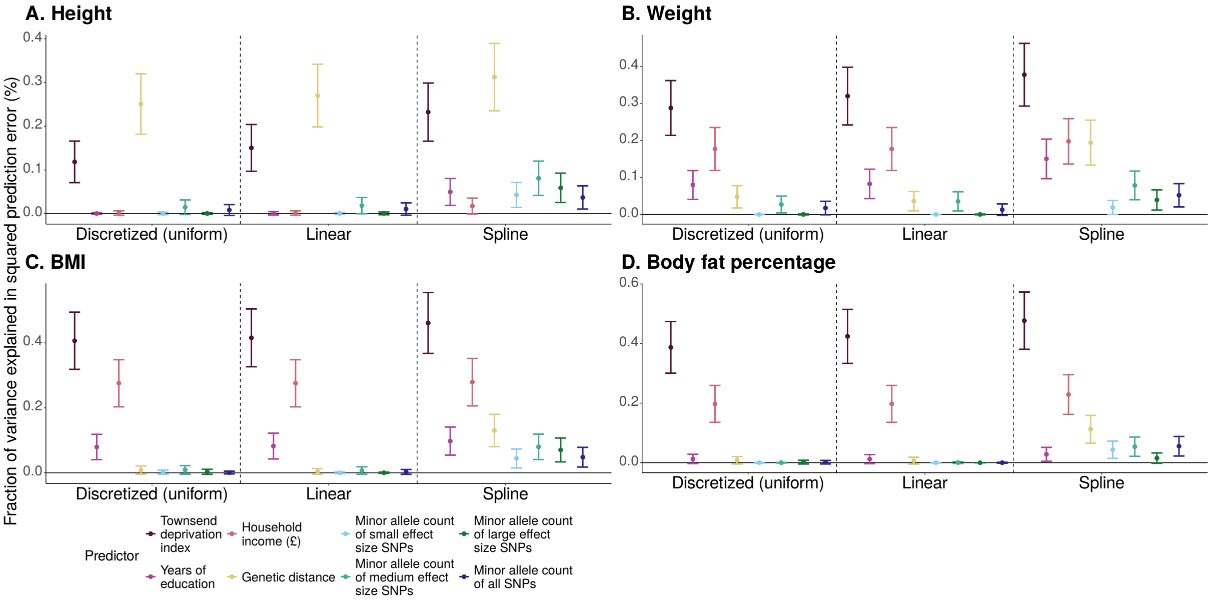

Supplementary Figure 18: Individual-level prediction error explained by different measures for anthropometric measurements. This figure presents the same analysis as Fig. 3B in the main text, but shows more measures and methods for fitting the measures, and focusing on anthropometric traits. Minor allele refers to the minor allele with respect to the GWAS sample. For each measure, we independently fit three different models. Discretized (uniform) refers to a discretized predictor, using one predicted value per each of the 5 bins where all 5 bins had identical widths. Linear refers to a linear predictor, fit using Ordinary Least Squares (OLS). Spline refers to a cubic spline, for which 16 knots were placed based on the density of data points, such that there was an equal number of data points between each pair of consecutive knots. All analyses were performed on the 69,500 prediction individuals. For all panels, no repeated measurements from the same individual were taken.

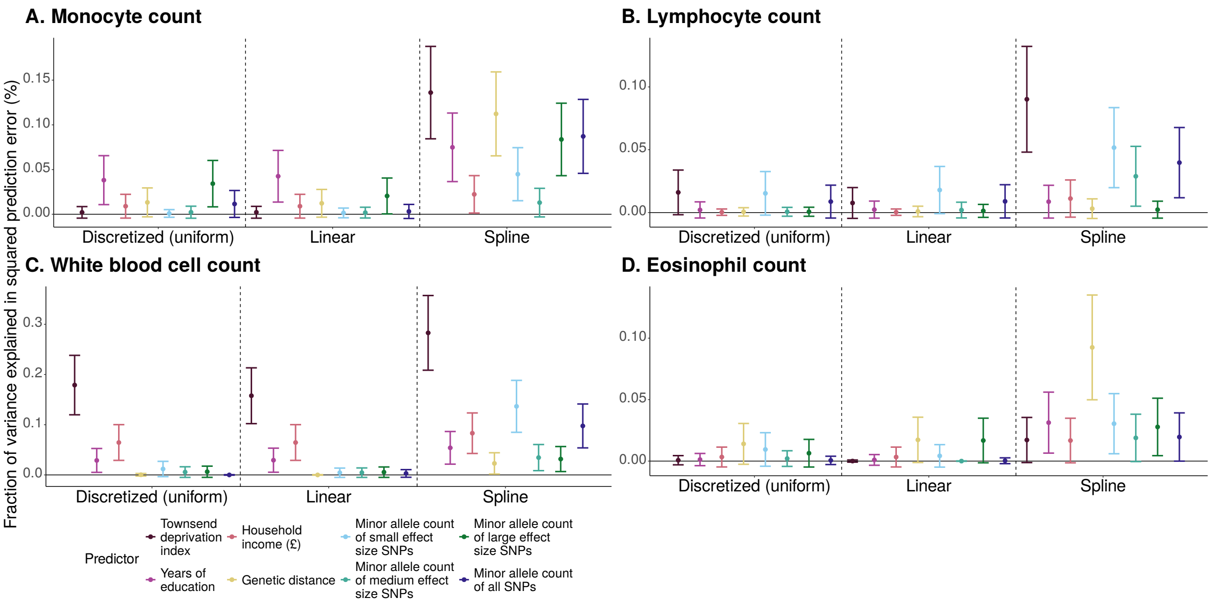

Supplementary Figure 19: Individual-level prediction error explained by different measures for white blood cell-related traits. This figure presents the same analysis as Fig. 3B in the main text, but shows more measures and methods for fitting the measures. Minor allele refers to the minor allele with respect to the GWAS sample. For each measure, we independently fit three different models. Discretized (uniform) refers to a discretized predictor, using one predicted value per each of the 5 bins where all 5 bins had identical widths. Linear refers to a linear predictor, fit using Ordinary Least Squares (OLS). Spline refers to a cubic spline, for which 16 knots were placed based on the density of data points, such that there was an equal number of data points between each pair of consecutive knots. All analyses were performed on the 69,500 prediction individuals. For all panels, no repeated measurements from the same individual were taken.

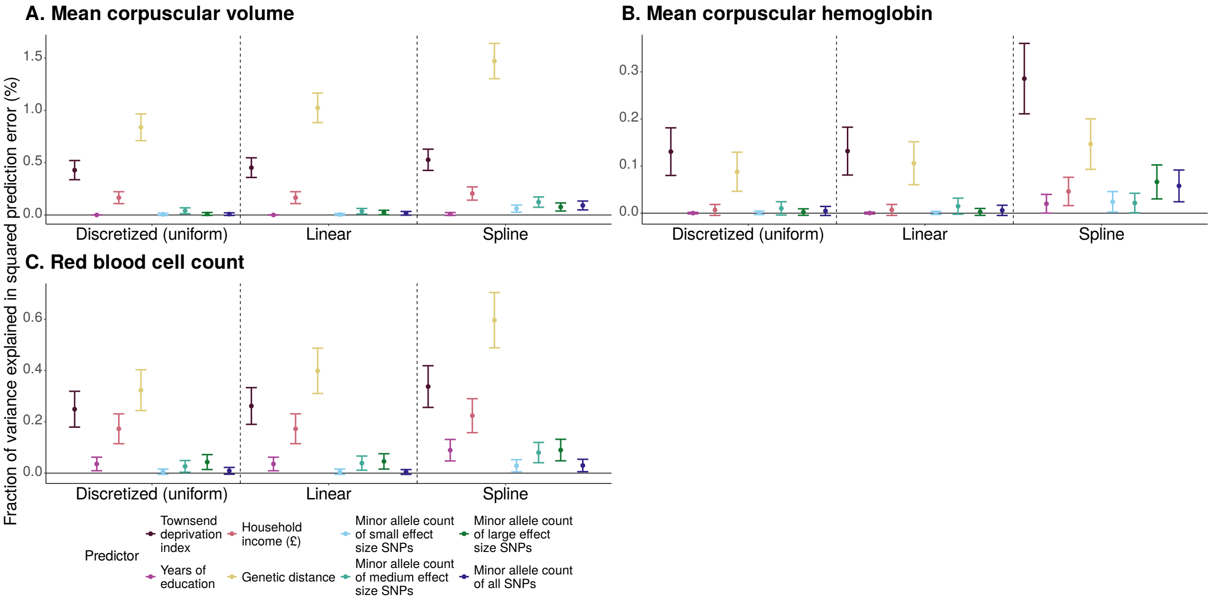

Supplementary Figure 20: Individual-level prediction error explained by different measures for red blood cell-related traits. This figure presents the same analysis as Fig. 3B in the main text, but shows more measures and methods for fitting the measures. Minor allele refers to the minor allele with respect to the GWAS sample. For each measure, we independently fit three different models. Discretized (uniform) refers to a discretized predictor, using one predicted value per each of the 5 bins where all 5 bins had identical widths. Linear refers to a linear predictor, fit using Ordinary Least Squares (OLS). Spline refers to a cubic spline, for which 16 knots were placed based on the density of data points, such that there was an equal number of data points between each pair of consecutive knots. All analyses were performed on the 69,500 prediction individuals. For all panels, no repeated measurements from the same individual were taken.

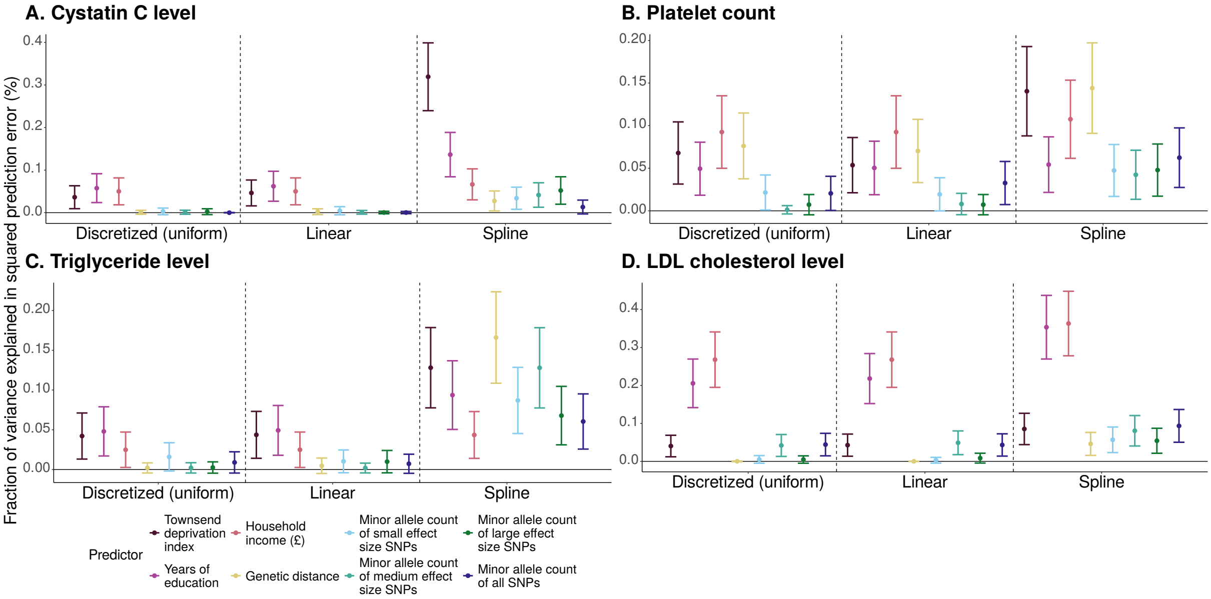

Supplementary Figure 21: Individual-level prediction error explained by different measures for other biomarkers. This figure presents the same analysis as Fig. 3B in the main text, but shows more measures and methods for fitting the measures. Minor allele refers to the minor allele with respect to the GWAS sample. For each measure, we independently fit three different models. Discretized (uniform) refers to a discretized predictor, using one predicted value per each of the 5 bins where all 5 bins had identical widths. Linear refers to a linear predictor, fit using Ordinary Least Squares (OLS). Spline refers to a cubic spline, for which 16 knots were placed based on the density of data points, such that there was an equal number of data points between each pair of consecutive knots. All analyses were performed on the 69,500 prediction individuals. For all panels, no repeated measurements from the same individual were taken.

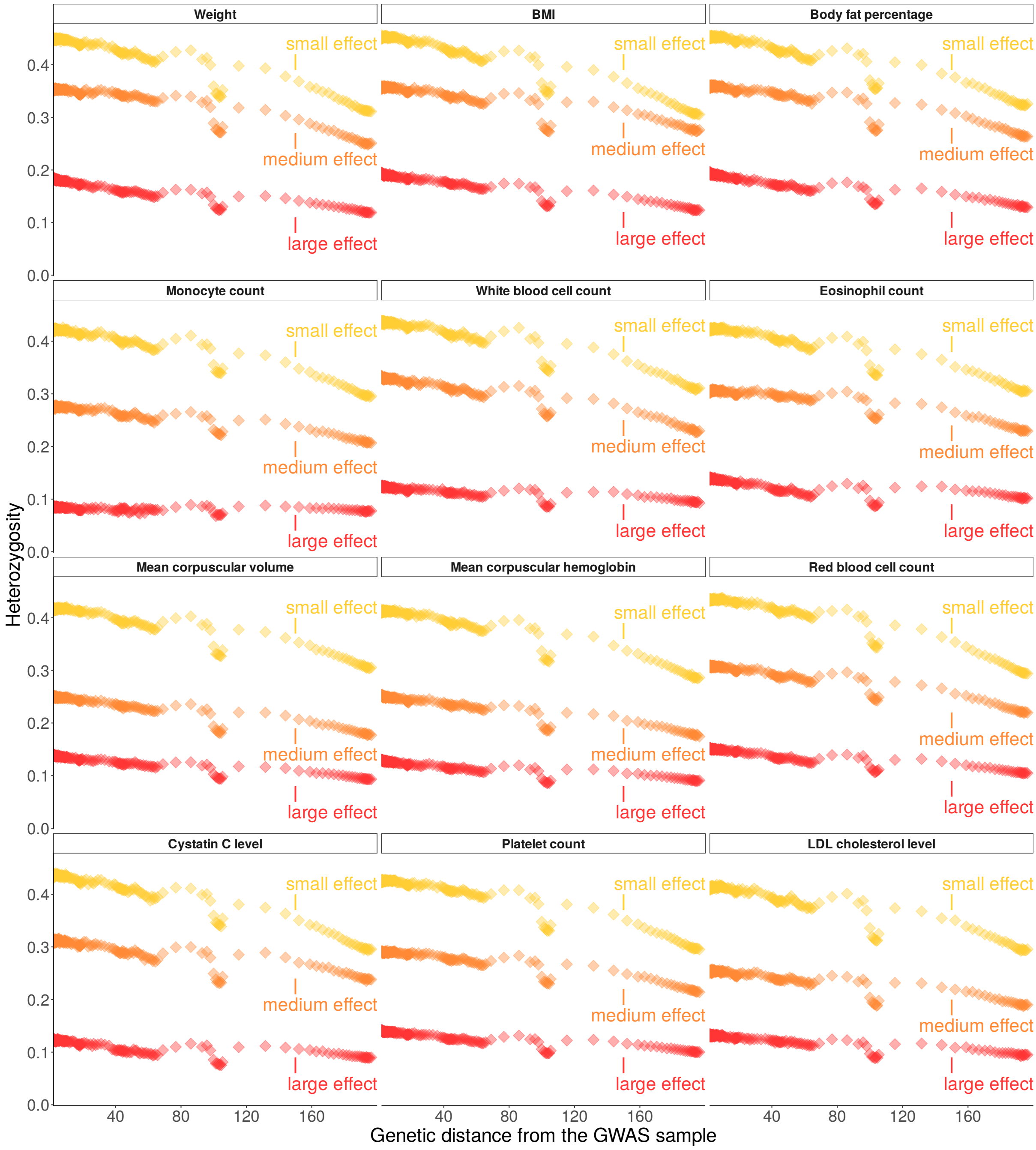

Supplementary Figure 22: Mean heterozygosity of SNPs, stratified by effect size. This figure presents the same analysis as Fig. 4B in the main text, but for the 12 quantitative traits that we analyzed which are not included there. For each trait, SNPs are stratified into three equal-sized strata (small, medium, and large) based on squared effect sizes (**Supplementary Fig. 23**). Each data point is the mean heterozygosity of a stratum in a bin of genetic distance. No repeated measurements from the same individual were taken.

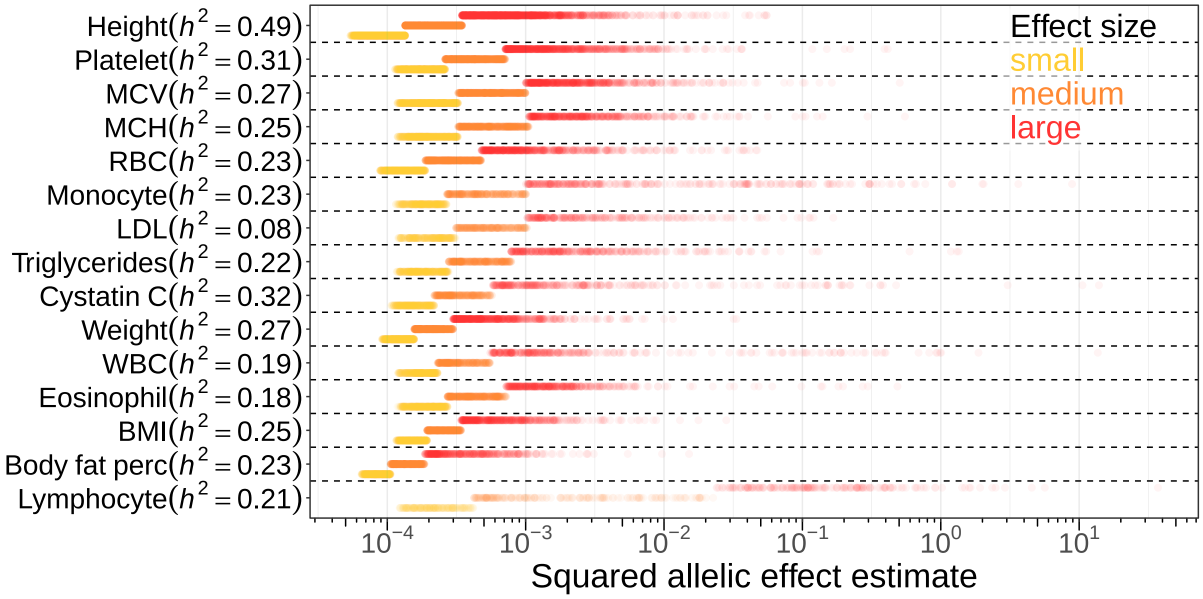

Supplementary Figure 23: Squared allelic effect estimate. Among the 69,500 prediction individuals, for each trait, the index SNPs of the respective PGS are stratified into three equal-sized strata (small, medium, and large) based on squared effect sizes. The x-axis represents the squared effect sizes in units of trait variance in the GWAS set. SNP heritabilities (h^2) are taken from the Neale Lab's UKB analysis[10]. Each data point represents a SNP. No repeated measurements from the same individual were taken. MCV: mean corpuscular volume. MCH: mean corpuscular hemoglobin. RBC: red blood cell count. Body fat perc: body fat percentage. WBC: white blood cell count. LDL: LDL cholesterol level.

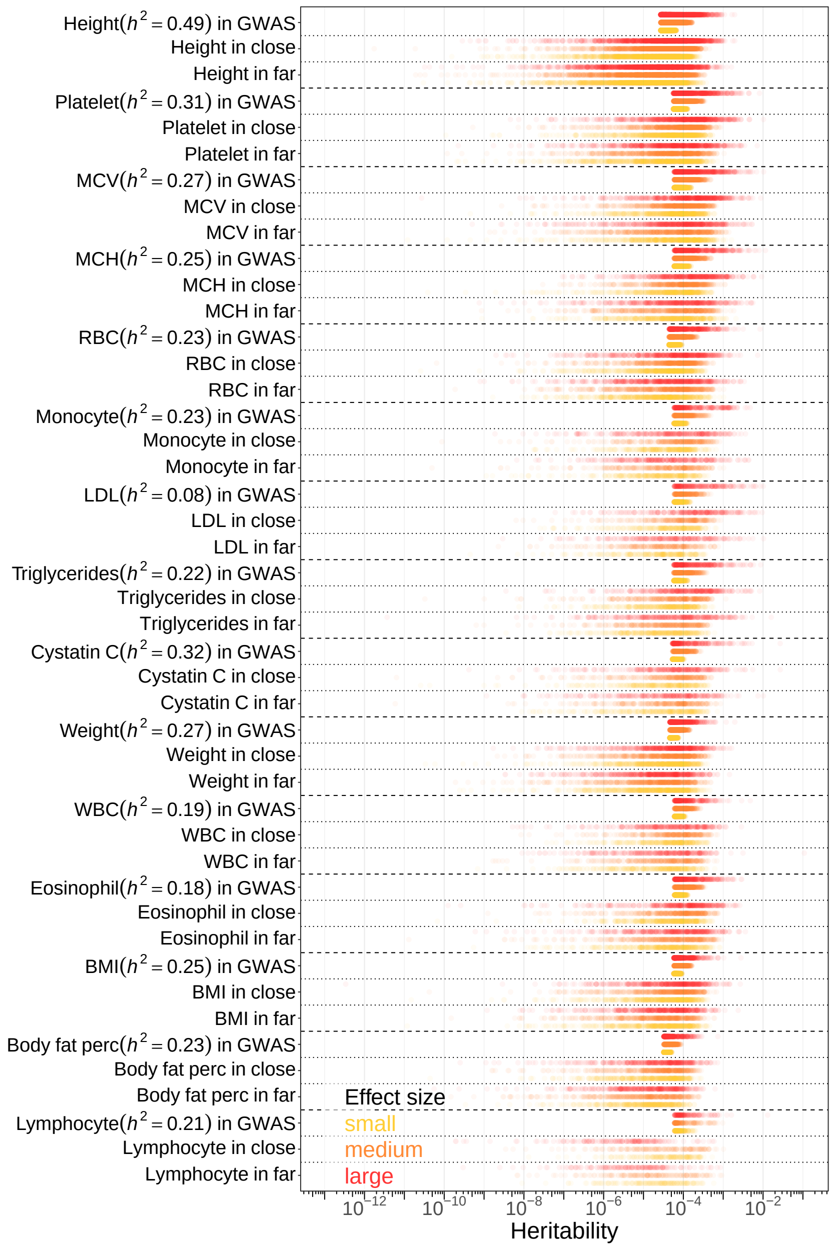

Supplementary Figure 24: Heritability explained by index SNPs. For each trait, index SNPs of the respective PGS are stratified by their squared effect sizes. Data points correspond to index SNPs. In each of the GWAS sample, the close subset of the prediction sample (genetic distance ≤ 10 in the prediction set, with 38,992 individuals) and the far subsample of the prediction sample (genetic distance > 10, with 30,508 individuals), we estimate the allelic effect of each SNP (in units of trait standard deviations) and its heterozygosity. The product of the two is the estimated heritability. SNP heritability estimates (h^2) on the y-axis are taken from the Neale Lab's UKB analysis[10]. No repeated measurements from the same individual were taken. MCV: mean corpuscular volume. MCH: mean corpuscular hemoglobin. RBC: red blood cell count. Body fat perc: body fat percentage. WBC: white blood cell count. LDL: LDL cholesterol level.

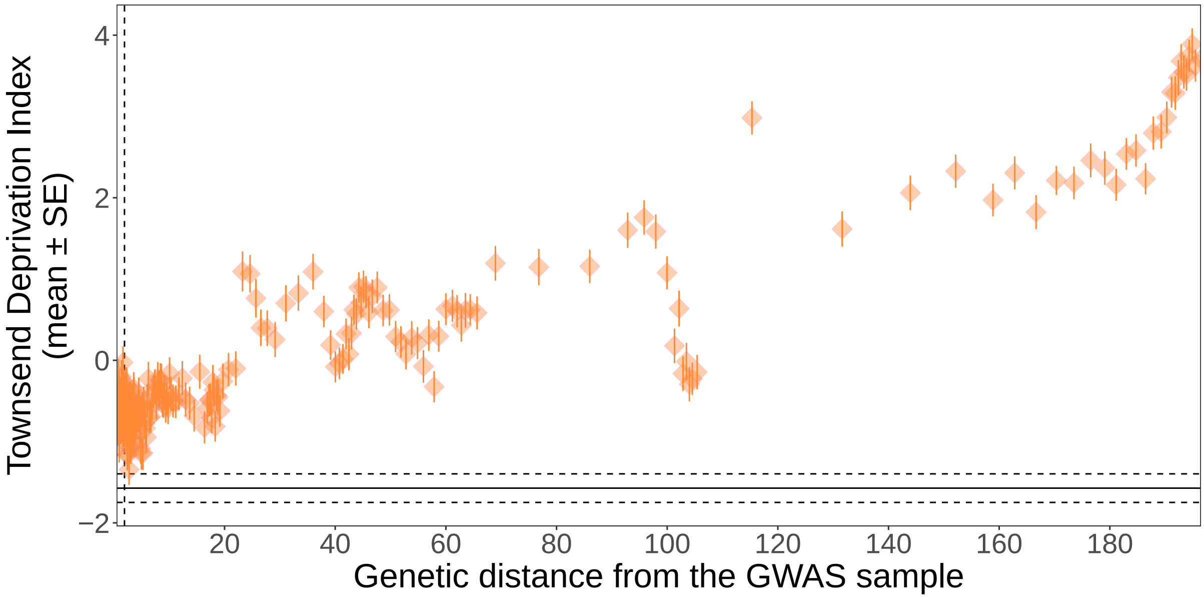

Supplementary Figure 25: Mean Townsend Deprivation Index ± standard error (SE) in each bin by genetic distance. In this figure, the orange dot and line represent the mean Townsend Deprivation Index ± SE in each bin in the prediction set. The horizontal solid line represents the mean Townsend Deprivation Index in the GWAS set. The horizontal dashed lines are calculated as mean in the GWAS sample $\pm \frac{SD_{GWAS}}{\sqrt{\overline{n_{pred}}}}$, where $\overline{n_{pred}}$ represents the mean sample size per bin (278 individuals), and there are 250 bins in the prediction set in total. The vertical dashed line represents the 97.5^{th} percentile of genetic distance among GWAS sample individuals. No repeated measurements from the same individual were taken.

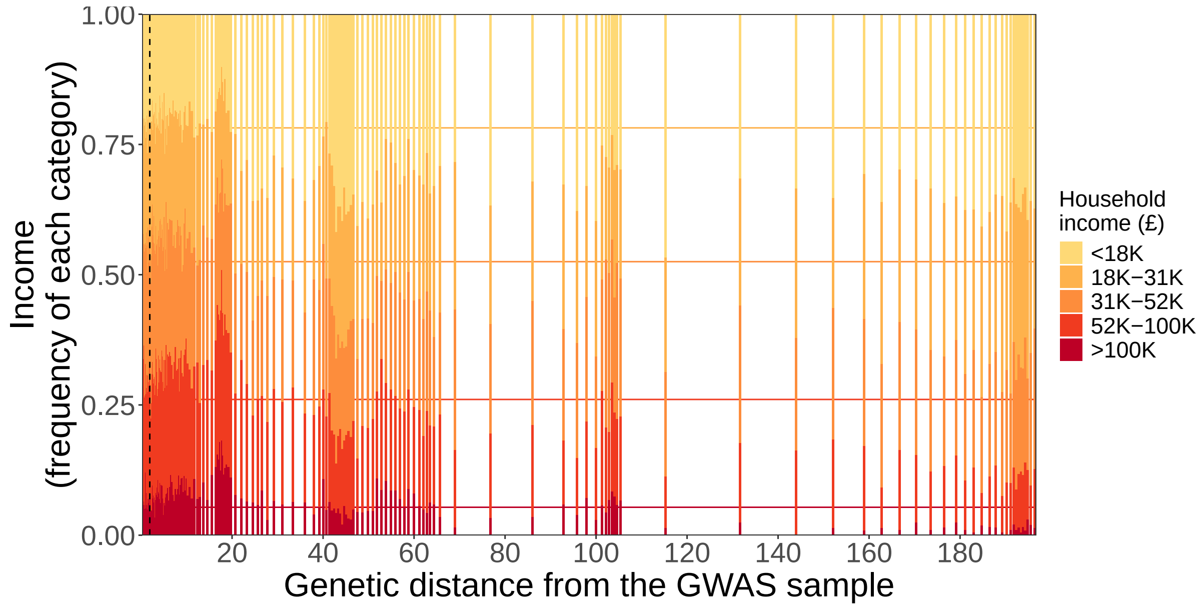

Supplementary Figure 26: Average total household income before tax in each bin by genetic distance. In this figure, each vertical colored bar represents one of 250 bins, each containing 278 individuals in the prediction set, with the cumulative frequency of income category color-coded. The horizontal colored lines represent the cumulative frequencies in the GWAS set. The vertical dashed line represents the 97.5^{th} percentile of genetic distance among GWAS sample individuals. No repeated measurements from the same individual were taken.

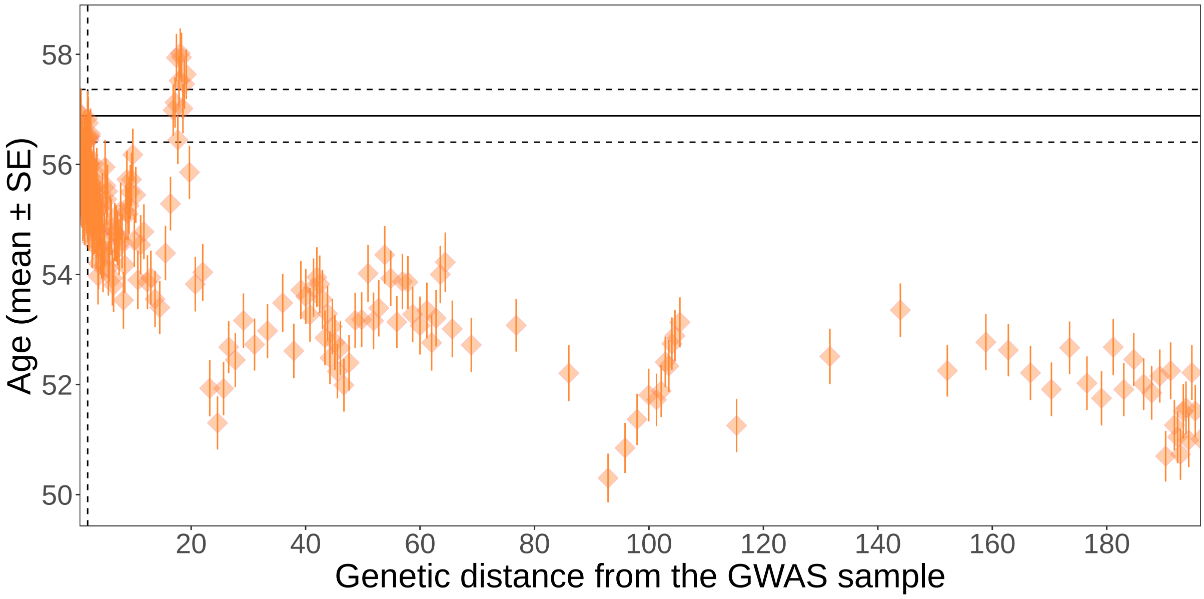

Supplementary Figure 27: Mean age ± SE in each bin by genetic distance. In this figure, the orange dot and line represent the mean age ± standard error (SE) in each bin in the prediction set. The horizontal solid line represents the mean age in the GWAS set. The horizontal dashed lines are calculated as mean in the GWAS sample ± $\frac{\text{SD}_{\text{GWAS}}}{\sqrt{\overline{n_{\text{pred}}}}}$, where $\overline{n_{\text{pred}}}$ represents the mean sample size per bin (278 individuals), and there are 250 bins in the prediction set in total. The vertical dashed line represents the 97.5th percentile of genetic distance among GWAS sample individuals. No repeated measurements from the same individual were taken.

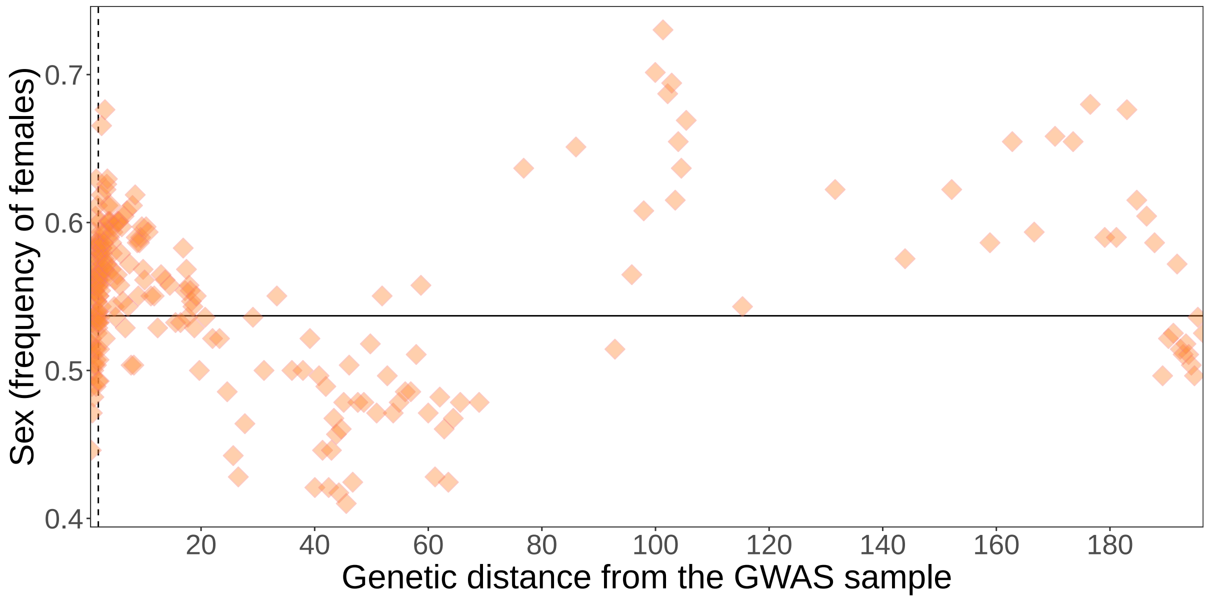

Supplementary Figure 28: Sex composition across bins of genetic distance. Orange dots represent the mean frequency of females in each genetic distance bin of 278 individuals, and there are 250 bins in the prediction set in total. The horizontal solid black line represents the mean frequency of females in the GWAS set. The vertical dashed line represents the 97.5th percentile of genetic distance among GWAS sample individuals. No repeated measurements from the same individual were taken.

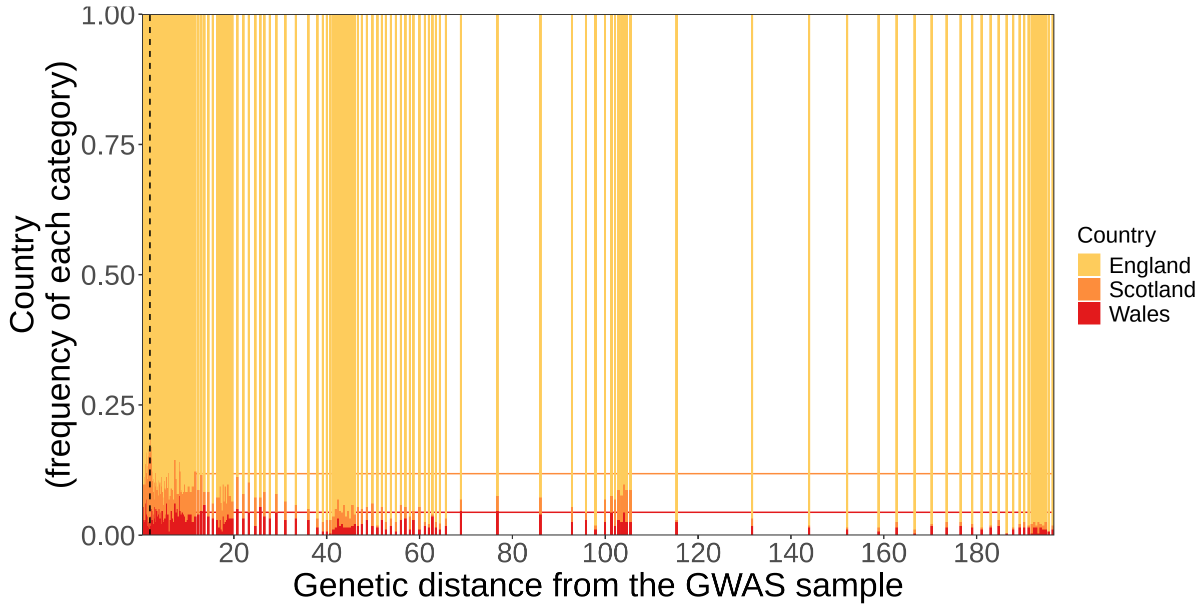

Supplementary Figure 29: Participants' country of residence across bins of genetic distance. In this figure, each vertical colored bar represents one of 250 bins, each containing 278 individuals in the prediction set, with the cumulative frequency of country of residence color-coded. The horizontal colored lines represent the cumulative frequencies in the GWAS set. The vertical dashed line represents the 97.5^{th} percentile of genetic distance among GWAS sample individuals. No repeated measurements from the same individual were taken.

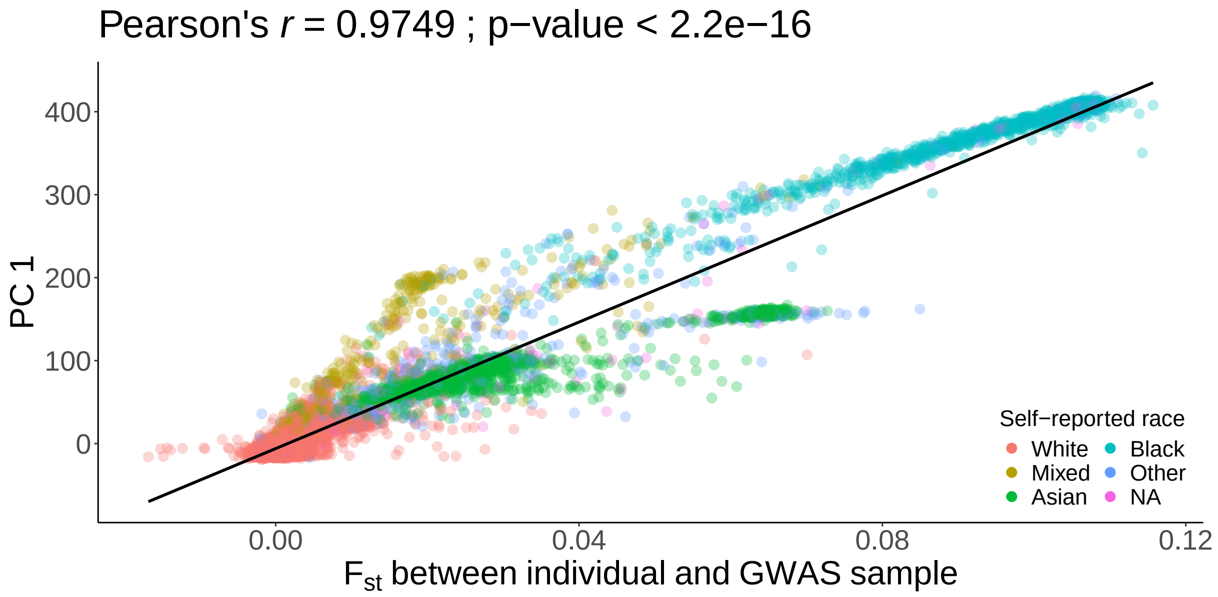

Supplementary Figure 30: Correlation between F_{st} and PC 1. This figure presents the correlation between F_{st} and PC 1 for 10,000 randomly selected prediction sample individuals (among those with a weighted PC distance greater than the weighted PC distance of 95% of the GWAS set; as described in Methods). The data points are colored by self-reported race (UKB data field 21000). No repeated measurements from the same individual were taken.

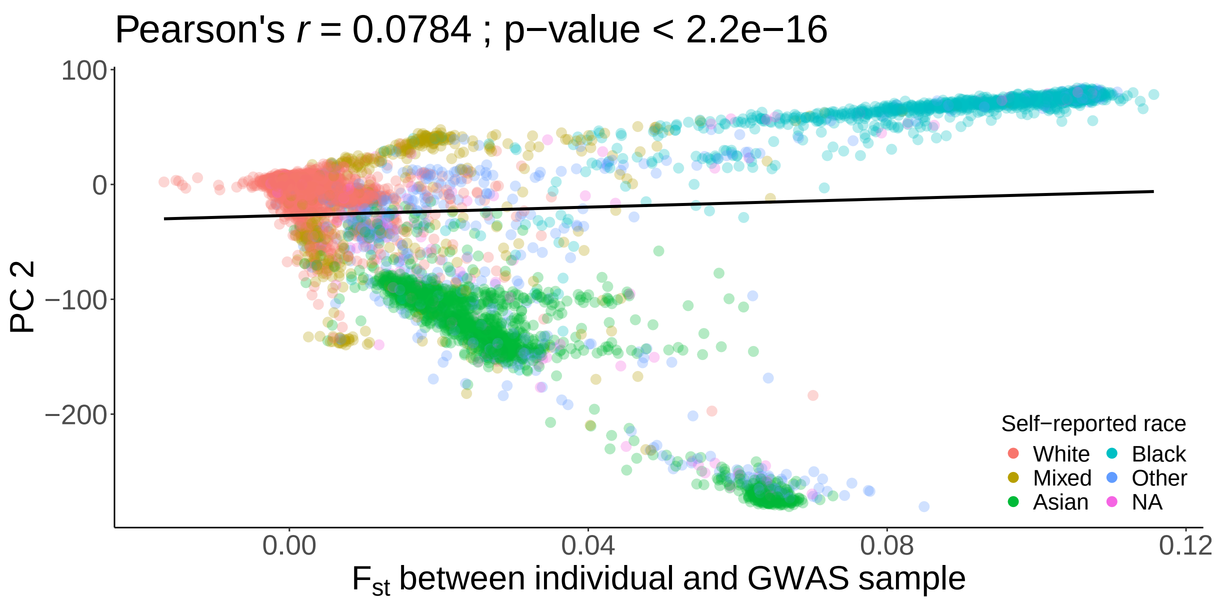

Supplementary Figure 31: Correlation between F_{st} and PC 2. This figure presents the correlation between F_{st} and PC 2 for 10,000 randomly selected prediction sample individuals (among those with a weighted PC distance greater than the weighted PC distance of 95% of the GWAS set; as described in Methods). The data points are colored by self-reported race (UKB data field 21000). No repeated measurements from the same individual were taken.

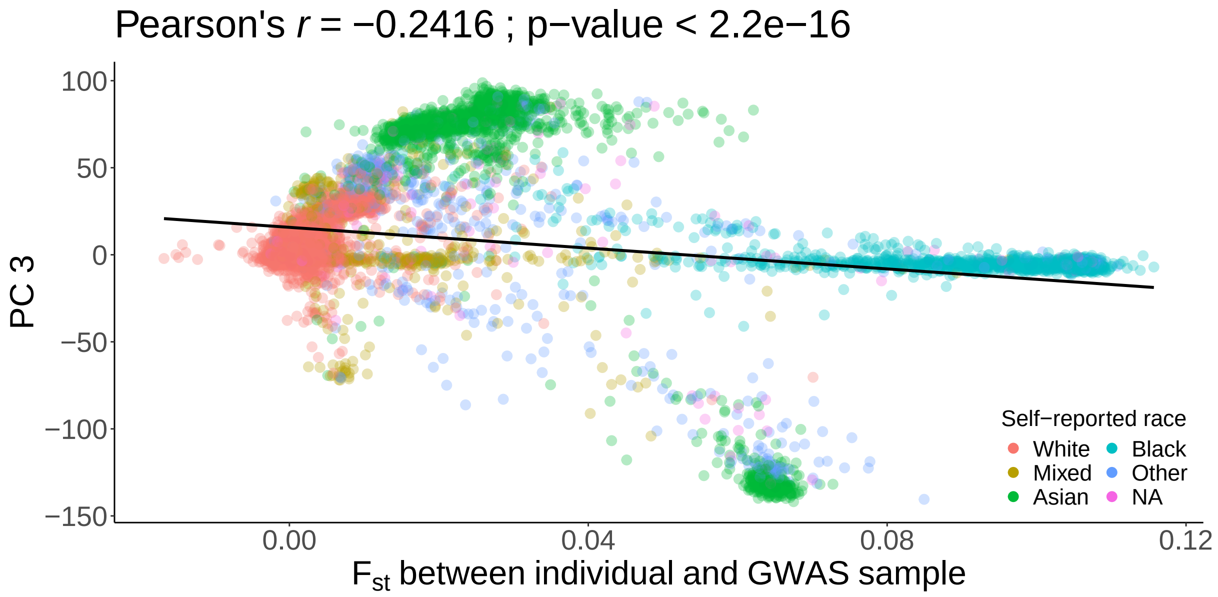

Supplementary Figure 32: Correlation between F_{st} and PC 3. This figure presents the correlation between F_{st} and PC 3 for 10,000 randomly selected prediction sample individuals (among those with a weighted PC distance greater than the weighted PC distance of 95% of the GWAS set; as described in Methods). The data points are colored by self-reported race (UKB data field 21000). No repeated measurements from the same individual were taken.

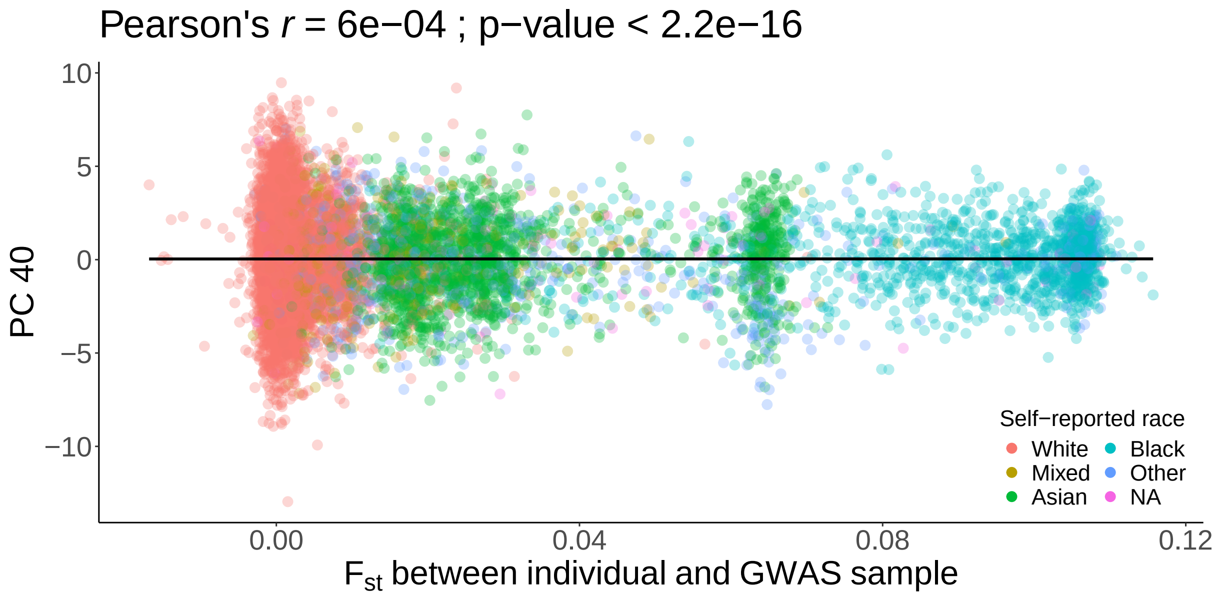

Supplementary Figure 33: Correlation between F_{st} and PC 40. This figure presents the correlation between F_{st} and PC 40 for 10,000 randomly selected prediction sample individuals (among those with a weighted PC distance greater than the weighted PC distance of 95% of the GWAS set; as described in Methods). The data points are colored by self-reported race (UKB data field 21000). No repeated measurements from the same individual were taken.

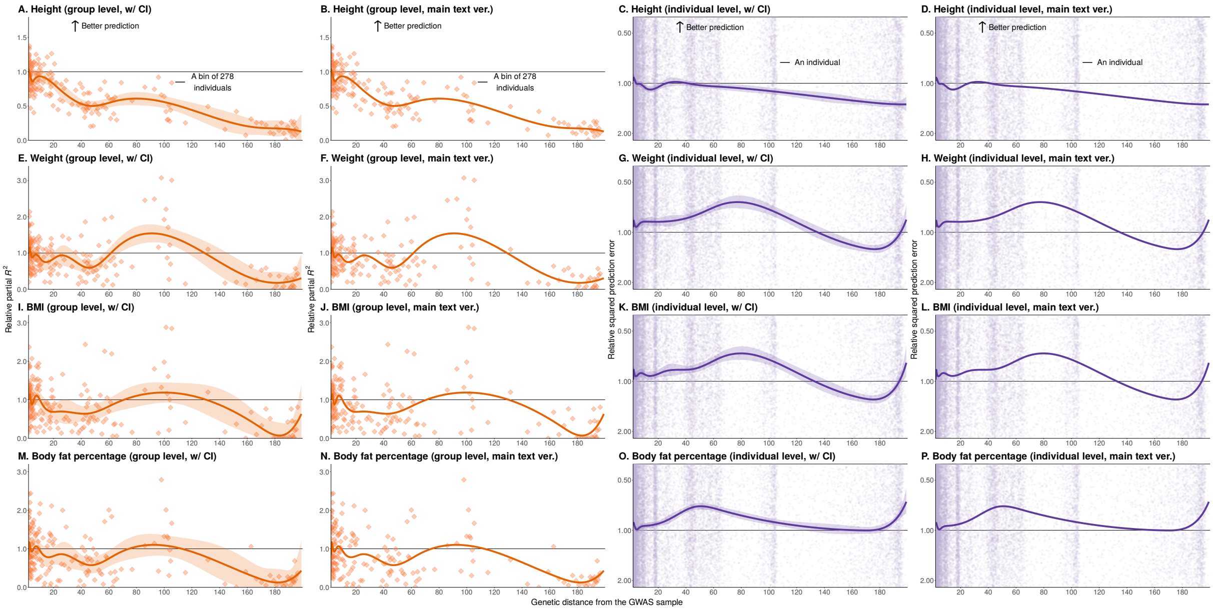

Supplementary Figure 34: Trends of portability vary across traits and measures, with confidence intervals (CI), for anthropometric measurements. This figure presents the same analysis as Fig. 2 and Supplementary Fig. 2, but the first and third columns (A, C, E, G, I, K, M, O) are plots with confidence intervals. The second and fourth columns (B, D, F, H, J, L, N, P) contain plots taken from the main text or supplementary text for comparison. At the group level (left two columns), we measured prediction accuracy with the squared partial correlation between the PGS and the trait value in 250 bins of 278 individuals each. At the individual level (right two columns), we measured prediction accuracy as the squared difference between the predicted phenotype and the true phenotype value. At both the group and individual levels, y-axis values show relative prediction accuracy, i.e. prediction accuracy divided by a baseline value. The baseline value is the mean prediction accuracy in 25 bins with median genetic distances that are closest to the mean genetic distance for GWAS individuals. Supplementary Table 1 details these baseline values for each trait. Curves show cubic spline fits, with 8 knots placed based on the density of data points. For all panels, no repeated measurements from the same individual were taken.

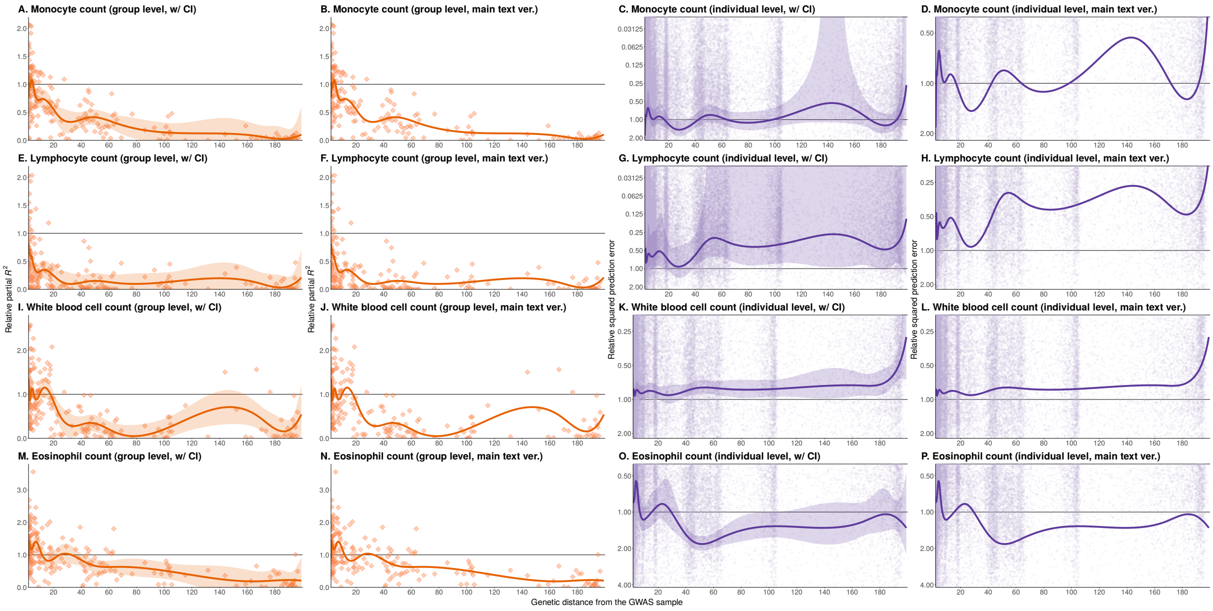

Supplementary Figure 35: Trends of portability vary across traits and measures, with confidence intervals (CI), for white blood cell-related traits. This figure presents the same analysis as Fig. 2 and Supplementary Fig. 3, but the first and third columns (A, C, E, G, I, K, M, O) are plots with confidence intervals. The second and fourth columns (B, D, F, H, J, L, N, P) contain plots taken from the main text or supplementary text for comparison. At the group level (left two columns), we measured prediction accuracy with the squared partial correlation between the PGS and the trait value in 250 bins of 278 individuals each. At the individual level (right two columns), we measured prediction accuracy as the squared difference between the predicted phenotype and the true phenotype value. At both the group and individual levels, y-axis values show relative prediction accuracy, i.e. prediction accuracy divided by a baseline value. The baseline value is the mean prediction accuracy in 25 bins with median genetic distances that are closest to the mean genetic distance for GWAS individuals. Supplementary Table 1 details these baseline values for each trait. Curves show cubic spline fits, with 8 knots placed based on the density of data points. For C, G, K, when plotting the CI on a logarithmic scale, we replaced the values extremely close to zero with 10^{-6}. For all panels, no repeated measurements from the same individual were taken.

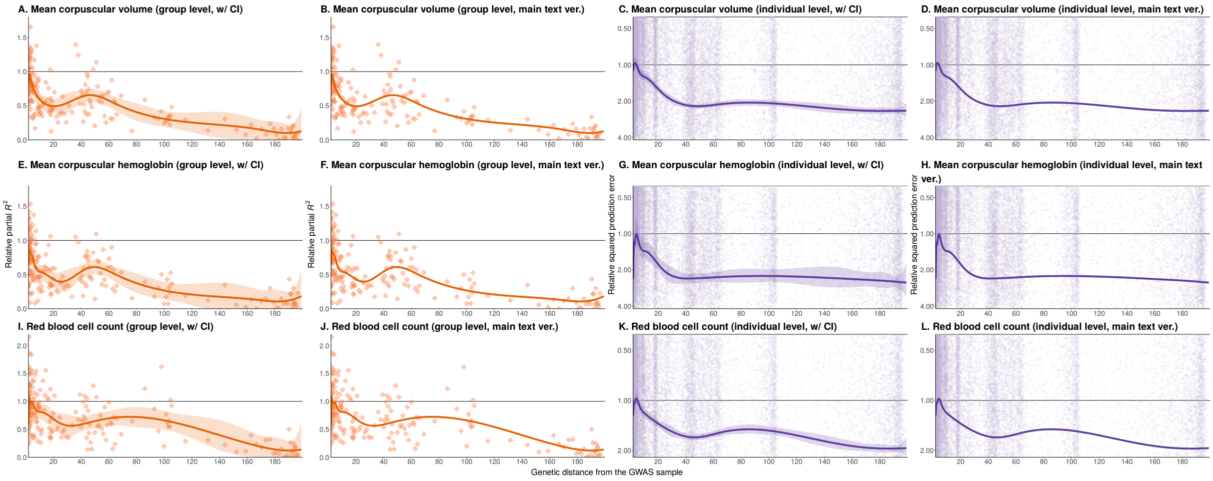

Supplementary Figure 36: Trends of portability vary across traits and measures, with confidence intervals (CI), for red blood cell-related traits. This figure presents the same analysis as . 2 and Supplementary Fig. 4, but the first and third columns (A, C, E, G, I, K) are plots with confidence intervals. The second and fourth columns (B, D, F, H, J, L) contain plots taken from the main text or supplementary text for comparison. At the group level (left two columns), we measured prediction accuracy with the squared partial correlation between the PGS and the trait value in 250 bins of 278 individuals each. At the individual level (right two columns), we measured prediction accuracy as the squared difference between the predicted phenotype and the true phenotype value. At both the group and individual levels, y-axis values show relative prediction accuracy, i.e. prediction accuracy divided by a baseline value. The baseline value is the mean prediction accuracy in 25 bins with median genetic distances that are closest to the mean genetic distance for GWAS individuals. Supplementary Table 1 details these baseline values for each trait. Curves show cubic spline fits, with 8 knots placed based on the density of data points. For all panels, no repeated measurements from the same individual were taken.

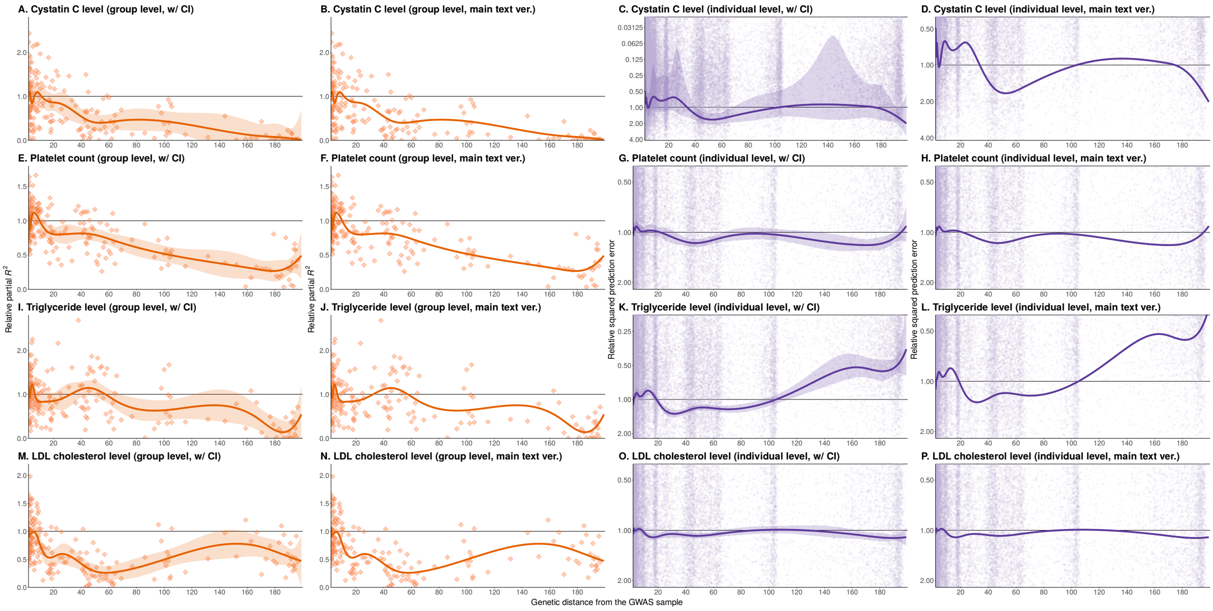

Supplementary Figure 37: Trends of portability vary across traits and measures, with confidence intervals (CI), for other biomarkers. This figure presents the same analysis as Fig. 2 and Supplementary Fig. 5, but the first and third columns (A, C, E, G, I, K, M, O) are plots with 95% confidence intervals. The second and fourth columns (B, D, F, H, J, L, N, P) contain plots taken from the main text or supplementary text for comparison. At the group level (left two columns), we measured prediction accuracy with the squared partial correlation between the PGS and the trait value in 250 bins of 278 individuals each. At the individual level (right two columns), we measured prediction accuracy as the squared difference between the predicted phenotype and the true phenotype value. At both the group and individual levels, y-axis values show relative prediction accuracy, i.e. prediction accuracy divided by a baseline value. The baseline value is the mean prediction accuracy in 25 bins with median genetic distances that are closest to the mean genetic distance for GWAS individuals. Supplementary Table 1 details these baseline values for each trait. Curves show cubic spline fits, with 8 knots placed based on the density of data points. For all panels, no repeated measurements from the same individual were taken.

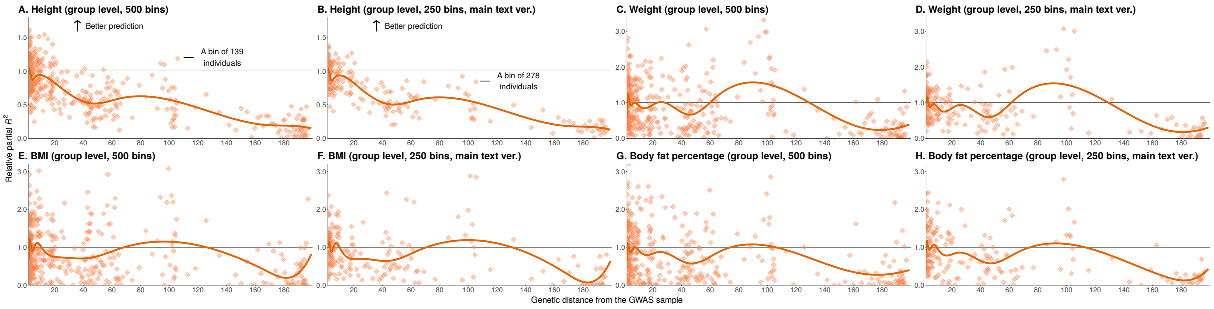

Supplementary Figure 38: Group level portability, using more bins, for anthropometric measurements. This figure presents the same analysis as Fig. 2 and Supplementary Fig. 2 at the group level, but the first and third columns (A, C, E, G) are plots with 500 bins of 139 each. The second and fourth columns (B, D, F, H) contain plots with 250 bins of 278 each, taken from the main text or supplementary text for comparison. At the group level, we measured prediction accuracy with the squared partial correlation between the PGS and the trait value. The y-axis values show relative prediction accuracy, i.e. prediction accuracy divided by a baseline value. The baseline value for the panels with 500 bins is the mean prediction accuracy in 50 bins with median genetic distances that are closest to the mean genetic distance for GWAS individuals, and is based on 25 bins with median genetic distances that are closest to the mean genetic distance for GWAS individuals for the original plots with 250 bins. Supplementary Table 1 details these baseline values for each trait. Curves show cubic spline fits, with 8 knots placed based on the density of data points. For all panels, no repeated measurements from the same individual were taken.

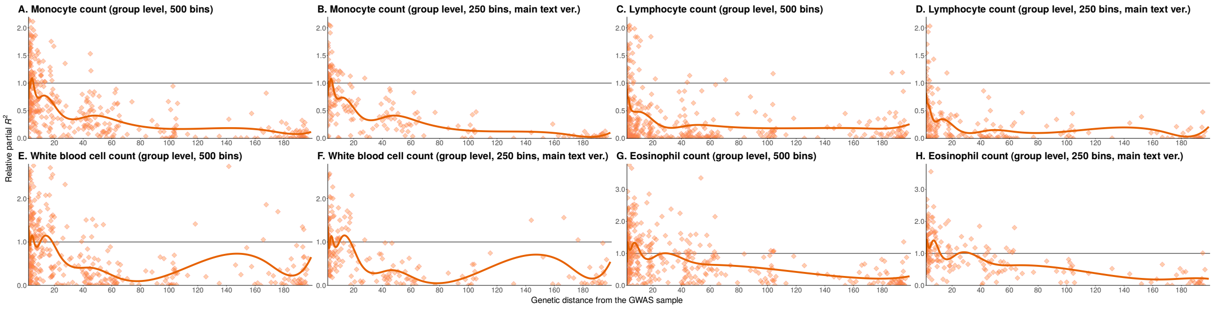

Supplementary Figure 39: Group level portability, using more bins, for white blood cell-related traits. This figure presents the same analysis as Fig. 2 and Supplementary Fig. 3 at the group level, but the first and third columns (A, C, E, G) are plots with 500 bins of 139 each. The second and fourth columns (B, D, F, H) contain plots with 250 bins of 278 each, taken from the main text or supplementary text for comparison. At the group level, we measured prediction accuracy with the squared partial correlation between the PGS and the trait value. The y-axis values show relative prediction accuracy, i.e. prediction accuracy divided by a baseline value. The baseline value for the panels with 500 bins is the mean prediction accuracy in 50 bins with median genetic distances that are closest to the mean genetic distance for GWAS individuals, and is based on 25 bins with median genetic distances that are closest to the mean genetic distance for GWAS individuals for the original plots with 250 bins. Supplementary Table 1 details these baseline values for each trait. Curves show cubic spline fits, with 8 knots placed based on the density of data points. For all panels, no repeated measurements from the same individual were taken.

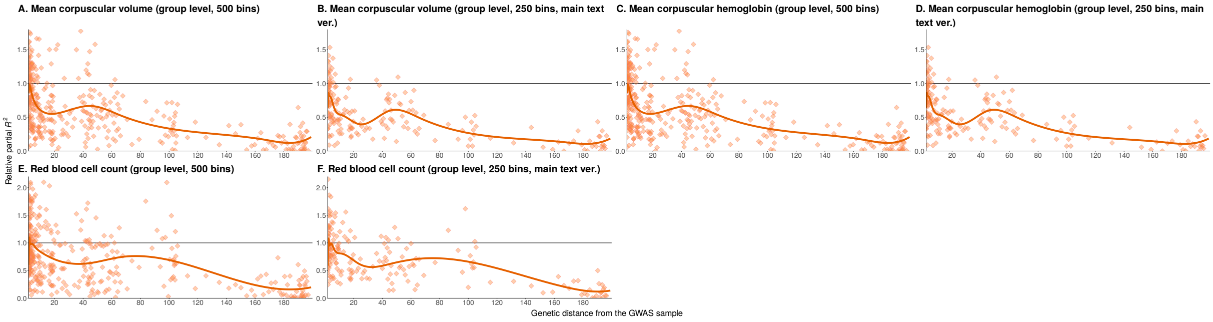

Supplementary Figure 40: Group level portability, using more bins, for red blood cell-related traits. This figure presents the same analysis as Fig. 2 and Supplementary Fig. 4 at the group level, but the first and third columns (A, C, E) are plots with 500 bins of 139 each. The second and fourth columns (B, D, F) contain plots with 250 bins of 278 each, taken from the main text or supplementary text for comparison. At the group level, we measured prediction accuracy with the squared partial correlation between the PGS and the trait value. The y-axis values show relative prediction accuracy, i.e. prediction accuracy divided by a baseline value. The baseline value for the panels with 500 bins is the mean prediction accuracy in 50 bins with median genetic distances that are closest to the mean genetic distance for GWAS individuals, and is based on 25 bins with median genetic distances that are closest to the mean genetic distance for GWAS individuals for the original plots with 250 bins. Supplementary Table 1 details these baseline values for each trait. Curves show cubic spline fits, with 8 knots placed based on the density of data points. For all panels, no repeated measurements from the same individual were taken.

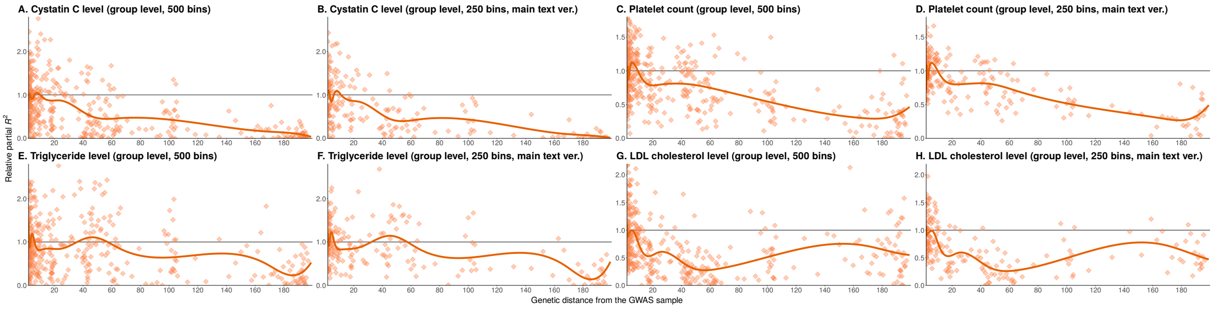

Supplementary Figure 41: Group level portability, using more bins, for other biomarkers. This figure presents the same analysis as Fig. 2 and Supplementary Fig. 5 at the group level, but the first and third columns (A, C, E, G) are plots with 500 bins of 139 each. The second and fourth columns (B, D, F, H) contain plots with 250 bins of 278 each, taken from the main text or supplementary text for comparison. At the group level, we measured prediction accuracy with the squared partial correlation between the PGS and the trait value. The y-axis values show relative prediction accuracy, i.e. prediction accuracy divided by a baseline value. The baseline value for the panels with 500 bins is the mean prediction accuracy in 50 bins with median genetic distances that are closest to the mean genetic distance for GWAS individuals, and is based on 25 bins with median genetic distances that are closest to the mean genetic distance for GWAS individuals for the original plots with 250 bins. Supplementary Table 1 details these baseline values for each trait. Curves show cubic spline fits, with 8 knots placed based on the density of data points. For all panels, no repeated measurements from the same individual were taken.

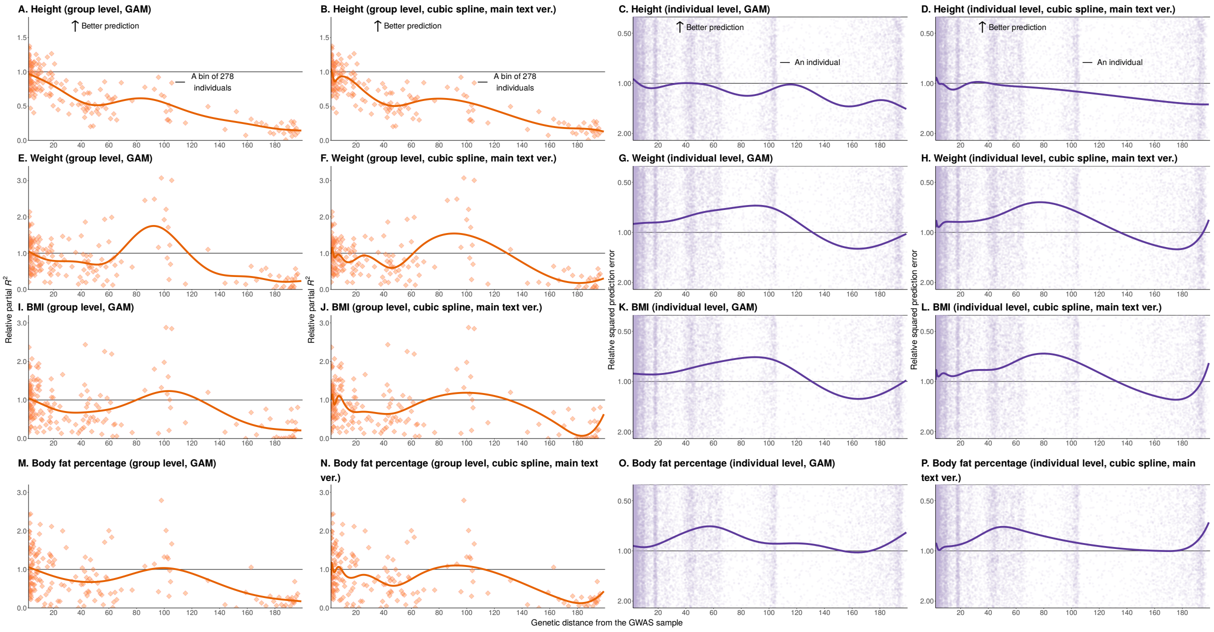

Supplementary Figure 42: Trends of portability, visualized with generalized additive model (GAM) fit, for anthropometric measurements. This figure presents the same analysis as Fig. 2 and Supplementary Fig. 2, but the first and third columns (A, C, E, G, I, K, M, O) are plots with GAM fits, as shown by the curves. The second and fourth columns (B, D, F, H, J, L, N, P) contain plots with cubic spline fits, taken from the main text or supplementary text for comparison. At the group level (left two columns), we measured prediction accuracy with the squared partial correlation between the PGS and the trait value in 250 bins of 278 individuals each. At the individual level (right two columns), we measured prediction accuracy as the squared difference between the predicted phenotype and the true phenotype value. At both the group and individual levels, y-axis values show relative prediction accuracy, i.e. prediction accuracy divided by a baseline value. The baseline value is the mean prediction accuracy in 25 bins with median genetic distances that are closest to the mean genetic distance for GWAS individuals. Supplementary Table 1 details these baseline values for each trait. For both GAM and cubic spline fits, curves show cubic spline fits, with 8 knots placed based on the density of data points. For all panels, no repeated measurements from the same individual were taken.

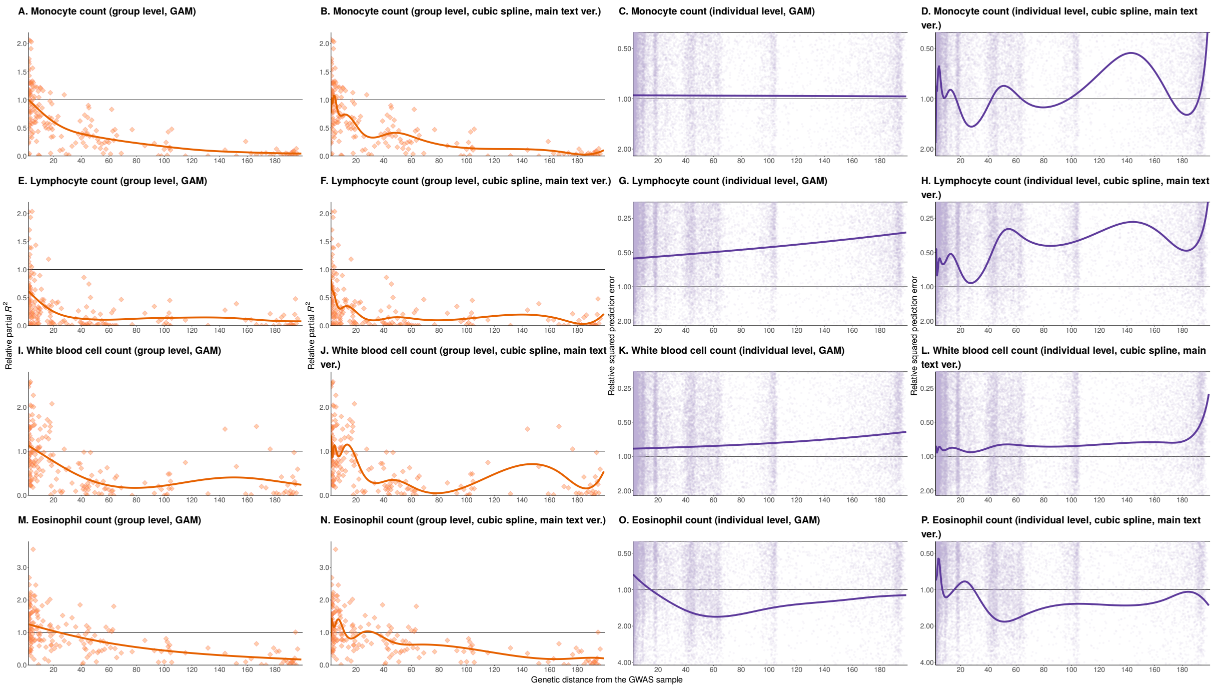

Supplementary Figure 43: Trends of portability, visualized with generalized additive model (GAM) fit, for white blood cell-related traits. This figure presents the same analysis as Fig. 2 and Supplementary Fig. 3, but the first and third columns (A, C, E, G, I, K, M, O) are plots with GAM fits, as shown by the curves. The second and fourth columns (B, D, F, H, J, L, N, P) contain plots with cubic spline fits, taken from the main text or supplementary text for comparison. At the group level (left two columns), we measured prediction accuracy with the squared partial correlation between the PGS and the trait value in 250 bins of 278 individuals each. At the individual level (right two columns), we measured prediction accuracy as the squared difference between the predicted phenotype and the true phenotype value. At both the group and individual levels, y-axis values show relative prediction accuracy, i.e. prediction accuracy divided by a baseline value. The baseline value is the mean prediction accuracy in 25 bins with median genetic distances that are closest to the mean genetic distance for GWAS individuals. Supplementary Table 1 details these baseline values for each trait. For both GAM and cubic spline fits, curves show cubic spline fits, with 8 knots placed based on the density of data points. For all panels, no repeated measurements from the same individual were taken.

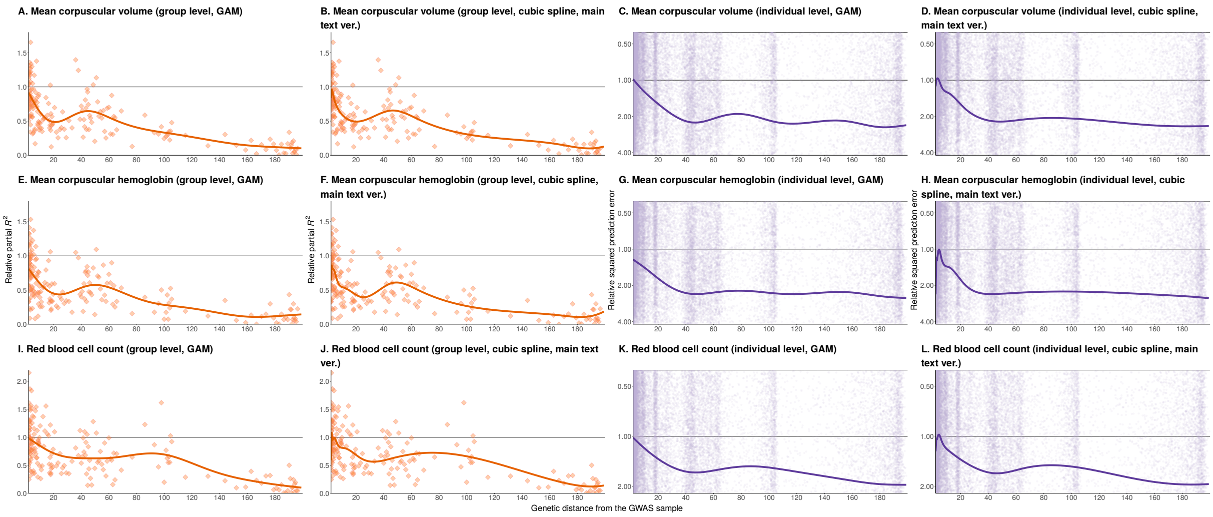

Supplementary Figure 44: Trends of portability, visualized with generalized additive model (GAM) fit, for red blood cell-related traits. This figure presents the same analysis as Fig. 2 and Supplementary Fig. 4, but the first and third columns (A, C, E, G, I, K) are plots with GAM fits, as shown by the curves. The second and fourth columns (B, D, F, H, J, L) contain plots with cubic spline fits, taken from the main text or supplementary text for comparison. At the group level (left two columns), we measured prediction accuracy with the squared partial correlation between the PGS and the trait value in 250 bins of 278 individuals each. At the individual level (right two columns), we measured prediction accuracy as the squared difference between the predicted phenotype and the true phenotype value. At both the group and individual levels, y-axis values show relative prediction accuracy, i.e. prediction accuracy divided by a baseline value. The baseline value is the mean prediction accuracy in 25 bins with median genetic distances that are closest to the mean genetic distance for GWAS individuals. Supplementary Table 1 details these baseline values for each trait. For both GAM and cubic spline fits, curves show cubic spline fits, with 8 knots placed based on the density of data points. For all panels, no repeated measurements from the same individual were taken.

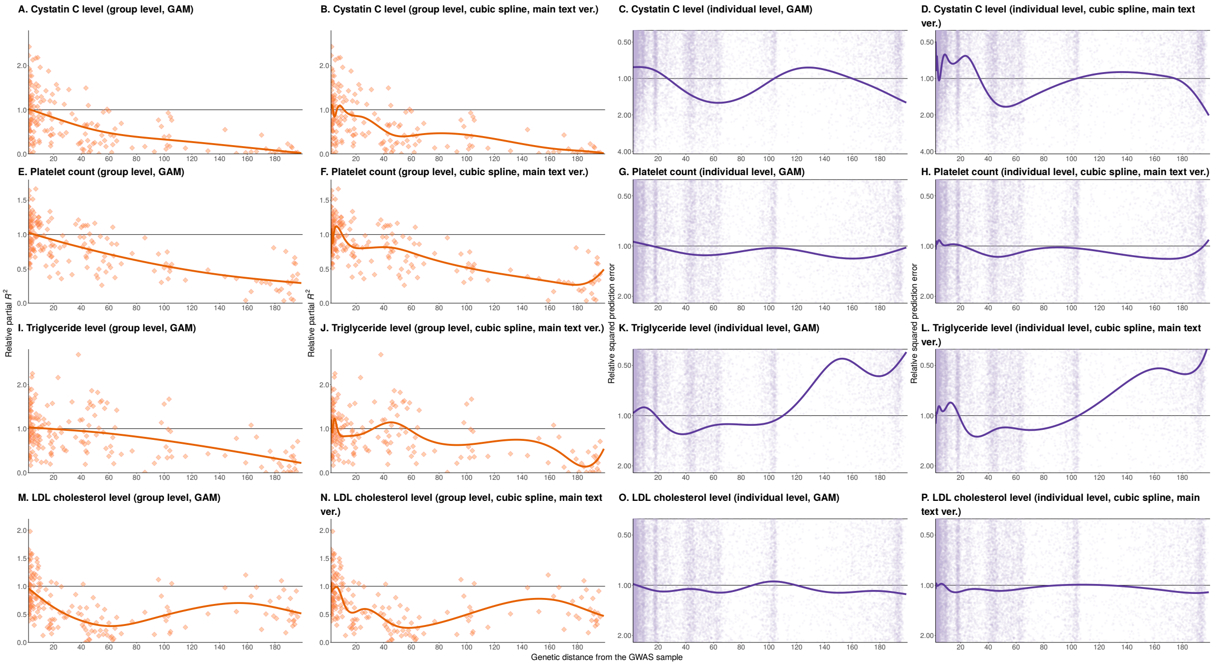

Supplementary Figure 45: Trends of portability, visualized with generalized additive model (GAM) fit, for other biomarkers. This figure presents the same analysis as Fig. 2 and Supplementary Fig. 5, but the first and third columns (A, C, E, G, I, K, M, O) are plots with GAM fits, as shown by the curves. The second and fourth columns (B, D, F, H, J, L, N, P) contain plots with cubic spline fits, taken from the main text or supplementary text for comparison. At the group level (left two columns), we measured prediction accuracy with the squared partial correlation between the PGS and the trait value in 250 bins of 278 individuals each. At the individual level (right two columns), we measured prediction accuracy as the squared difference between the predicted phenotype and the true phenotype value. At both the group and individual levels, y-axis values show relative prediction accuracy, i.e. prediction accuracy divided by a baseline value. The baseline value is the mean prediction accuracy in 25 bins with median genetic distances that are closest to the mean genetic distance for GWAS individuals. Supplementary Table 1 details these baseline values for each trait. For both GAM and cubic spline fits, curves show cubic spline fits, with 8 knots placed based on the density of data points. For all panels, no repeated measurements from the same individual were taken.

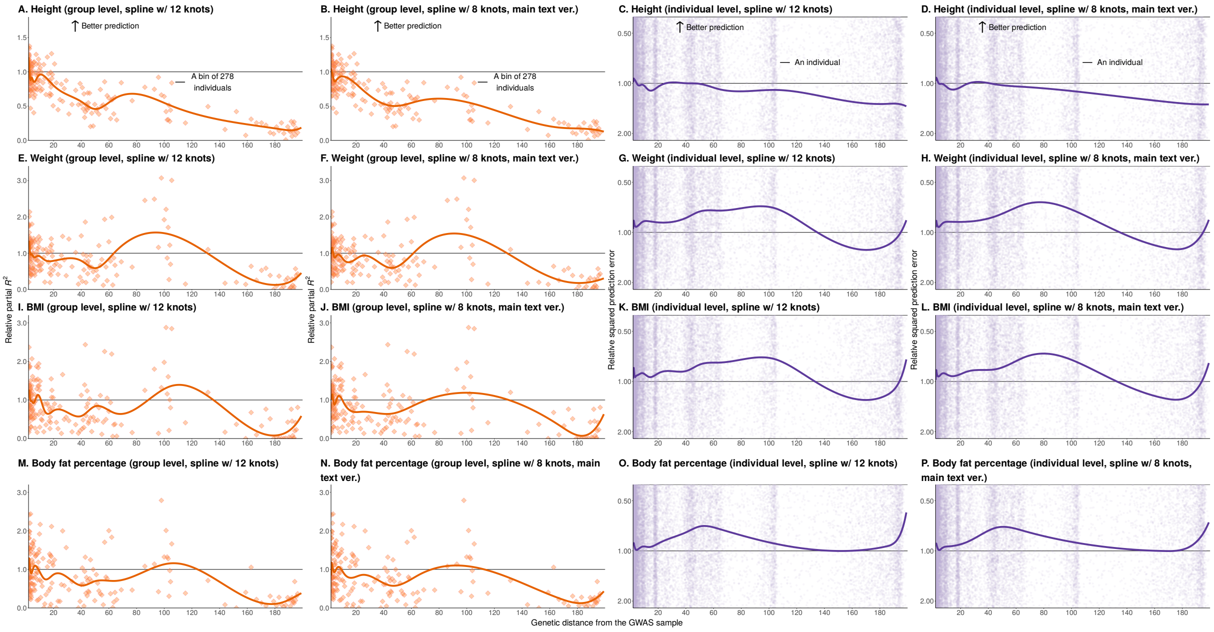

Supplementary Figure 46: Trends of portability, visualized using 12 knots in cubic line fits, for anthropometric measurements. This figure presents the same analysis as Fig. 2 and Supplementary Fig. 2, but the first and third columns (A, C, E, G, I, K, M, O) are plots with cubic splines of 12 knots. The second and fourth columns (B, D, F, H, J, L, N, P) contain plots with cubic splines of 8 knots, taken from the main text or supplementary text for comparison. For both knot numbers, the knots were placed based on the density of data points. At the group level (left two columns), we measured prediction accuracy with the squared partial correlation between the PGS and the trait value in 250 bins of 278 individuals each. At the individual level (right two columns), we measured prediction accuracy as the squared difference between the predicted phenotype and the true phenotype value. At both the group and individual levels, y-axis values show relative prediction accuracy, i.e. prediction accuracy divided by a baseline value. The baseline value is the mean prediction accuracy in 25 bins with median genetic distances that are closest to the mean genetic distance for GWAS individuals. Supplementary Table 1 details these baseline values for each trait. For all panels, no repeated measurements from the same individual were taken.

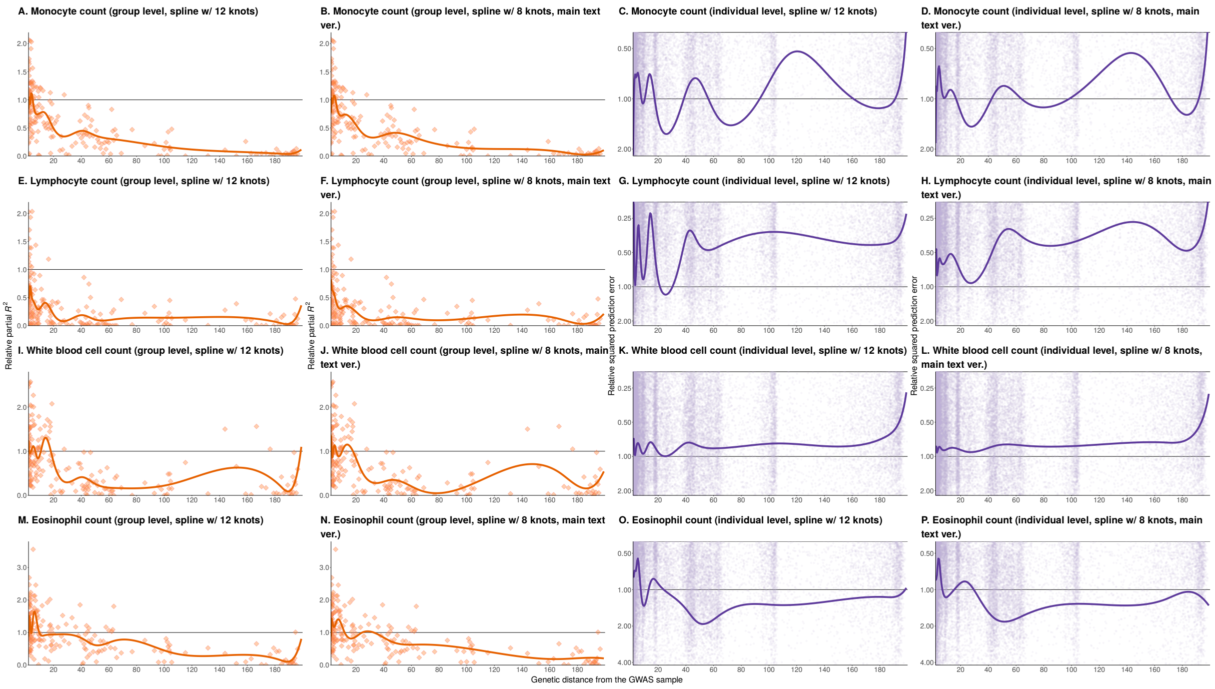

Supplementary Figure 47: Trends of portability, visualized using 12 knots in cubic line fits, for white blood cell-related traits. This figure presents the same analysis as Fig. 2 and Supplementary Fig. 3, but the first and third columns (A, C, E, G, I, K, M, O) are plots with cubic splines of 12 knots. The second and fourth columns (B, D, F, H, J, L, N, P) contain plots with cubic splines of 8 knots, taken from the main text or supplementary text for comparison. For both knot numbers, the knots were placed based on the density of data points. At the group level (left two columns), we measured prediction accuracy with the squared partial correlation between the PGS and the trait value in 250 bins of 278 individuals each. At the individual level (right two columns), we measured prediction accuracy as the squared difference between the predicted phenotype and the true phenotype value. At both the group and individual levels, y-axis values show relative prediction accuracy, i.e. prediction accuracy divided by a baseline value. The baseline value is the mean prediction accuracy in 25 bins with median genetic distances that are closest to the mean genetic distance for GWAS individuals. Supplementary Table 1 details these baseline values for each trait. For all panels, no repeated measurements from the same individual were taken.

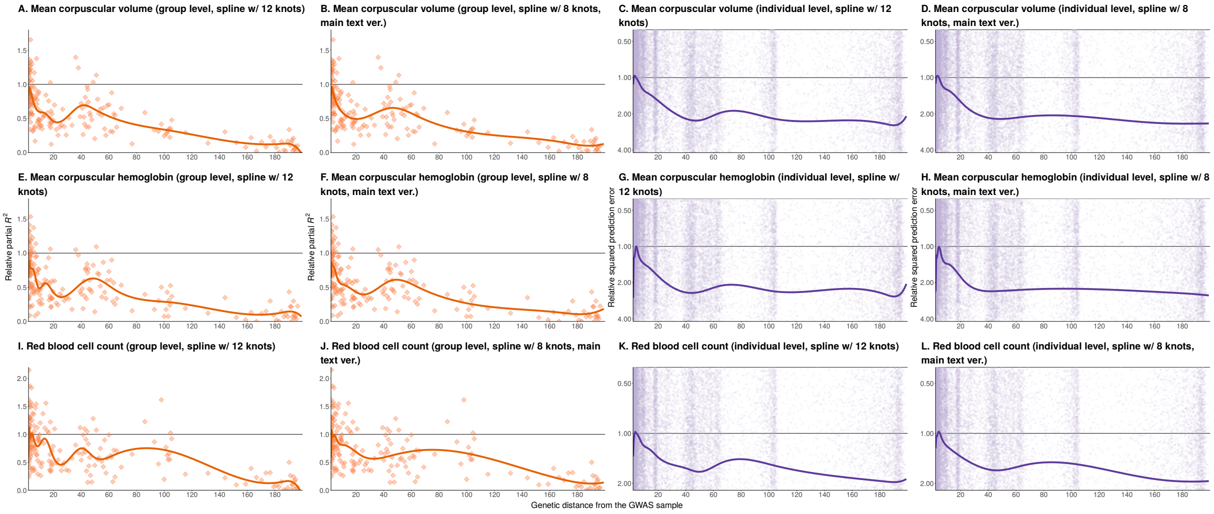

Supplementary Figure 48: Trends of portability, visualized using 12 knots in cubic line fits, for red blood cell-related traits. This figure presents the same analysis as Fig. 2 and Supplementary Fig. 4, but the first and third columns (A, C, E, G, I, K) are plots with cubic splines of 12 knots. The second and fourth columns (B, D, F, H, J, L) contain plots with cubic splines of 8 knots, taken from the main text or supplementary text for comparison. For both knot numbers, the knots were placed based on the density of data points. At the group level (left two columns), we measured prediction accuracy with the squared partial correlation between the PGS and the trait value in 250 bins of 278 individuals each. At the individual level (right two columns), we measured prediction accuracy as the squared difference between the predicted phenotype and the true phenotype value. At both the group and individual levels, y-axis values show relative prediction accuracy, i.e. prediction accuracy divided by a baseline value. The baseline value is the mean prediction accuracy in 25 bins with median genetic distances that are closest to the mean genetic distance for GWAS individuals. Supplementary Table 1 details these baseline values for each trait. For all panels, no repeated measurements from the same individual were taken.

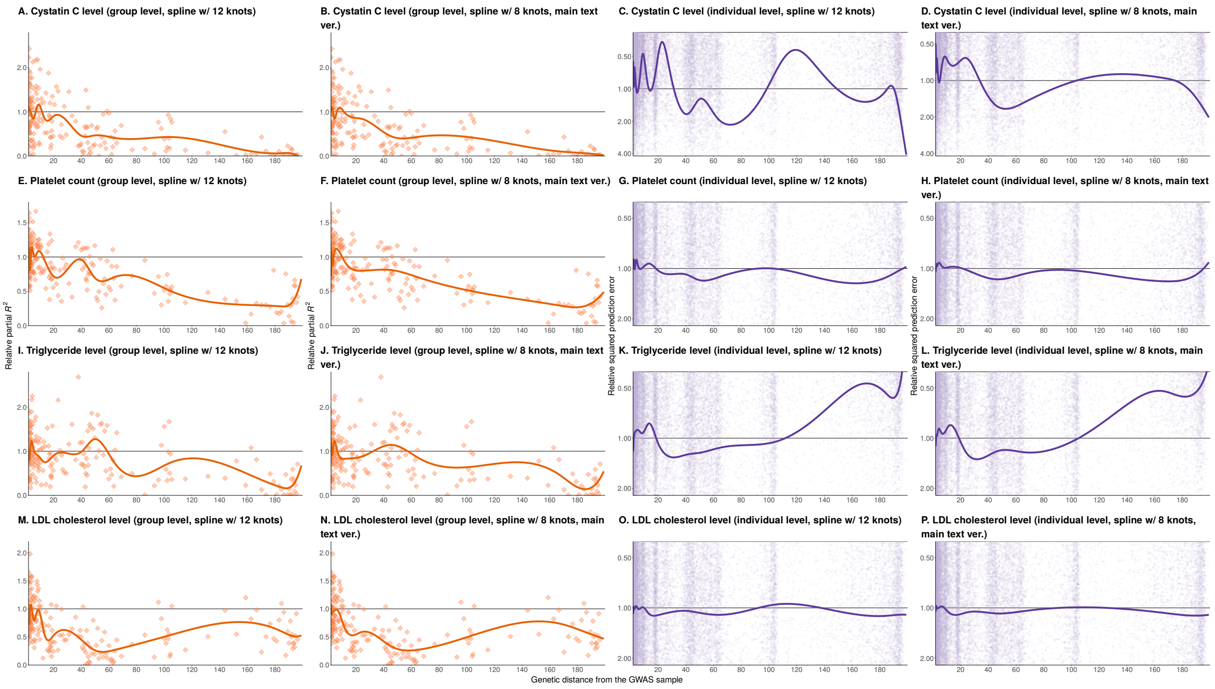

Supplementary Figure 49: Trends of portability, visualized using 12 knots in cubic line fits, for other biomarkers. This figure presents the same analysis as Fig. 2 and Supplementary Fig. 5, but the first and third columns (A, C, E, G, I, K, M, O) are plots with cubic splines of 12 knots. The second and fourth columns (B, D, F, H, J, L, N, P) contain plots with cubic splines of 8 knots, taken from the main text or supplementary text for comparison. For both knot numbers, the knots were placed based on the density of data points. At the group level (left two columns), we measured prediction accuracy with the squared partial correlation between the PGS and the trait value in 250 bins of 278 individuals each. At the individual level (right two columns), we measured prediction accuracy as the squared difference between the predicted phenotype and the true phenotype value. At both the group and individual levels, y-axis values show relative prediction accuracy, i.e. prediction accuracy divided by a baseline value. The baseline value is the mean prediction accuracy in 25 bins with median genetic distances that are closest to the mean genetic distance for GWAS individuals. Supplementary Table 1 details these baseline values for each trait. For all panels, no repeated measurements from the same individual were taken.

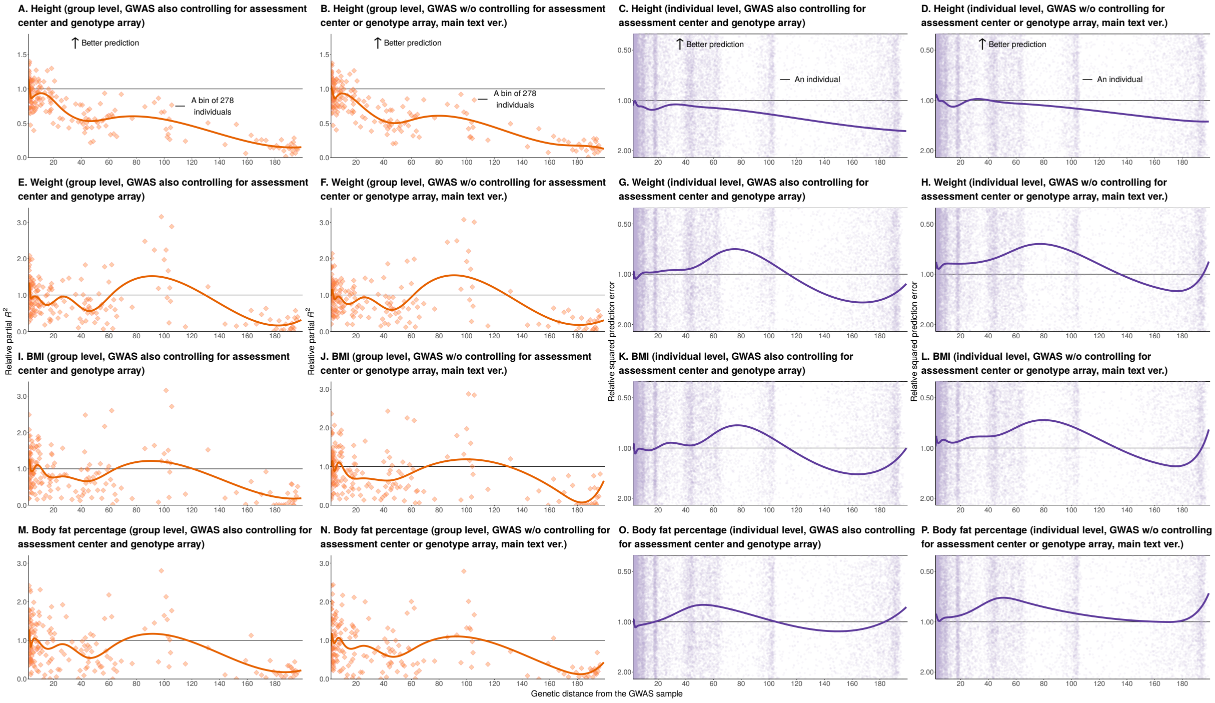

Supplementary Figure 50: Trends of portability, when including additional GWAS covariates, for anthropometric measurements. This figure presents the same analysis as Fig. 2 and Supplementary Fig. 2, but the first and third columns (A, C, E, G, I, K, M, O) are plots controlling for assessment center and genotype array in the GWAS. The second and fourth columns (B, D, F, H, J, L, N, P) contain plots without controlling for assessment center and genotype array in the GWAS, taken from the main text or supplementary text for comparison. At the group level (left two columns), we measured prediction accuracy with the squared partial correlation between the PGS and the trait value in 250 bins of 278 individuals each. At the individual level (right two columns), we measured prediction accuracy as the squared difference between the predicted phenotype and the true phenotype value. At both the group and individual levels, y-axis values show relative prediction accuracy, i.e. prediction accuracy divided by a baseline value. The baseline value is the mean prediction accuracy in 25 bins with median genetic distances that are closest to the mean genetic distance for GWAS individuals. Supplementary Table 1 details these baseline values for each trait. Curves show cubic spline fits, with 8 knots placed based on the density of data points. For all panels, no repeated measurements from the same individual were taken.

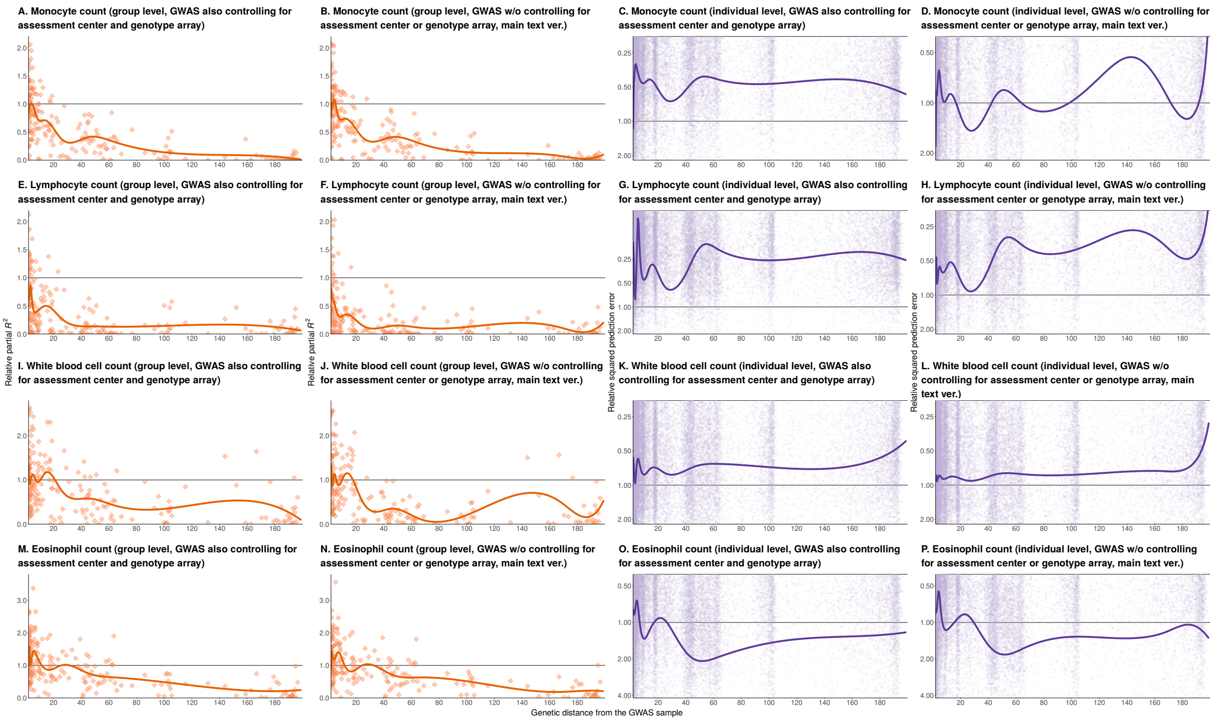

Supplementary Figure 51: Trends of portability, when including additional GWAS covariates, for white blood cell-related traits. This figure presents the same analysis as Fig. 2 and Supplementary Fig. 3, but the first and third columns (A, C, E, G, I, K, M, O) are plots controlling for assessment center and genotype array in the GWAS. The second and fourth columns (B, D, F, H, J, L, N, P) contain plots without controlling for assessment center and genotype array in the GWAS, taken from the main text or supplementary text for comparison. At the group level (left two columns), we measured prediction accuracy with the squared partial correlation between the PGS and the trait value in 250 bins of 278 individuals each. At the individual level (right two columns), we measured prediction accuracy as the squared difference between the predicted phenotype and the true phenotype value. At both the group and individual levels, y-axis values show relative prediction accuracy, i.e. prediction accuracy divided by a baseline value. The baseline value is the mean prediction accuracy in 25 bins with median genetic distances that are closest to the mean genetic distance for GWAS individuals. Supplementary Table 1 details these baseline values for each trait. Curves show cubic spline fits, with 8 knots placed based on the density of data points. For all panels, no repeated measurements from the same individual were taken.

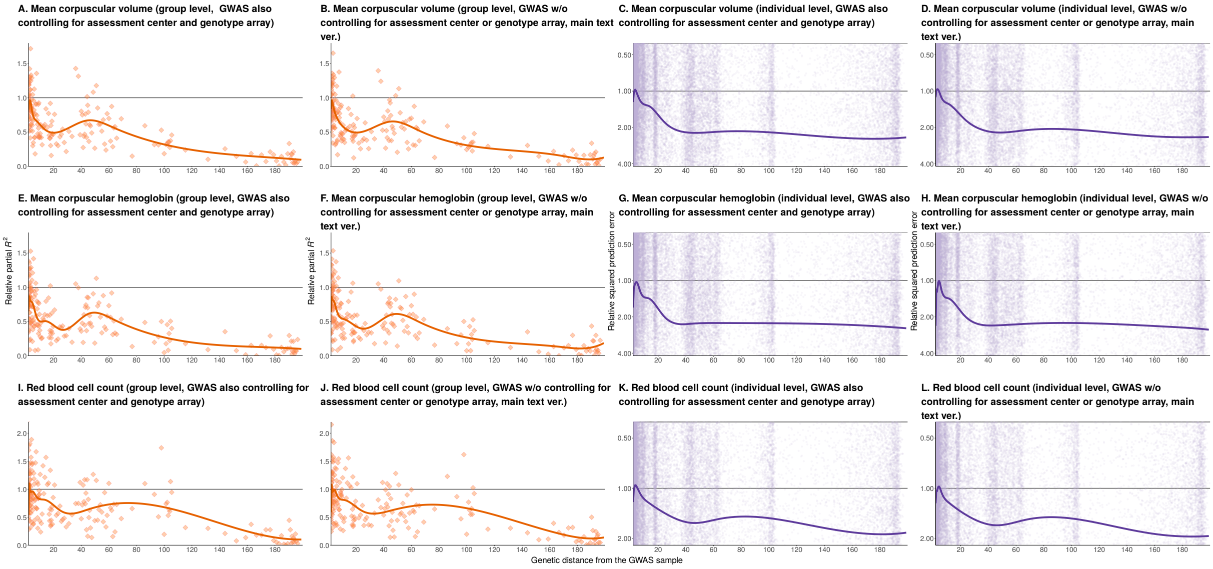

Supplementary Figure 52: Trends of portability, when including additional GWAS covariates, for red blood cell-related traits. This figure presents the same analysis as Fig. 2 and Supplementary Fig. 4, but the first and third columns (A, C, E, G, I, K, M, O) are plots controlling for assessment center and genotype array in the GWAS. The second and fourth columns (B, D, F, H, J, L, N, P) contain plots without controlling for assessment center and genotype array in the GWAS, taken from the main text or supplementary text for comparison. At the group level (left two columns), we measured prediction accuracy with the squared partial correlation between the PGS and the trait value in 250 bins of 278 individuals each. At the individual level (right two columns), we measured prediction accuracy as the squared difference between the predicted phenotype and the true phenotype value. At both the group and individual levels, y-axis values show relative prediction accuracy, i.e. prediction accuracy divided by a baseline value. The baseline value is the mean prediction accuracy in 25 bins with median genetic distances that are closest to the mean genetic distance for GWAS individuals. Supplementary Table 1 details these baseline values for each trait. Curves show cubic spline fits, with 8 knots placed based on the density of data points. For all panels, no repeated measurements from the same individual were taken.

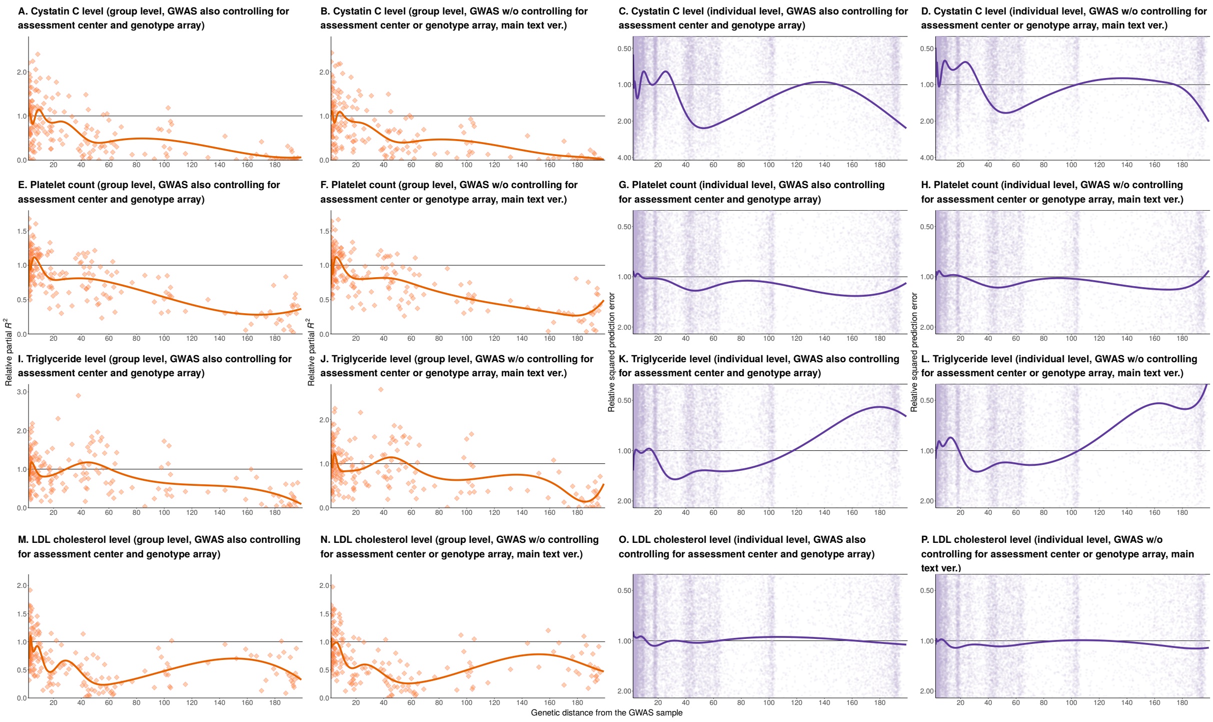

Supplementary Figure 53: Trends of portability, when including additional GWAS covariates, for other biomarkers. This figure presents the same analysis as Fig. 2 and Supplementary Fig. 5, but the first and third columns (A, C, E, G, I, K, M, O) are plots controlling for assessment center and genotype array in the GWAS. The second and fourth columns (B, D, F, H, J, L, N, P) contain plots without controlling for assessment center and genotype array in the GWAS, taken from the main text or supplementary text for comparison. At the group level (left two columns), we measured prediction accuracy with the squared partial correlation between the PGS and the trait value in 250 bins of 278 individuals each. At the individual level (right two columns), we measured prediction accuracy as the squared difference between the predicted phenotype and the true phenotype value. At both the group and individual levels, y-axis values show relative prediction accuracy, i.e. prediction accuracy divided by a baseline value. The baseline value is the mean prediction accuracy in 25 bins with median genetic distances that are closest to the mean genetic distance for GWAS individuals. Supplementary Table 1 details these baseline values for each trait. Curves show cubic spline fits, with 8 knots placed based on the density of data points. For all panels, no repeated measurements from the same individual were taken.

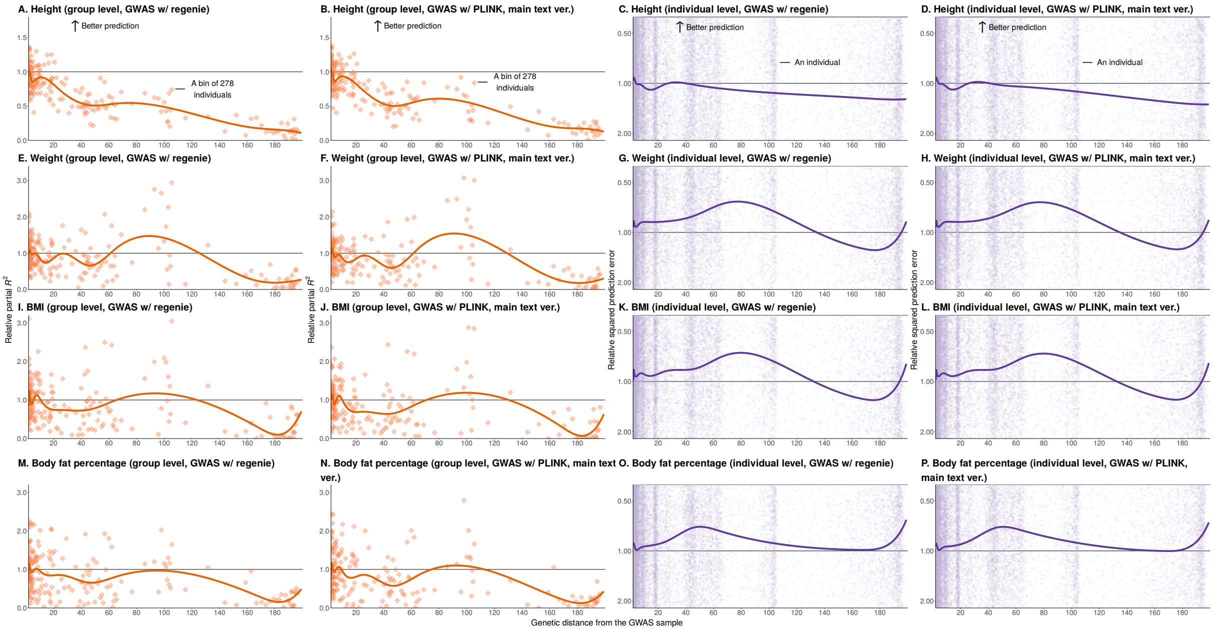

Supplementary Figure 54: Trends of portability, with GWAS run using regenie, for anthropometric measurements. This figure presents the same analysis as Fig. 2 and Supplementary Fig. 2, but the first and third columns (A, C, E, G, I, K, M, O) are plots with GWAS run with regenie[2]. The second and fourth columns (B, D, F, H, J, L, N, P) contain plots with GWAS run with PLINK[11], taken from the main text or supplementary text for comparison. At the group level (left two columns), we measured prediction accuracy with the squared partial correlation between the PGS and the trait value in 250 bins of 278 individuals each. At the individual level (right two columns), we measured prediction accuracy as the squared difference between the predicted phenotype and the true phenotype value. At both the group and individual levels, y-axis values show relative prediction accuracy, i.e. prediction accuracy divided by a baseline value. The baseline value is the mean prediction accuracy in 25 bins with median genetic distances that are closest to the mean genetic distance for GWAS individuals. Supplementary Table 1 details these baseline values for each trait. Curves show cubic spline fits, with 8 knots placed based on the density of data points. For all panels, no repeated measurements from the same individual were taken.

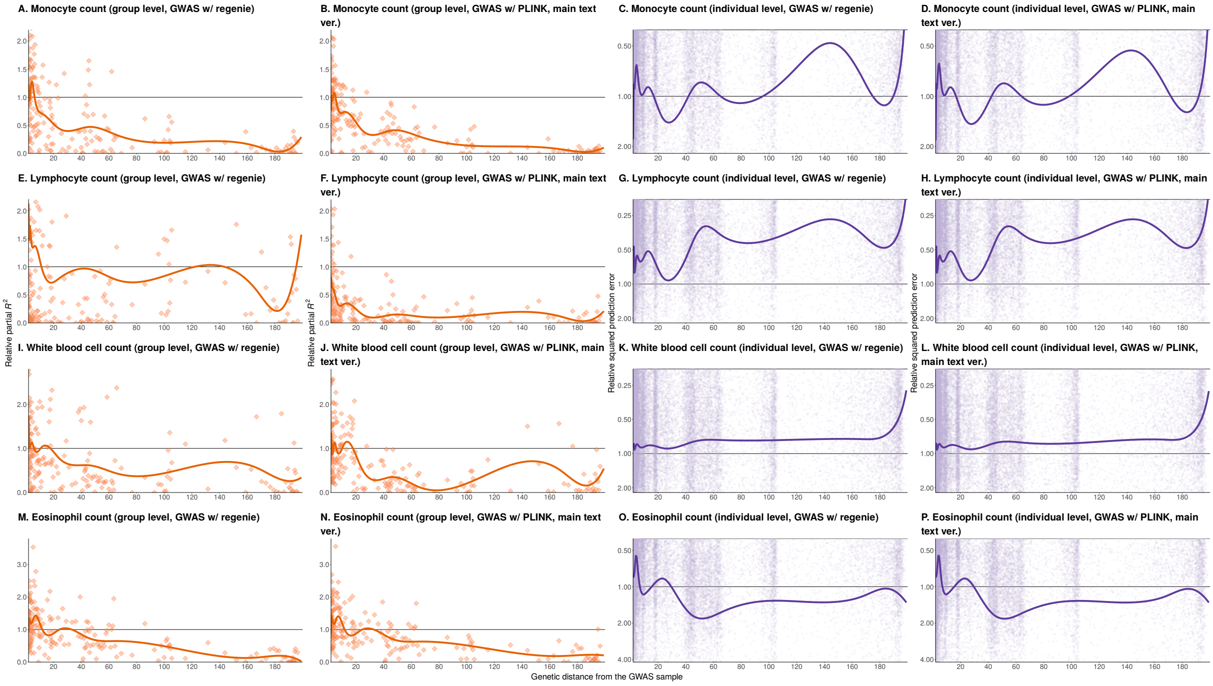

Supplementary Figure 55: Trends of portability, with GWAS run using regenie, for white blood cell-related traits. This figure presents the same analysis as Fig. 2 and Supplementary Fig. 3, but the first and third columns (A, C, E, G, I, K, M, O) are plots with GWAS run with regenie[2]. The second and fourth columns (B, D, F, H, J, L, N, P) contain plots with GWAS run with PLINK[11], taken from the main text or supplementary text for comparison. At the group level (left two columns), we measured prediction accuracy with the squared partial correlation between the PGS and the trait value in 250 bins of 278 individuals each. At the individual level (right two columns), we measured prediction accuracy as the squared difference between the predicted phenotype and the true phenotype value. At both the group and individual levels, y-axis values show relative prediction accuracy, i.e. prediction accuracy divided by a baseline value. The baseline value is the mean prediction accuracy in 25 bins with median genetic distances that are closest to the mean genetic distance for GWAS individuals. Supplementary Table 1 details these baseline values for each trait. Curves show cubic spline fits, with 8 knots placed based on the density of data points. For all panels, no repeated measurements from the same individual were taken.

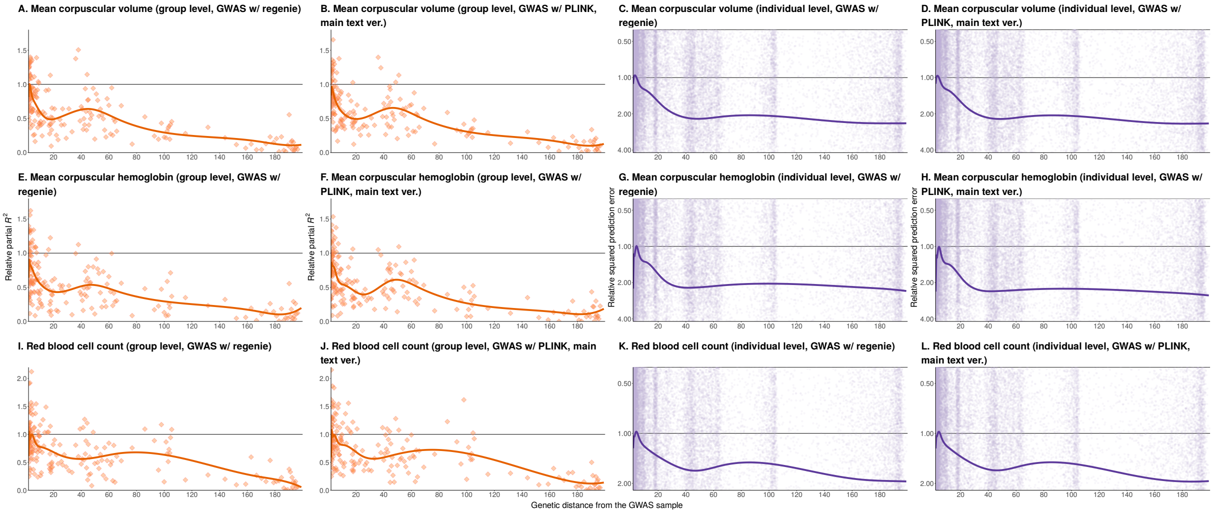

Supplementary Figure 56: Trends of portability, with GWAS run using regenie, for red blood cell-related traits. This figure presents the same analysis as Fig. 2 and Supplementary Fig. 4, but the first and third columns (A, C, E, G, I, K, M, O) are plots with GWAS run with regenie[2]. The second and fourth columns (B, D, F, H, J, L, N, P) contain plots with GWAS run with PLINK[11], taken from the main text or supplementary text for comparison. At the group level (left two columns), we measured prediction accuracy with the squared partial correlation between the PGS and the trait value in 250 bins of 278 individuals each. At the individual level (right two columns), we measured prediction accuracy as the squared difference between the predicted phenotype and the true phenotype value. At both the group and individual levels, y-axis values show relative prediction accuracy, i.e. prediction accuracy divided by a baseline value. The baseline value is the mean prediction accuracy in 25 bins with median genetic distances that are closest to the mean genetic distance for GWAS individuals. Supplementary Table 1 details these baseline values for each trait. Curves show cubic spline fits, with 8 knots placed based on the density of data points. For all panels, no repeated measurements from the same individual were taken.

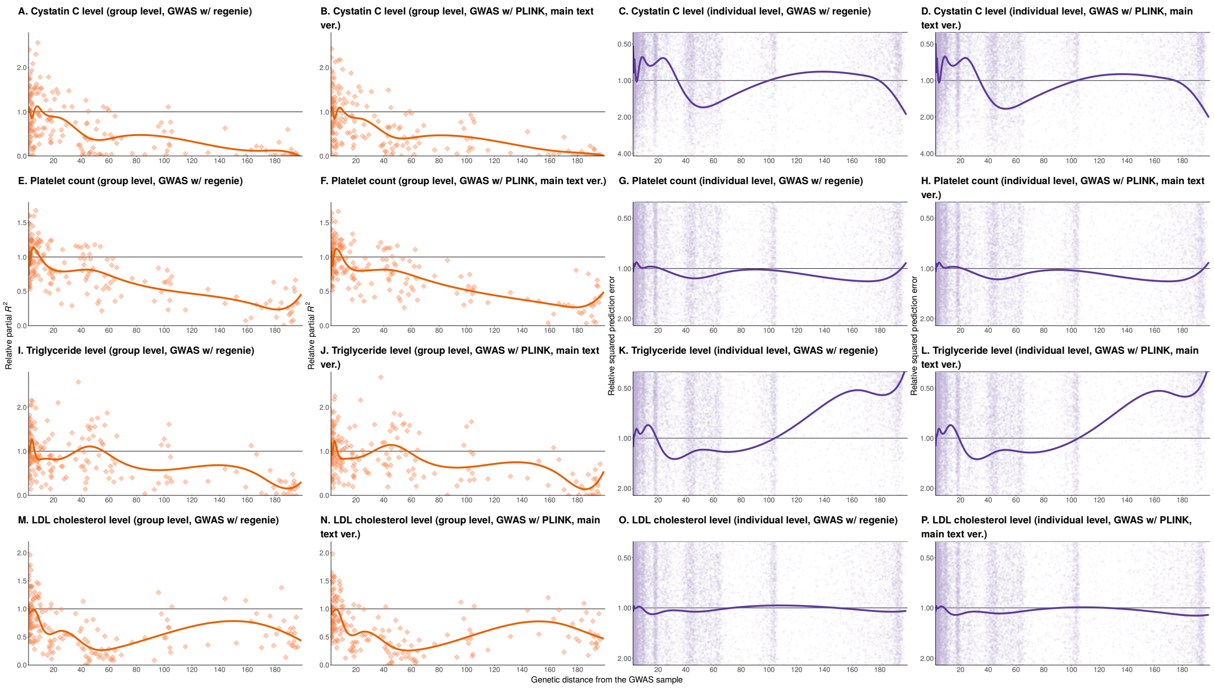

Supplementary Figure 57: Trends of portability with GWAS run using regenie, for other biomarkers. This figure presents the same analysis as Fig. 2 and Supplementary Fig. 5, but the first and third columns (A, C, E, G, I, K, M, O) are plots with GWAS run with regenie[2]. The second and fourth columns (B, D, F, H, J, L, N, P) contain plots with GWAS run with PLINK[11], taken from the main text or supplementary text for comparison. At the group level (left two columns), we measured prediction accuracy with the squared partial correlation between the PGS and the trait value in 250 bins of 278 individuals each. At the individual level (right two columns), we measured prediction accuracy as the squared difference between the predicted phenotype and the true phenotype value. At both the group and individual levels, y-axis values show relative prediction accuracy, i.e. prediction accuracy divided by a baseline value. The baseline value is the mean prediction accuracy in 25 bins with median genetic distances that are closest to the mean genetic distance for GWAS individuals. Supplementary Table 1 details these baseline values for each trait. Curves show cubic spline fits, with 8 knots placed based on the density of data points. For all panels, no repeated measurements from the same individual were taken.

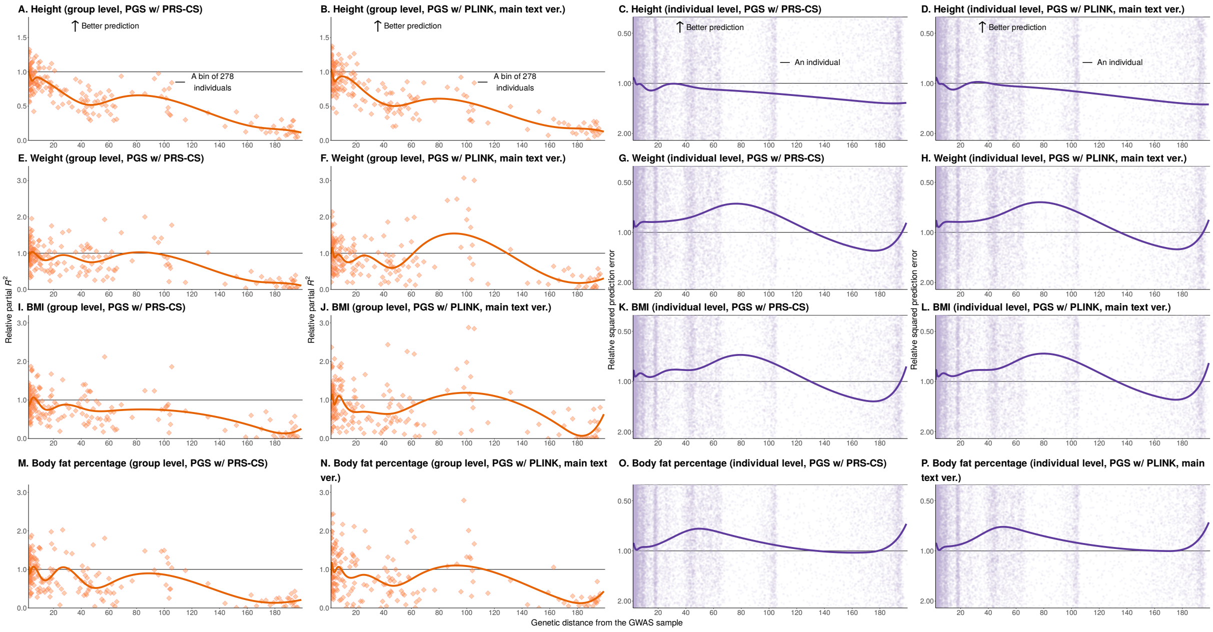

Supplementary Figure 58: Trends of portability, with PGS constructed using PRS-CS, for anthropometric measurements. This figure presents the same analysis as Fig. 2 and Supplementary Fig. 2, but the first and third columns (A, C, E, G, I, K, M, O) are plots with PRS reconstructed with PRS-CS[3]. The second and fourth columns (B, D, F, H, J, L, N, P) contain plots with PGS constructed with PLINK[11], taken from the main text or supplementary text for comparison. At the group level (left two columns), we measured prediction accuracy with the squared partial correlation between the PGS and the trait value in 250 bins of 278 individuals each. At the individual level (right two columns), we measured prediction accuracy as the squared difference between the predicted phenotype and the true phenotype value. At both the group and individual levels, y-axis values show relative prediction accuracy, i.e. prediction accuracy divided by a baseline value. The baseline value is the mean prediction accuracy in 25 bins with median genetic distances that are closest to the mean genetic distance for GWAS individuals. Supplementary Table 1 details these baseline values for each trait. Curves show cubic spline fits, with 8 knots placed based on the density of data points. For all panels, no repeated measurements from the same individual were taken.

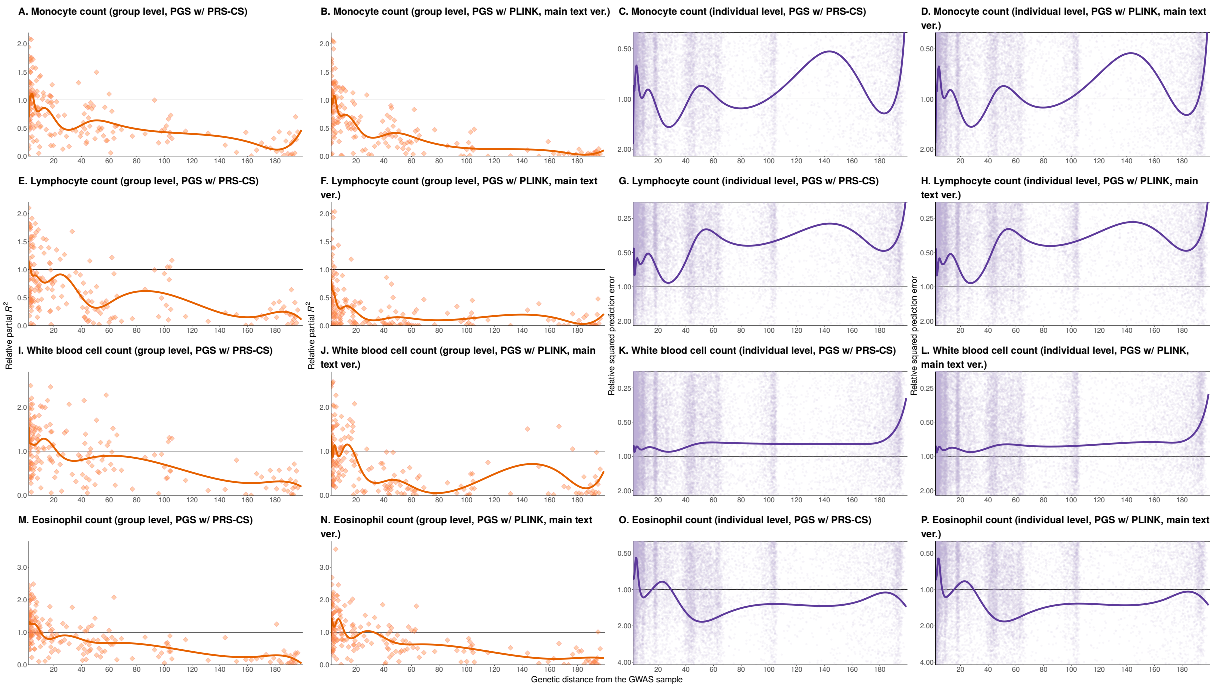

Supplementary Figure 59: Trends of portability, with PGS constructed using PRS-CS, for white blood cell-related traits. This figure presents the same analysis as Fig. 2 and Supplementary Fig. 3, but the first and third columns (A, C, E, G, I, K, M, O) are plots with PRS reconstructed with PRS-CS [3]. The second and fourth columns (B, D, F, H, J, L, N, P) contain plots with PGS constructed with PLINK [11], taken from the main text or supplementary text for comparison. At the group level (left two columns), we measured prediction accuracy with the squared partial correlation between the PGS and the trait value in 250 bins of 278 individuals each. At the individual level (right two columns), we measured prediction accuracy as the squared difference between the predicted phenotype and the true phenotype value. At both the group and individual levels, y-axis values show relative prediction accuracy, i.e. prediction accuracy divided by a baseline value. The baseline value is the mean prediction accuracy in 25 bins with median genetic distances that are closest to the mean genetic distance for GWAS individuals. Supplementary Table 1 details these baseline values for each trait. Curves show cubic spline fits, with 8 knots placed based on the density of data points. For all panels, no repeated measurements from the same individual were taken.

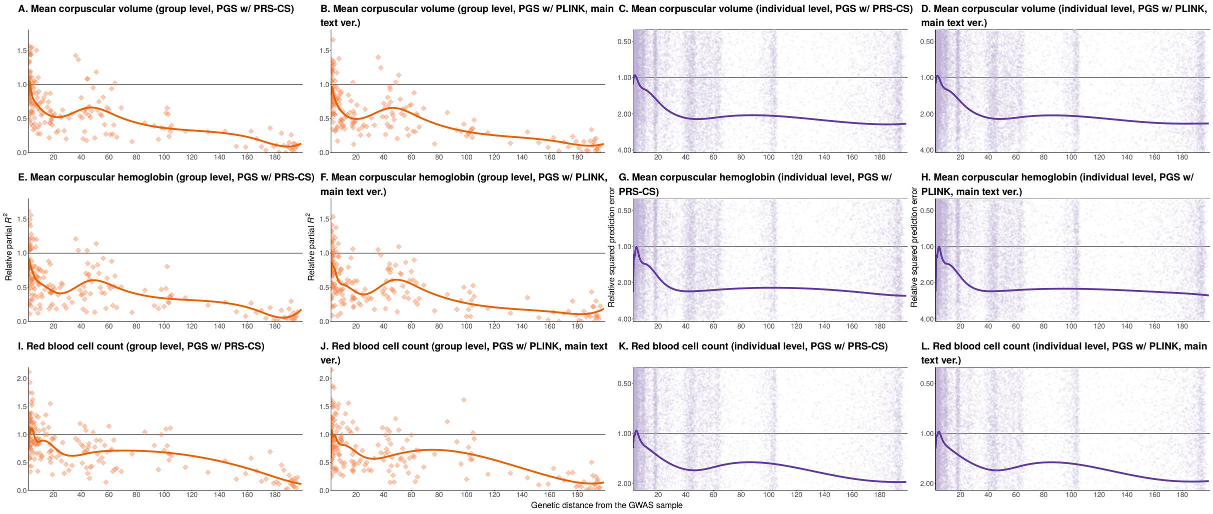

Supplementary Figure 60: Trends of portability, with PGS constructed using PRS-CS, for red blood cell-related traits. This figure presents the same analysis as Fig. 2 and Supplementary Fig. 4, but the first and third columns (A, C, E, G, I, K, M, O) are plots with PRS reconstructed with PRS-CS[3]. The second and fourth columns (B, D, F, H, J, L, N, P) contain plots with PGS constructed with PLINK[11], taken from the main text or supplementary text for comparison. At the group level (left two columns), we measured prediction accuracy with the squared partial correlation between the PGS and the trait value in 250 bins of 278 individuals each. At the individual level (right two columns), we measured prediction accuracy as the squared difference between the predicted phenotype and the true phenotype value. At both the group and individual levels, y-axis values show relative prediction accuracy, i.e. prediction accuracy divided by a baseline value. The baseline value is the mean prediction accuracy in 25 bins with median genetic distances that are closest to the mean genetic distance for GWAS individuals. Supplementary Table 1 details these baseline values for each trait. Curves show cubic spline fits, with 8 knots placed based on the density of data points. For all panels, no repeated measurements from the same individual were taken.

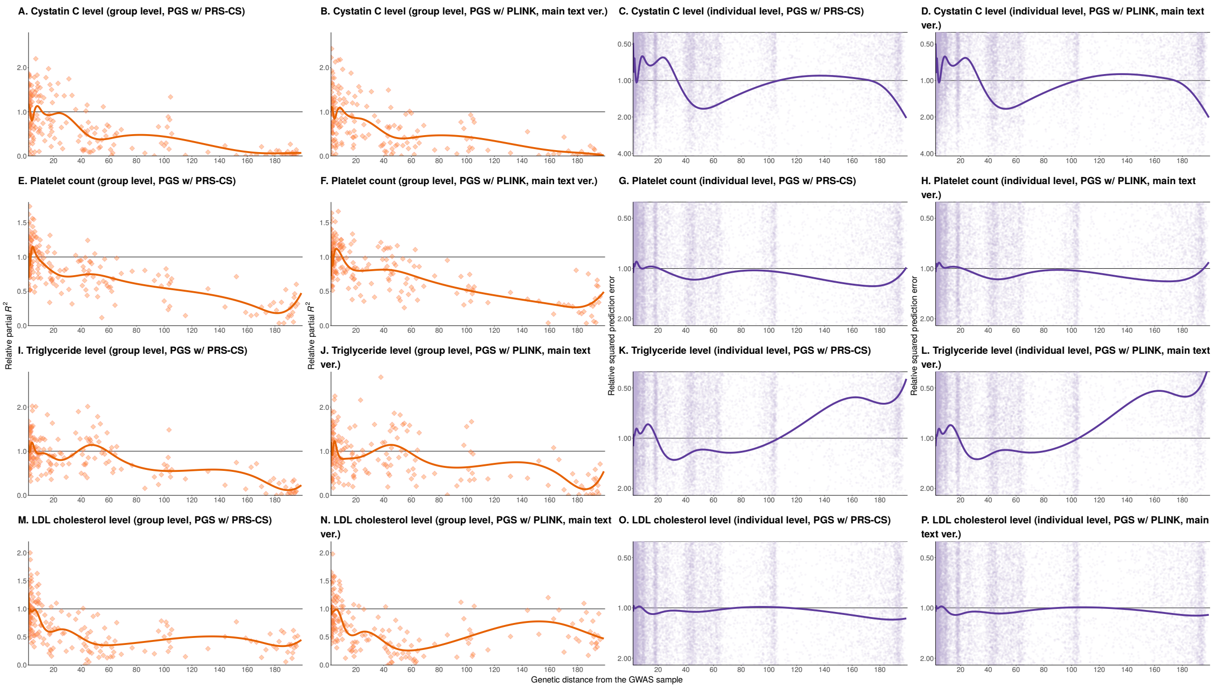

Supplementary Figure 61: Trends of portability, with PGS constructed using PRS-CS, for other biomarkers. This figure presents the same analysis as Fig. 2 and Supplementary Fig. 5, but the first and third columns (A, C, E, G, I, K, M, O) are plots with PRS reconstructed with PRS-CS[3]. The second and fourth columns (B, D, F, H, J, L, N, P) contain plots with PGS constructed with PLINK[11], taken from the main text or supplementary text for comparison. At the group level (left two columns), we measured prediction accuracy with the squared partial correlation between the PGS and the trait value in 250 bins of 278 individuals each. At the individual level (right two columns), we measured prediction accuracy as the squared difference between the predicted phenotype and the true phenotype value. At both the group and individual levels, y-axis values show relative prediction accuracy, i.e. prediction accuracy divided by a baseline value. The baseline value is the mean prediction accuracy in 25 bins with median genetic distances that are closest to the mean genetic distance for GWAS individuals. Supplementary Table 1 details these baseline values for each trait. Curves show cubic spline fits, with 8 knots placed based on the density of data points. For all panels, no repeated measurements from the same individual were taken.

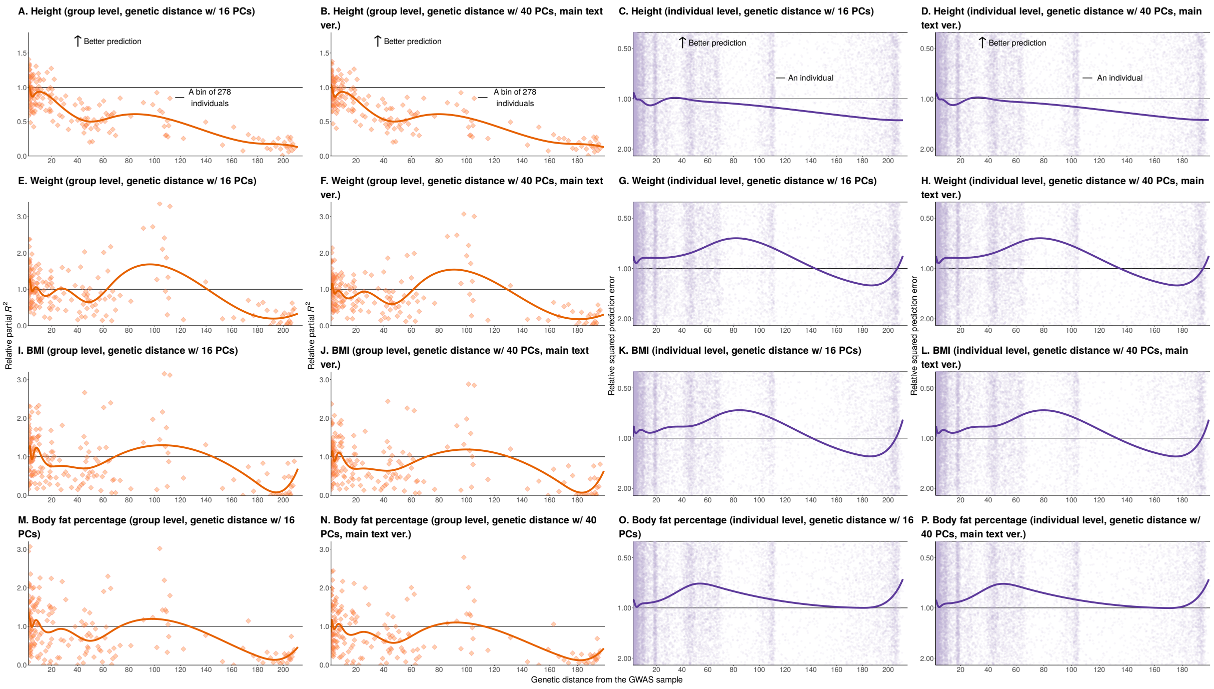

Supplementary Figure 62: A comparison of portability trends, with genetic distance defined in two different ways, for anthropometric measurements. This figure presents the same analysis as Fig. 2 and Supplementary Fig. 2, but the first and third columns (A, C, E, G, I, K, M, O) show results obtained with a genetic distance metric defined using the first 16 PCs alone. The second and fourth columns (B, D, F, H, J, L, N, P) show results obtained with the measure of genetic distances that uses 40 PCs, as in the main text. At the group level (left two columns), we measured prediction accuracy with the squared partial correlation between the PGS and the trait value in 250 bins of 278 individuals each. At the individual level (right two columns), we measured prediction accuracy as the squared difference between the predicted phenotype and the true phenotype value. At both the group and individual levels, y-axis values show relative prediction accuracy, i.e. prediction accuracy divided by a baseline value. The baseline value is the mean prediction accuracy in 25 bins with median genetic distances that are closest to the mean genetic distance for GWAS individuals. Supplementary Table 1 details these baseline values for each trait. Curves show cubic spline fits, with 8 knots placed based on the density of data points. For all panels, no repeated measurements from the same individual were taken.

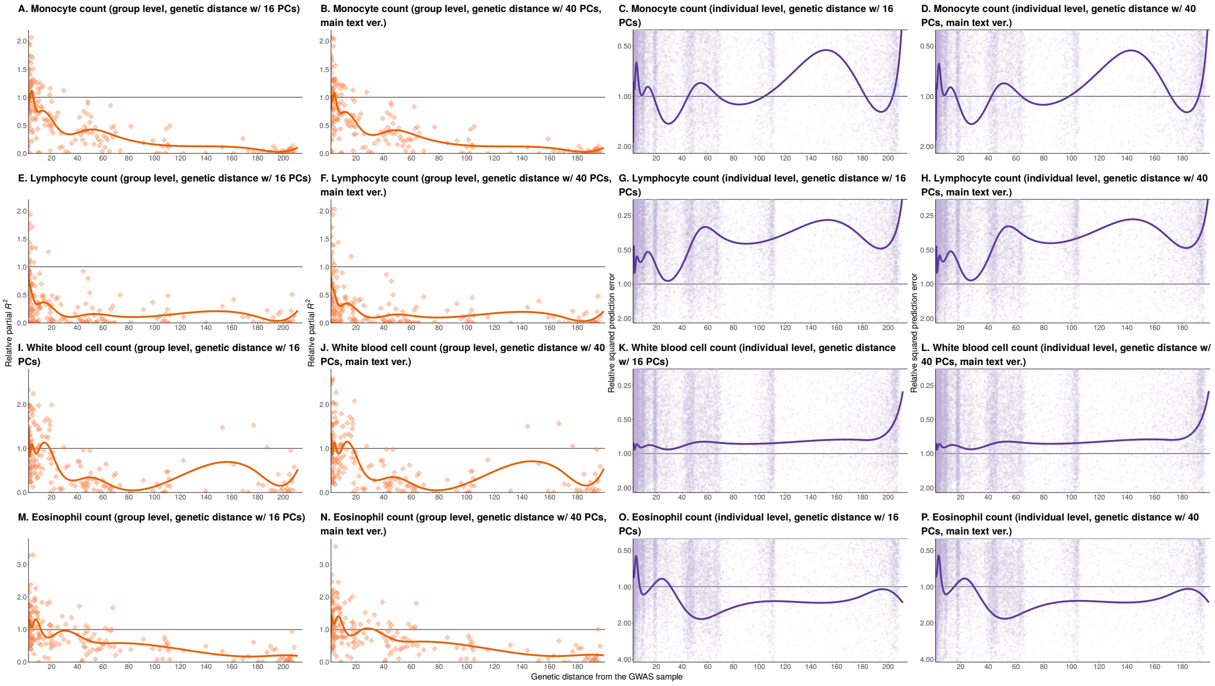

Supplementary Figure 63: A comparison of portability trends, with genetic distance defined in two different ways, for white blood cell-related traits. This figure presents the same analysis as Fig. 2 and Supplementary Fig. 3, but the first and third columns (A, C, E, G, I, K, M, O) show results obtained with a genetic distance metric defined using the first 16 PCs alone. The second and fourth columns (B, D, F, H, J, L, N, P) show results obtained with the measure of genetic distances that uses 40 PCs, as in the main text. At the group level (left two columns), we measured prediction accuracy with the squared partial correlation between the PGS and the trait value in 250 bins of 278 individuals each. At the individual level (right two columns), we measured prediction accuracy as the squared difference between the predicted phenotype and the true phenotype value. At both the group and individual levels, y-axis values show relative prediction accuracy, i.e. prediction accuracy divided by a baseline value. The baseline value is the mean prediction accuracy in 25 bins with median genetic distances that are closest to the mean genetic distance for GWAS individuals. Supplementary Table 1 details these baseline values for each trait. Curves show cubic spline fits, with 8 knots placed based on the density of data points. For all panels, no repeated measurements from the same individual were taken.

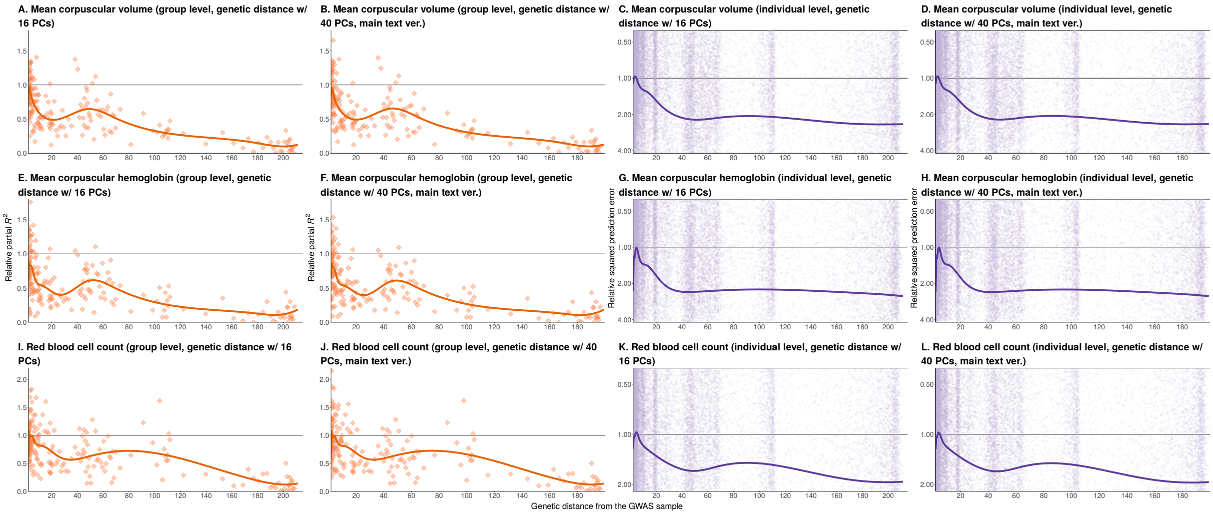

Supplementary Figure 64: A comparison of portability trends, with genetic distance defined in two different ways, for red blood cell-related traits. This figure presents the same analysis as Fig. 2 and Supplementary Fig. 4, but the first and third columns (A, C, E, G, I, K) show results obtained with a genetic distance metric defined using the first 16 PCs alone. The second and fourth columns (B, D, F, H, J, L) show results obtained with the measure of genetic distances that uses 40 PCs, as in the main text. At the group level (left two columns), we measured prediction accuracy with the squared partial correlation between the PGS and the trait value in 250 bins of 278 individuals each. At the individual level (right two columns), we measured prediction accuracy as the squared difference between the predicted phenotype and the true phenotype value. At both the group and individual levels, y-axis values show relative prediction accuracy, i.e. prediction accuracy divided by a baseline value. The baseline value is the mean prediction accuracy in 25 bins with median genetic distances that are closest to the mean genetic distance for GWAS individuals. Supplementary Table 1 details these baseline values for each trait. Curves show cubic spline fits, with 8 knots placed based on the density of data points. For all panels, no repeated measurements from the same individual were taken.

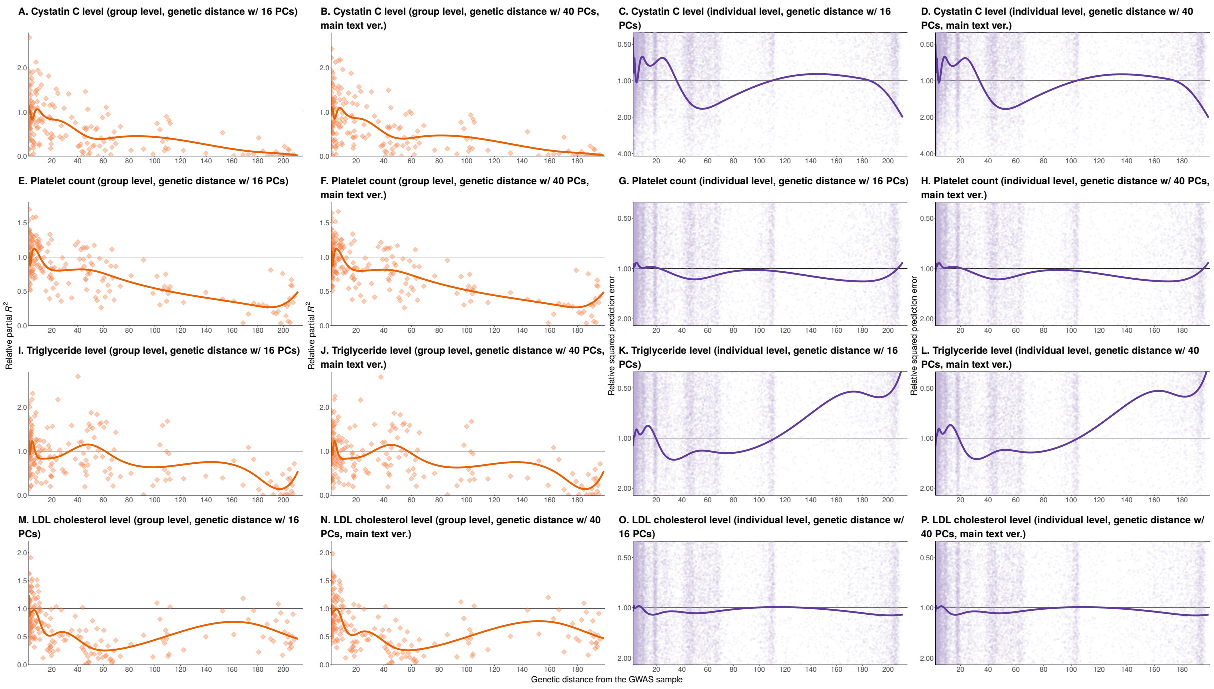

Supplementary Figure 65: A comparison of portability trends, with genetic distance defined in two different ways, for other biomarkers. This figure presents the same analysis as Fig. 2 and Supplementary Fig. 5, but the first and third columns (A, C, E, G, I, K, M, O) show results obtained with a genetic distance metric defined using the first 16 PCs alone. The second and fourth columns (B, D, F, H, J, L, N, P) show results obtained with the measure of genetic distances that uses 40 PCs, as in the main text. At the group level (left two columns), we measured prediction accuracy with the squared partial correlation between the PGS and the trait value in 250 bins of 278 individuals each. At the individual level (right two columns), we measured prediction accuracy as the squared difference between the predicted phenotype and the true phenotype value. At both the group and individual levels, y-axis values show relative prediction accuracy, i.e. prediction accuracy divided by a baseline value. The baseline value is the mean prediction accuracy in 25 bins with median genetic distances that are closest to the mean genetic distance for GWAS individuals. Supplementary Table 1 details these baseline values for each trait. Curves show cubic spline fits, with 8 knots placed based on the density of data points. For all panels, no repeated measurements from the same individual were taken.

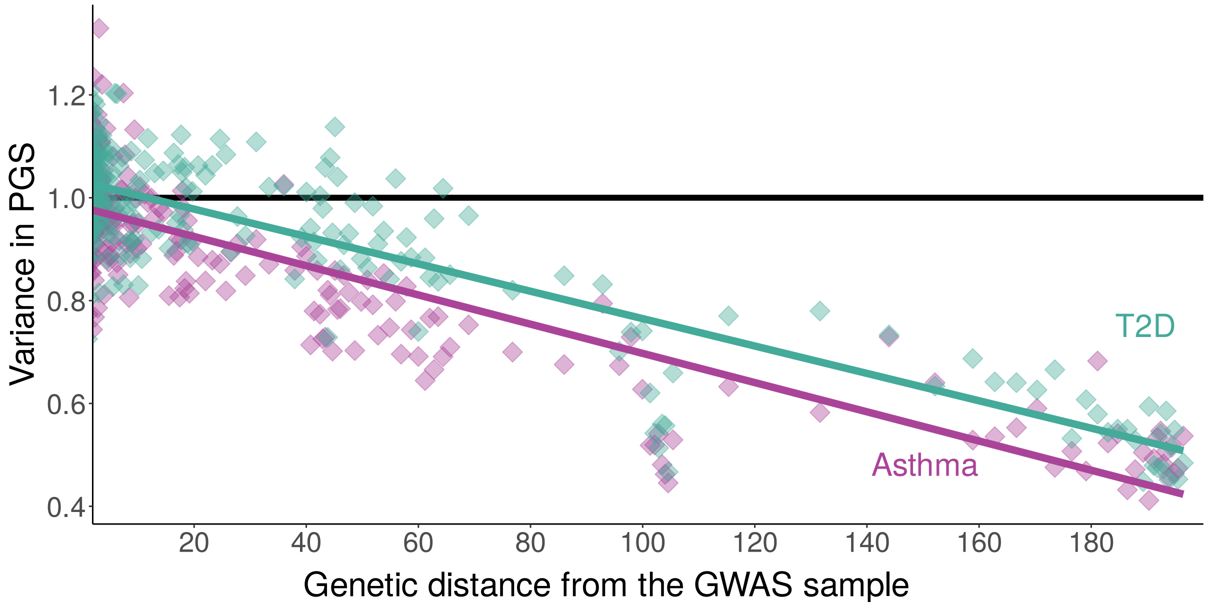

Supplementary Figure 66: Variance in disease PGS as a function of genetic distance. We binned the prediction individuals into 250 bins of 278 individuals each by genetic distance. Points show the PGS variance for each genetic distance bins. Lines show the ordinary least squares linear fit to the respective bin-level data. No repeated measurements from the same individual were taken.

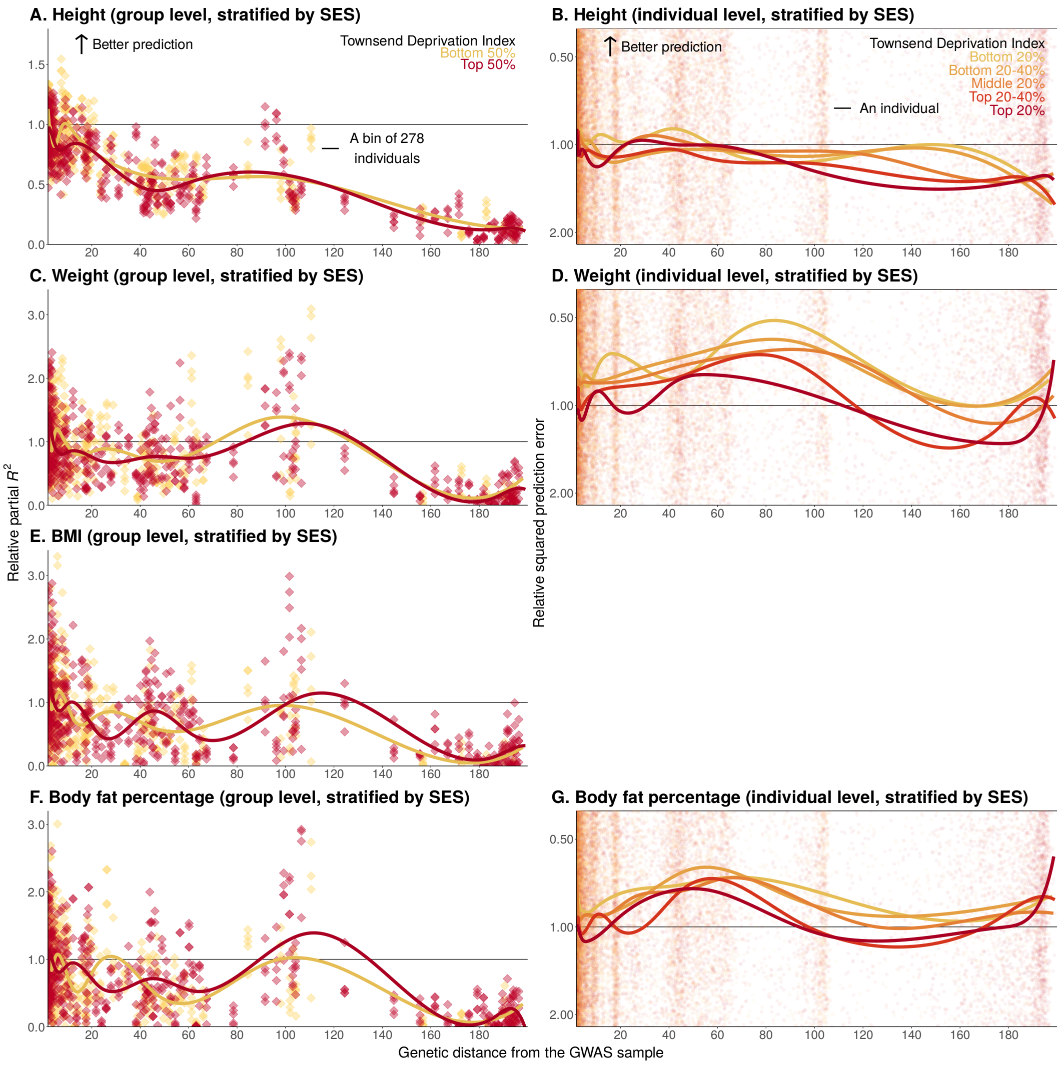

Supplementary Figure 67: Portability, as a function of both genetic distance and Townsend Deprivation Index, for anthropometric measurements. We first stratified the prediction sample individuals into equal-sized strata of Townsend Deprivation Index: 2 strata at the group level, shown on the left, and 5 equal-sized strata at the individual level, shown on the right panels. Within each stratum, we then performed the same analysis of prediction accuracy as a function of genetic distance as in Fig. 2 and Supplementary Fig. 2. At the group level, we measured prediction accuracy with the squared partial correlation between the PGS and the trait value in 250 bins of 278 individuals each. At the individual level, we measured prediction accuracy as the squared difference between the predicted phenotype and the true phenotype value. At both the group and individual levels, y-axis values show relative prediction accuracy, i.e. prediction accuracy divided by a baseline value. The baseline value is the mean prediction accuracy in 25 bins with median genetic distances that are closest to the mean genetic distance for GWAS individuals. Curves show cubic spline fits, with 8 knots placed based on the density of data points. Top represents those who are more deprived. See Fig. 3C for the individual level of BMI. For all panels, no repeated measurements from the same individual were taken.

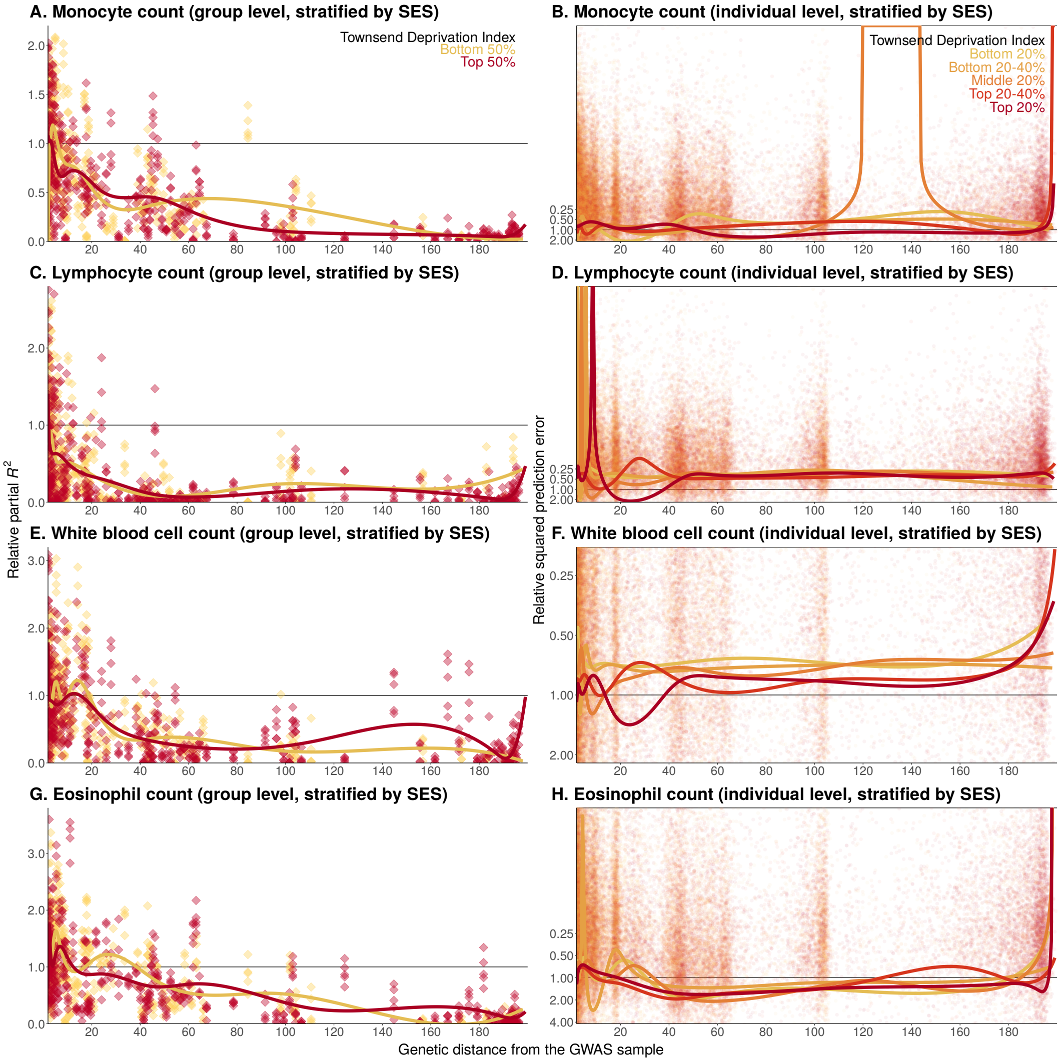

Supplementary Figure 68: Portability, as a function of both genetic distance and Townsend Deprivation Index, for white blood cell-related traits. We first stratified the prediction sample individuals into equal-sized strata of Townsend Deprivation Index: 2 strata at the group level, shown on the left, and 5 equal-sized strata at the individual level, shown on the right panels. Within each stratum, we then performed the same analysis of prediction accuracy as a function of genetic distance as in Fig. 2 and Supplementary Fig. 3. At the group level, we measured prediction accuracy with the squared partial correlation between the PGS and the trait value in 250 bins of 278 individuals each. At the individual level, we measured prediction accuracy as the squared difference between the predicted phenotype and the true phenotype value. At both the group and individual levels, y-axis values show relative prediction accuracy, i.e. prediction accuracy divided by a baseline value. The baseline value is the mean prediction accuracy in 25 bins with median genetic distances that are closest to the mean genetic distance for GWAS individuals. Curves show cubic spline fits, with 8 knots placed based on the density of data points. Top represents those who are more deprived. For B and D, when plotting the spline fit on a logarithmic scale, we replaced the non-positive values with 10^{-6}. For all panels, no repeated measurements from the same individual were taken.

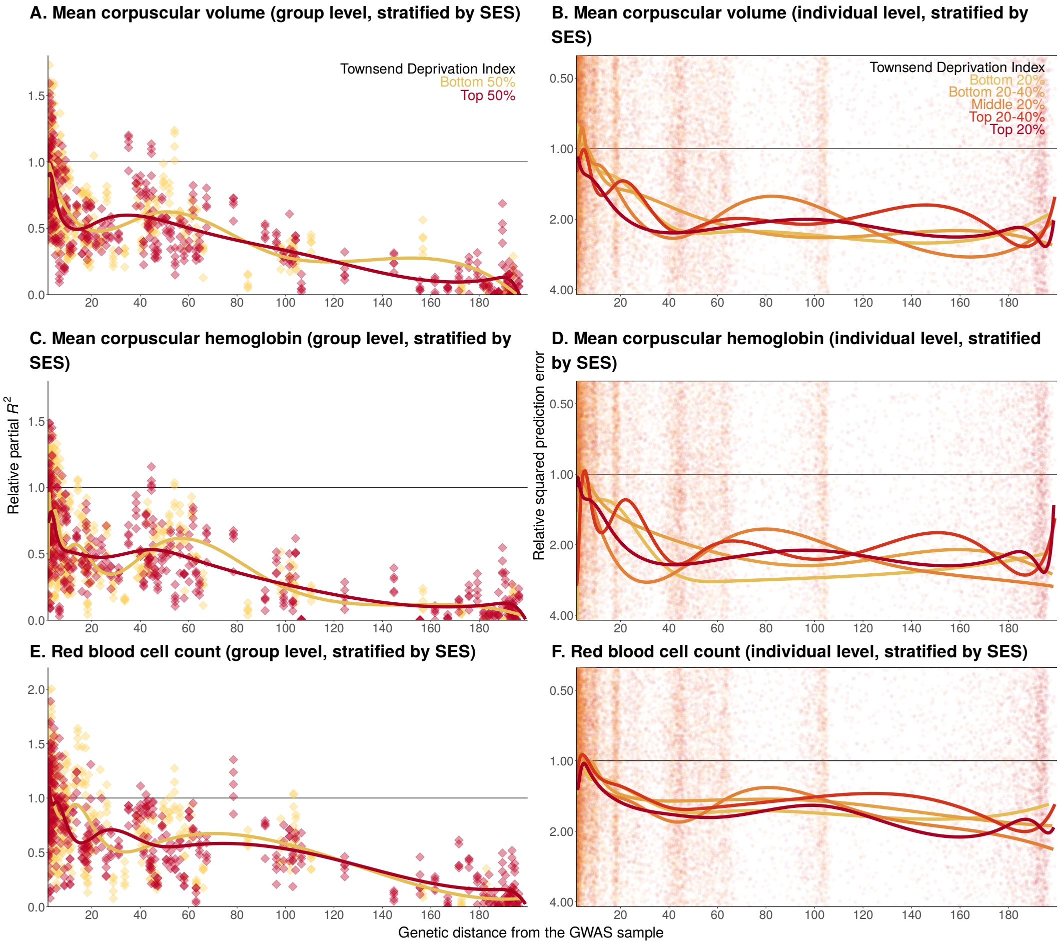

Supplementary Figure 69: Portability, as a function of both genetic distance and Townsend Deprivation Index, for red blood cell-related traits. We first stratified the prediction sample individuals into equal-sized strata of Townsend Deprivation Index: 2 strata at the group level, shown on the left, and 5 equal-sized strata at the individual level, shown on the right panels. Within each stratum, we then performed the same analysis of prediction accuracy as a function of genetic distance as in Fig. 2 and Supplementary Fig. 4. At the group level, we measured prediction accuracy with the squared partial correlation between the PGS and the trait value in 250 bins of 278 individuals each. At the individual level, we measured prediction accuracy as the squared difference between the predicted phenotype and the true phenotype value. At both the group and individual levels, y-axis values show relative prediction accuracy, i.e. prediction accuracy divided by a baseline value. The baseline value is the mean prediction accuracy in 25 bins with median genetic distances that are closest to the mean genetic distance for GWAS individuals. Curves show cubic spline fits, with 8 knots placed based on the density of data points. Top represents those who are more deprived. For all panels, no repeated measurements from the same individual were taken.

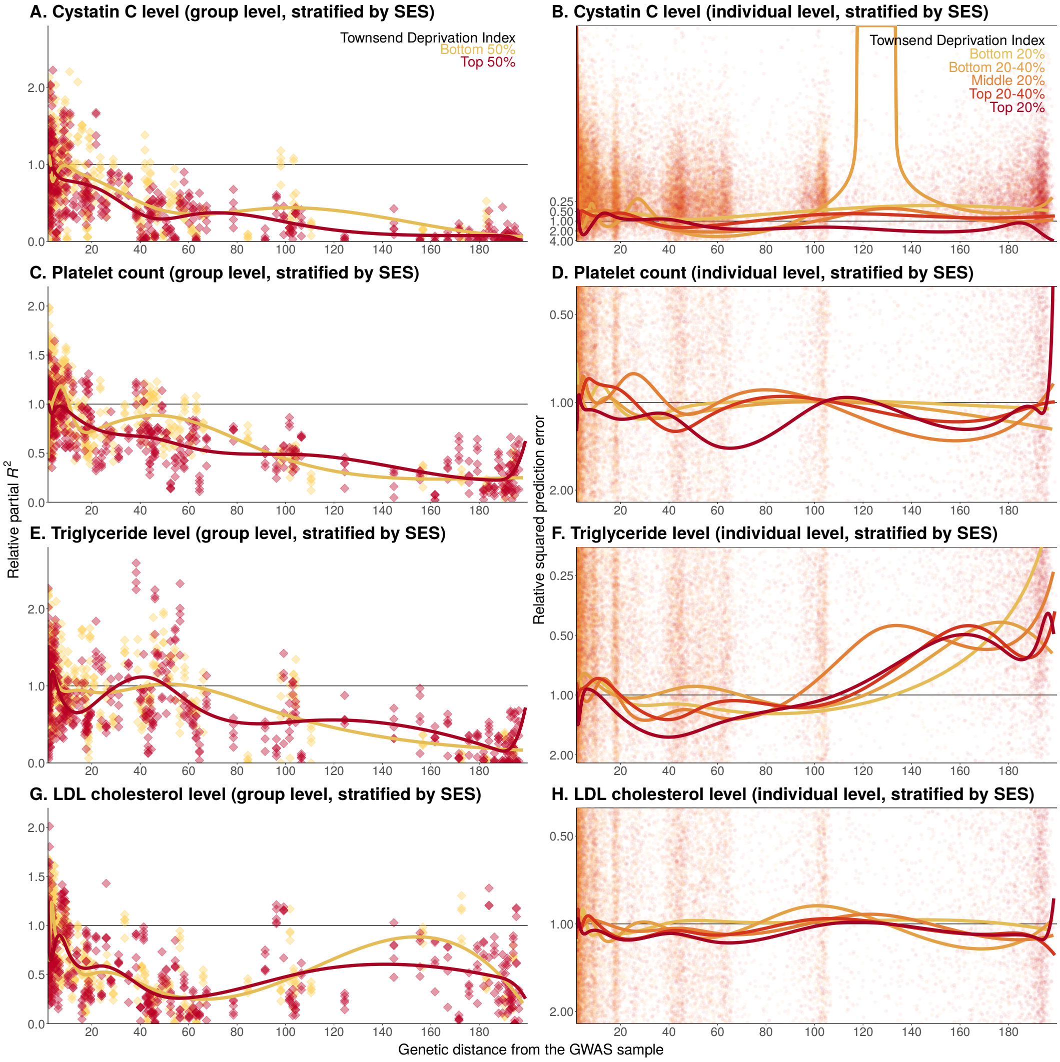

Supplementary Figure 70: Portability, as a function of both genetic distance and Townsend Deprivation Index, for other biomarkers. We first stratified the prediction sample individuals into equal-sized strata of Townsend Deprivation Index: 2 strata at the group level, shown on the left, and 5 equal-sized strata at the individual level, shown on the right panels. Within each stratum, we then performed the same analysis of prediction accuracy as a function of genetic distance as in Fig. **??** and Supplementary Fig. 5. At the group level, we measured prediction accuracy with the squared partial correlation between the PGS and the trait value in 250 bins of 278 individuals each. At the individual level, we measured prediction accuracy as the squared difference between the predicted phenotype and the true phenotype value. At both the group and individual levels, y-axis values show relative prediction accuracy, i.e. prediction accuracy divided by a baseline value. The baseline value is the mean prediction accuracy in 25 bins with median genetic distances that are closest to the mean genetic distance for GWAS individuals. Curves show cubic spline fits, with 8 knots placed based on the density of data points. Top represents those who are more deprived. For B, when plotting the spline fit on a logarithmic scale, we replaced the non-positive values with 10^{-6}. For all panels, no repeated measurements from the same individual were taken.

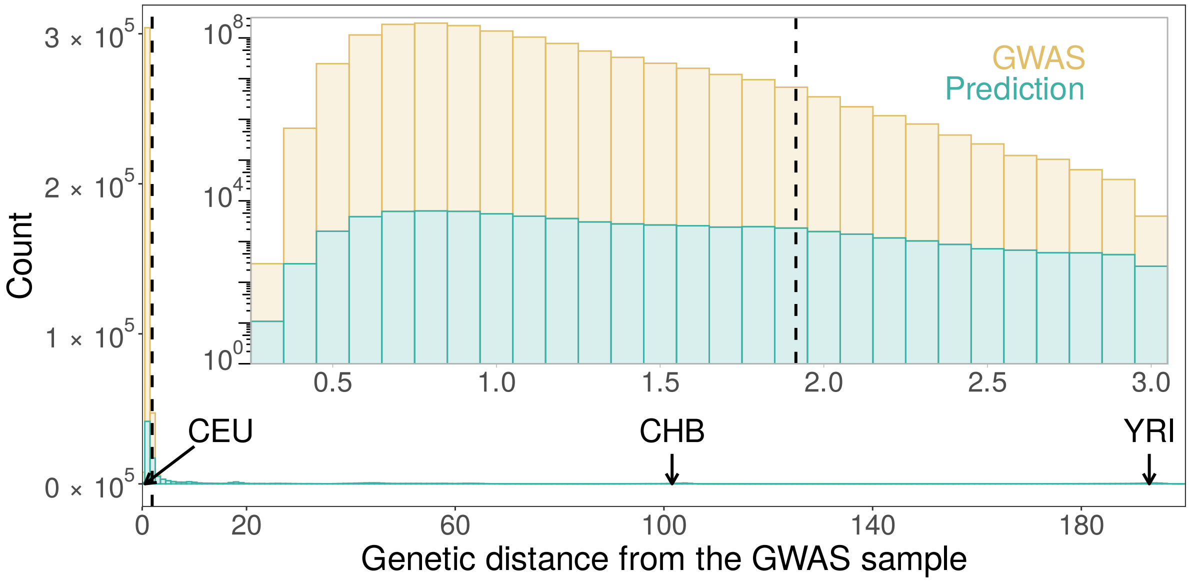

Supplementary Figure 71: Measuring genetic distance from the GWAS sample, with White British individuals included in the prediction set. This figure mirrors main text Fig. 1C, but reflects different compositions of the GWAS sample and the prediction sample than the ones used for the main text analysis. Here, after filtering, from a total of 336,923 WB individuals, we randomly selected 300,000 as the GWAS set, and used the remaining 106,423 individuals as the prediction set (36,923 WB and 69,500 NWB). We followed the same procedures as described in Methods, and identified that across 10,000 individuals from the prediction set, genetic distance to the GWAS sample (calculated with 40 PCs) is highly correlated with F_{st} between the GWAS sample and the individual. For reference, we show the mean genetic distances for subsets of the 1000 Genomes dataset[12]: CEU, Utah residents of primarily Northern and Western European descent; CHB, Han Chinese in Beijing, China; YRI, Yoruba in Ibadan, Nigeria. The dashed line represents the 97.5th percentile of genetic distance from among GWAS sample individuals. The inset is a zoomed-in view of genetic distances up to 3.0, on a log scale, to better visualize the distribution of the new GWAS sample. For all panels, no repeated measurements from the same individual were taken.

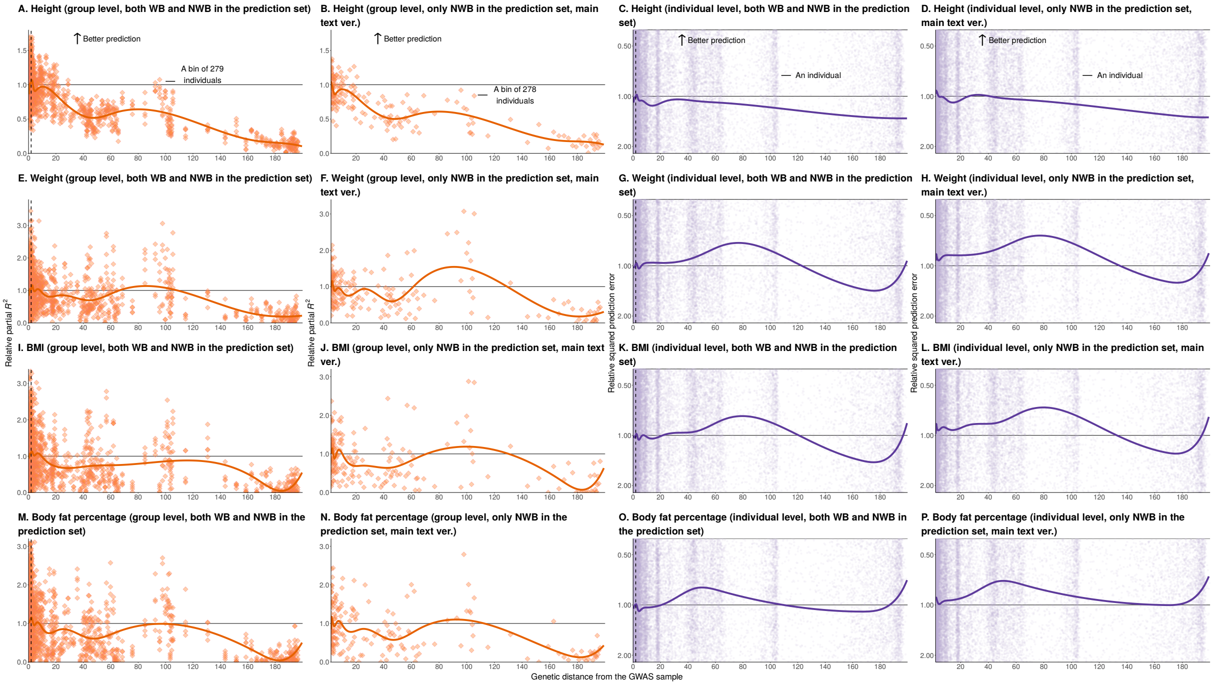

Supplementary Figure 72: A comparison of portability trends, with different choices of the GWAS and prediction samples, for anthropometric measurements. This figure presents the same analysis as Fig. 2 and Supplementary Fig. 2, but the first and third columns (A, C, E, G, I, K, M, O) show results obtained with a smaller GWAS set of 300,000 individuals labeled by the UKB as White British (WB) and a larger prediction set of 36,923 WB and 69,500 additional individuals not labeled as White British (NWB). The second and fourth columns (B, D, F, H, J, L, N, P) show results obtained with a GWAS set of 336,923 WB and a prediction set of 69,500 NWB only. At the group level (left two columns), we measured prediction accuracy with the squared partial correlation between the PGS and the trait value, in 382 bins of 279 individuals each in the first column and 250 bins of 278 individuals each in the second column. At the individual level (right two columns), we measured prediction accuracy as the squared difference between the predicted phenotype and the true phenotype value. At both the group and individual levels, y-axis values show relative prediction accuracy, i.e. prediction accuracy divided by a baseline value. The baseline value is the mean prediction accuracy in 38 bins with median genetic distances that are closest to the mean genetic distance for GWAS individuals. Curves show cubic spline fits, with 8 knots placed based on the density of data points. For all panels, no repeated measurements from the same individual were taken.

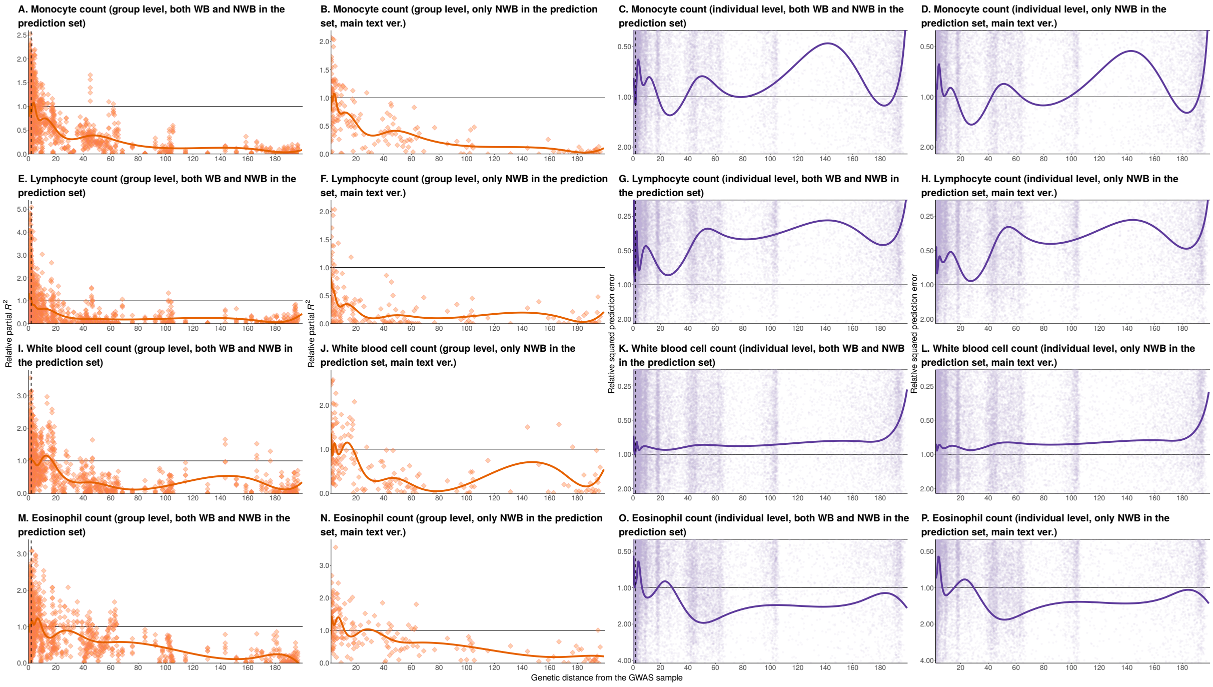

Supplementary Figure 73: A comparison of portability trends, with different choices of the GWAS and prediction samples, for white blood cell-related traits. This figure presents the same analysis as Fig. 2 and Supplementary Fig. 3, but the first and third columns (A, C, E, G, I, K, M, O) show results obtained with a smaller GWAS set of 300,000 individuals labeled by the UKB as White British (WB) and a larger prediction set of 36,923 WB and 69,500 additional individuals not labeled as White British (NWB). The second and fourth columns (B, D, F, H, J, L, N, P) show results obtained with a GWAS set of 336,923 WB and a prediction set of 69,500 NWB only. At the group level (left two columns), we measured prediction accuracy with the squared partial correlation between the PGS and the trait value, in 382 bins of 279 individuals each in the first column and 250 bins of 278 individuals each in the second column. At the individual level (right two columns), we measured prediction accuracy as the squared difference between the predicted phenotype and the true phenotype value. At both the group and individual levels, y-axis values show relative prediction accuracy, i.e. prediction accuracy divided by a baseline value. The baseline value is the mean prediction accuracy in 38 bins with median genetic distances that are closest to the mean genetic distance for GWAS individuals. Curves show cubic spline fits, with 8 knots placed based on the density of data points. For all panels, no repeated measurements from the same individual were taken.

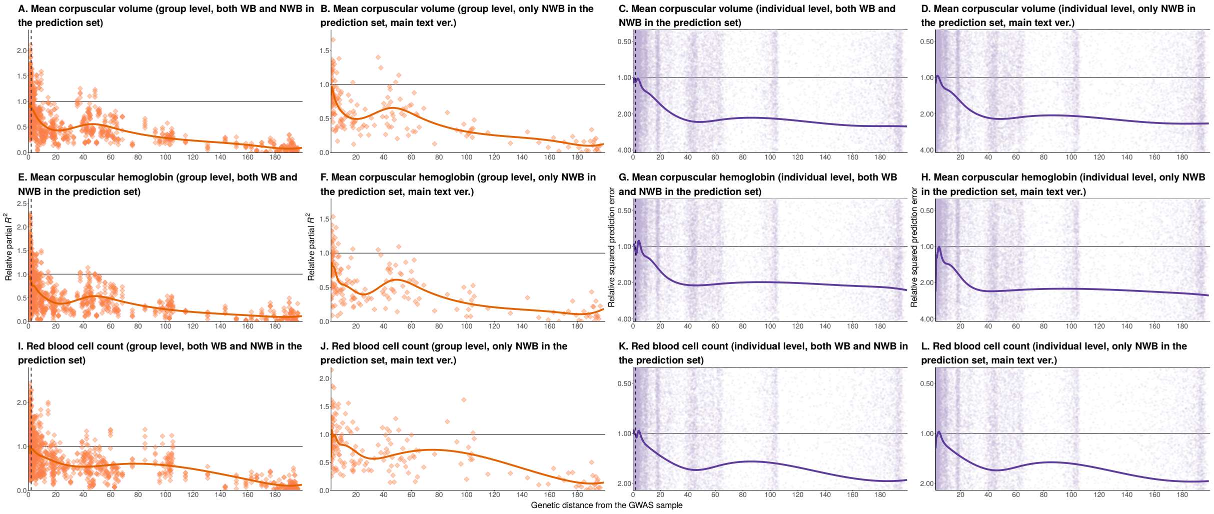

Supplementary Figure 74: A comparison of portability trends, with different choices of the GWAS and prediction samples, for red blood cell-related traits. This figure presents the same analysis as Fig. 2 and Supplementary Fig. 4, but the first and third columns (A, C, E, G, I, K) show results obtained with a smaller GWAS set of 300,000 individuals labeled by the UKB as White British (WB) and a larger prediction set of 36,923 WB and 69,500 additional individuals not labeled as White British (NWB). The second and fourth columns (B, D, F, H, J, L) show results obtained with a GWAS set of 336,923 WB and a prediction set of 69,500 NWB only. At the group level (left two columns), we measured prediction accuracy with the squared partial correlation between the PGS and the trait value, in 382 bins of 279 individuals each in the first column and 250 bins of 278 individuals each in the second column. At the individual level (right two columns), we measured prediction accuracy as the squared difference between the predicted phenotype and the true phenotype value. At both the group and individual levels, y-axis values show relative prediction accuracy, i.e. prediction accuracy divided by a baseline value. The baseline value is the mean prediction accuracy in 38 bins with median genetic distances that are closest to the mean genetic distance for GWAS individuals. Curves show cubic spline fits, with 8 knots placed based on the density of data points. For all panels, no repeated measurements from the same individual were taken.

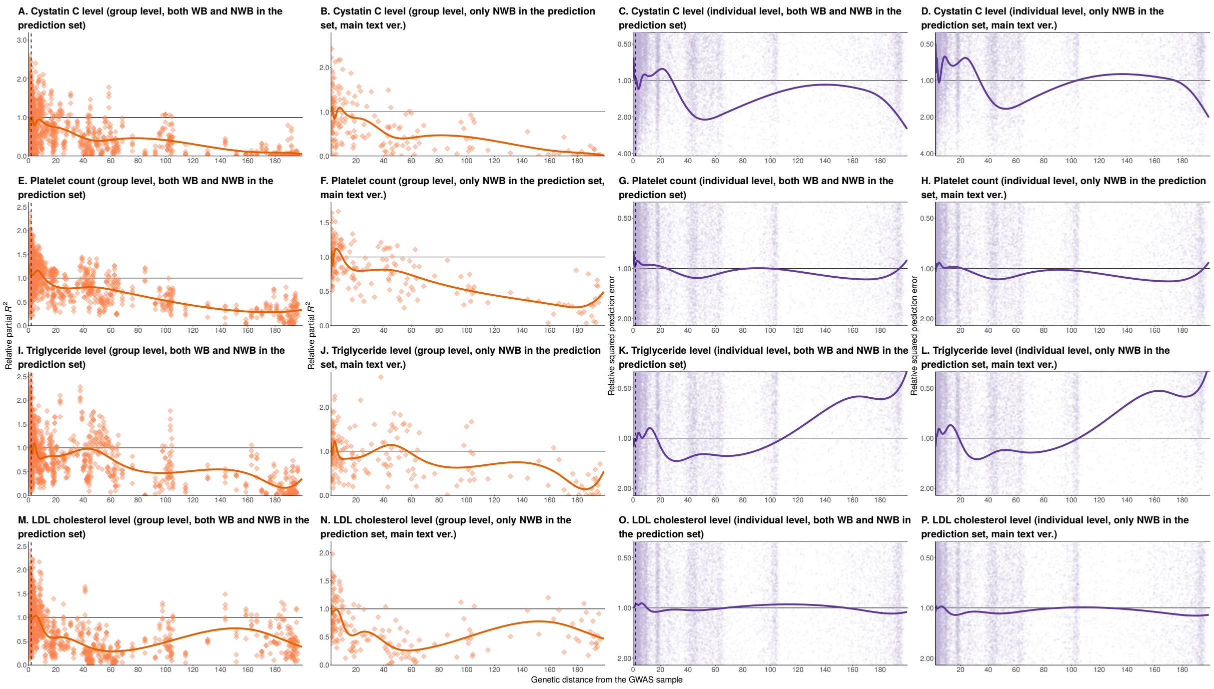

Supplementary Figure 75: A comparison of portability trends, with different choices of the GWAS and prediction samples, for other biomarkers. This figure presents the same analysis as Fig. 2 and Supplementary Fig. 5, but the first and third columns (A, C, E, G, I, K, M, O) show results obtained with a smaller GWAS set of 300,000 individuals labeled by the UKB as White British (WB) and a larger prediction set of 36,923 WB and 69,500 additional individuals not labeled as White British (NWB). The second and fourth columns (B, D, F, H, J, L, N, P) show results obtained with a GWAS set of 336,923 WB and a prediction set of 69,500 NWB only. At the group level (left two columns), we measured prediction accuracy with the squared partial correlation between the PGS and the trait value, in 382 bins of 279 individuals each in the first column and 250 bins of 278 individuals each in the second column. At the individual level (right two columns), we measured prediction accuracy as the squared difference between the predicted phenotype and the true phenotype value. At both the group and individual levels, y-axis values show relative prediction accuracy, i.e. prediction accuracy divided by a baseline value. The baseline value is the mean prediction accuracy in 38 bins with median genetic distances that are closest to the mean genetic distance for GWAS individuals. Curves show cubic spline fits, with 8 knots placed based on the density of data points. For all panels, no repeated measurements from the same individual were taken.

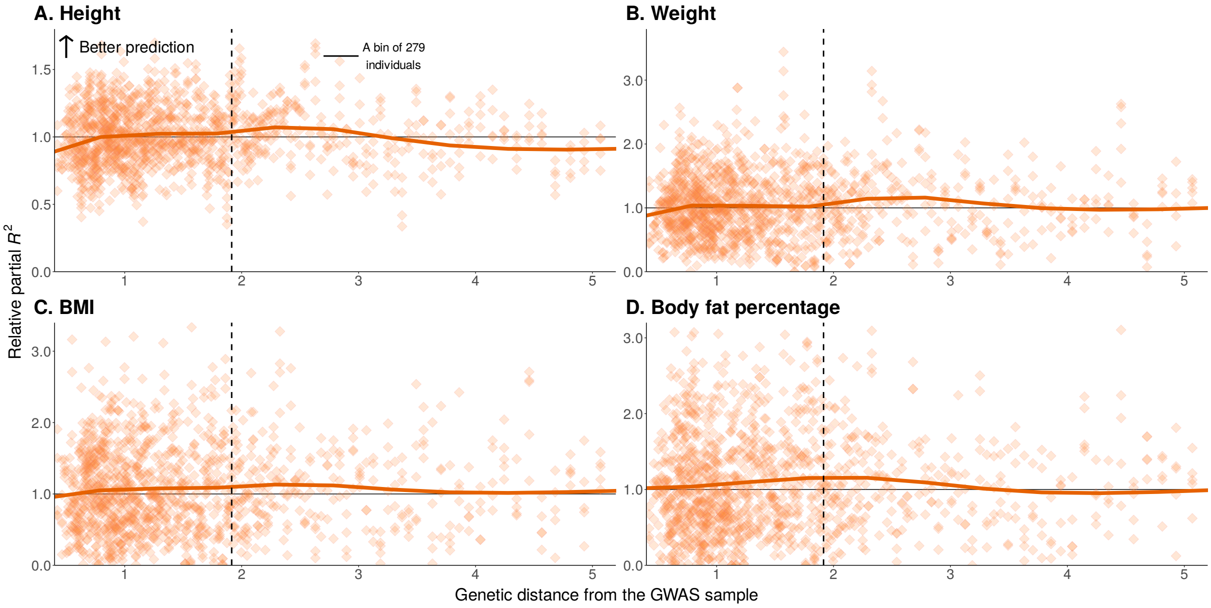

Supplementary Figure 76: Zoomed-in portability trends, with WB in the prediction set, for anthropometric measurements. This figure is a zoomed-in version of Supplementary Fig. 72A, E, I, M, to better visualize the bins at small genetic distances (x-axis shows genetic distance up to 5.2). The results are obtained from a GWAS set of 300,000 WB and a prediction set of both WB and NWB (36,923 WB and 69,500 NWB). We measured prediction accuracy with the squared partial correlation between the PGS and the trait value, in 382 bins of 279 individuals each. Y-axis values show relative prediction accuracy, i.e. prediction accuracy divided by a baseline value. The baseline value is the mean prediction accuracy in 38 bins with median genetic distances that are closest to the mean genetic distance for GWAS individuals. Curves show cubic spline fits, with 8 knots placed based on the density of data points. For all panels, no repeated measurements from the same individual were taken.

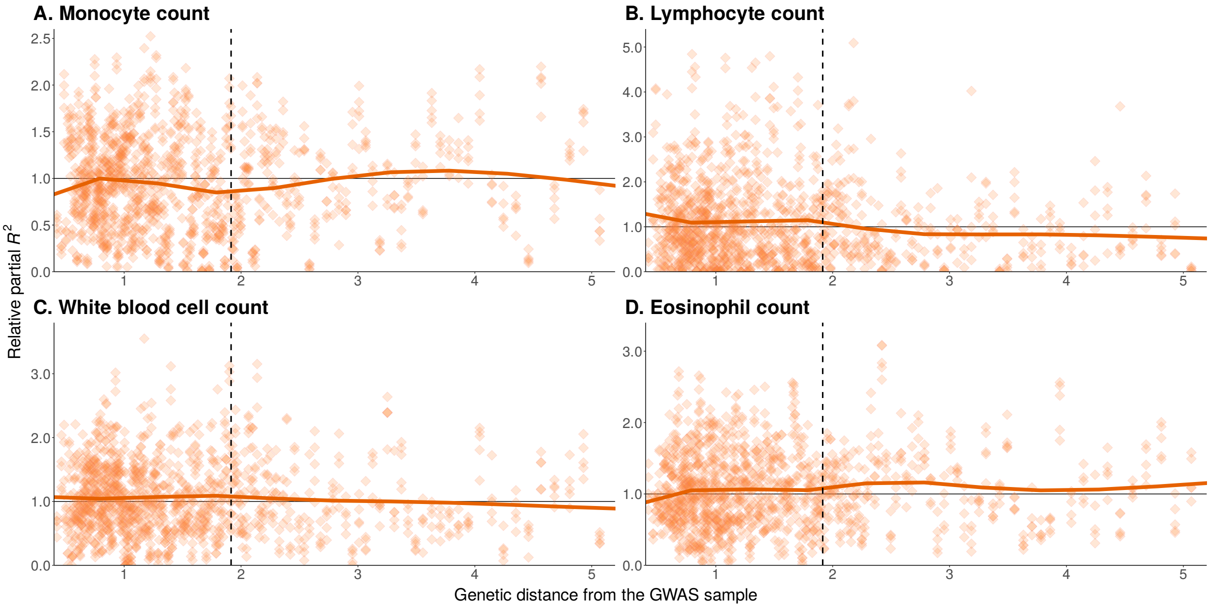

Supplementary Figure 77: Zoomed-in portability trends, with WB in the prediction set, for white blood cell-related traits. This figure is a zoomed-in version of Supplementary Fig. 73A, E, I, M, to better visualize the bins at small genetic distances (x-axis shows genetic distance up to 5.2). The results are obtained from a GWAS set of 300,000 WB and a prediction set of both WB and NWB (36,923 WB and 69,500 NWB). We measured prediction accuracy with the squared partial correlation between the PGS and the trait value, in 382 bins of 279 individuals each. Y-axis values show relative prediction accuracy, i.e. prediction accuracy divided by a baseline value. The baseline value is the mean prediction accuracy in 38 bins with median genetic distances that are closest to the mean genetic distance for GWAS individuals. Curves show cubic spline fits, with 8 knots placed based on the density of data points. For all panels, no repeated measurements from the same individual were taken.

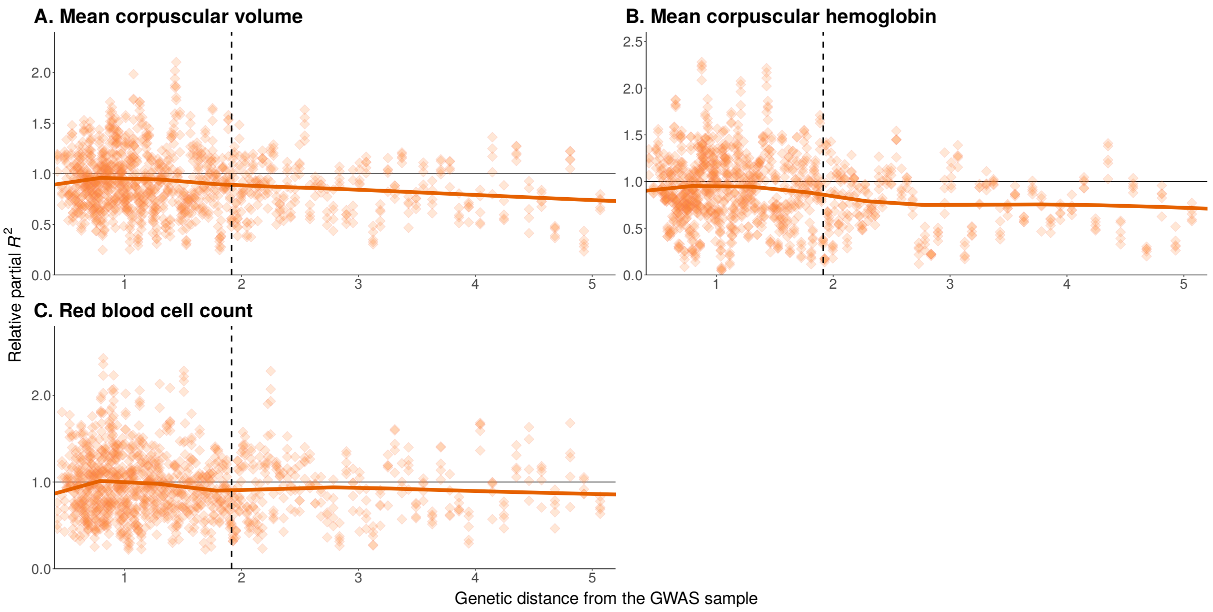

Supplementary Figure 78: Zoomed-in portability trends, with WB in the prediction set, for red blood cell-related traits. This figure is a zoomed-in version of Supplementary Fig. 74A, E, I, to better visualize the bins at small genetic distances (x-axis shows genetic distance up to 5.2). The results are obtained from a GWAS set of 300,000 WB and a prediction set of both WB and NWB (36,923 WB and 69,500 NWB). We measured prediction accuracy with the squared partial correlation between the PGS and the trait value, in 382 bins of 279 individuals each. Y-axis values show relative prediction accuracy, i.e. prediction accuracy divided by a baseline value. The baseline value is the mean prediction accuracy in 38 bins with median genetic distances that are closest to the mean genetic distance for GWAS individuals. Curves show cubic spline fits, with 8 knots placed based on the density of data points. For all panels, no repeated measurements from the same individual were taken.

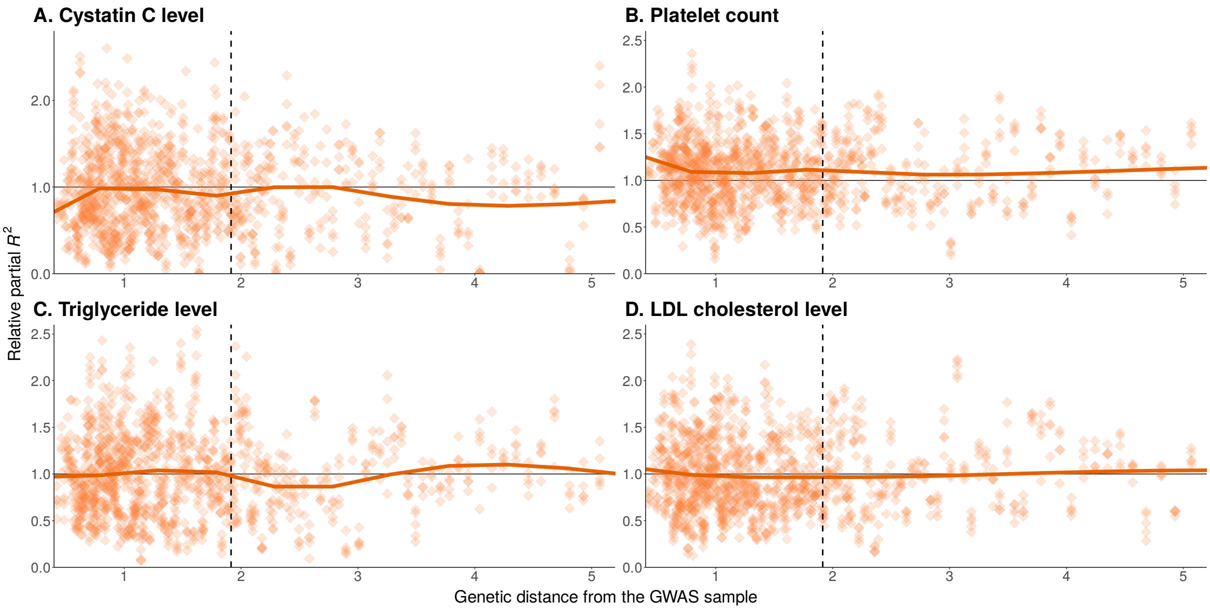

Supplementary Figure 79: Zoomed-in portability trends, with WB in the prediction set, for other biomarkers. This figure is a zoomed-in version of Supplementary Fig. 75A, E, I, M, to better visualize the bins at small genetic distances (x-axis shows genetic distance up to 5.2). The results are obtained from a GWAS set of 300,000 WB and a prediction set of both WB and NWB (36,923 WB and 69,500 NWB). We measured prediction accuracy with the squared partial correlation between the PGS and the trait value, in 382 bins of 279 individuals each. Y-axis values show relative prediction accuracy, i.e. prediction accuracy divided by a baseline value. The baseline value is the mean prediction accuracy in 38 bins with median genetic distances that are closest to the mean genetic distance for GWAS individuals. Curves show cubic spline fits, with 8 knots placed based on the density of data points. For all panels, no repeated measurements from the same individual were taken.

Supplementary references

[1] Townsend, P. Deprivation. *Journal of Social Policy* **16**, 125–146 (1987).

[2] Mbatchou et al. Computationally efficient whole-genome regression for quantitative and binary traits. *Nature Genetics* **53**, 1097–1103 (2021).

[3] Ge, T., Chen, C.-Y., Ni, Y., Feng, Y.-C. A. & Smoller, J. W. Polygenic prediction via Bayesian regression and continuous shrinkage priors. *Nature Communications* **10**, 1776 (2019).

[4] Python Software Foundation. Python Language Reference. `https://www.python.org` (2019). Version 3.8.1.

[5] Young, A. I., Benonisdottir, S., Przeworski, M. & Kong, A. Deconstructing the sources of genotype-phenotype associations in humans. *Science* **365**(6460), 1396–1400 (2019).

[6] Privé, F., Luu, K., Blum, M. G., McGrath, J. J. & Vilhjálmsson, B. J. Efficient toolkit implementing best practices for principal component analysis of population genetic data. *Bioinformatics* **36**(16), 4449–4457 (2020).

[7] Smith et al. A Litmus Test for Confounding in Polygenic Scores. Preprint at `https://www.biorxiv.org/content/10.1101/2025.02.01.635985v1` (2025).

[8] R Core Team. R: A Language and Environment for Statistical Computing. `https://www.R-project.org` (2024). Version 4.4.1.

[9] Wood, S. N. mgcv: Mixed GAM Computation Vehicle with Automatic Smoothness Estimation. `https://CRAN.R-project.org/package=mgcv` (2024). Version 1.9-1.

[10] Neale Lab. UK Biobank. `https://www.nealelab.is/uk-biobank` (2018).

[11] Purcell, S. & Chang, C. PLINK 1.9. `https://www.cog-genomics.org/plink/1.9` (2023). Version v1.90b7.2 64-bit.

[12] Auton et al. A global reference for human genetic variation. *Nature* **526**, 68–74 (2015).
